# Engineering antiviral immune-like systems for autonomous virus detection and inhibition in mice

**DOI:** 10.1038/s41467-022-35425-9

**Published:** 2022-12-09

**Authors:** Yidan Wang, Ying Xu, Chee Wah Tan, Longliang Qiao, Wan Ni Chia, Hongyi Zhang, Qin Huang, Zhenqiang Deng, Ziwei Wang, Xi Wang, Xurui Shen, Canyu Liu, Rongjuan Pei, Yuanxiao Liu, Shuai Xue, Deqiang Kong, Danielle E. Anderson, Fengfeng Cai, Peng Zhou, Lin-Fa Wang, Haifeng Ye

**Affiliations:** 1 Shanghai Frontiers Science Center of Genome Editing and Cell Therapy, Biomedical Synthetic Biology Research Centre, Shanghai Key Laboratory of Regulatory Biology, Institute of Biomedical Sciences and School of Life Sciences, East China Normal University, Dongchuan Road 500, Shanghai, 200241 China; 2 Chongqing Key Laboratory of Precision Optics, Chongqing Institute of East China Normal University, Chongqing, 401120 China; 3 Programme in Emerging Infectious Diseases, Duke-NUS Medical School, Singapore, Singapore; 4 Department of Breast Surgery, Yangpu Hospital, School of Medicine, Tongji University, 450 Tengyue Road, Shanghai, 200090 China; 5 CAS Key Laboratory of Special Pathogens, Wuhan Institute of Virology, Chinese Academy of Sciences, Wuhan, 430071 Hubei China; 6 University of Chinese Academy of Sciences, Beijing, China; 7 State Key Laboratory of Virology, Wuhan Institute of Virology, Chinese Academy of Sciences, Wuhan, 430071 Hubei China; 8 Department of Biosystems Science and Engineering, ETH Zurich, CH-4058 Basel, Switzerland; 9 SingHealth Duke-NUS Global Health Institute, Singapore, Singapore

**Keywords:** Gene therapy, Synthetic biology

## Abstract

The ongoing COVID-19 pandemic has demonstrated that viral diseases represent an enormous public health and economic threat to mankind and that individuals with compromised immune systems are at greater risk of complications and death from viral diseases. The development of broad-spectrum antivirals is an important part of pandemic preparedness. Here, we have engineer a series of designer cells which we term autonomous, intelligent, virus-inducible immune-like (ALICE) cells as sense-and-destroy antiviral system. After developing a destabilized STING-based sensor to detect viruses from seven different genera, we have used a synthetic signal transduction system to link viral detection to the expression of multiple antiviral effector molecules, including antiviral cytokines, a CRISPR-Cas9 module for viral degradation and the secretion of a neutralizing antibody. We perform a proof-of-concept study using multiple iterations of our ALICE system in vitro, followed by in vivo functionality testing in mice. We show that dual output ALICE_SaCas9+Ab_ system delivered by an AAV-vector inhibited viral infection in herpetic simplex keratitis (HSK) mouse model. Our work demonstrates that viral detection and antiviral countermeasures can be paired for intelligent sense-and-destroy applications as a flexible and innovative method against virus infection.

## Abstract (web summary)

The development of broad-spectrum antivirals is an important part of pandemic preparedness and response. Here the authors present ALICE, synthetic biology designer immune-like cells that act as a sense-and-destroy antiviral system can detect viruses from seven different genera, mimicking the human innate immune system.

## Introduction

With global climate change, we will confront more emerging and reemerging human diseases^1^ and the lack of effective antivirals is taking a toll on human lives and social wealth^2^. As part of pandemic preparedness, there is an urgent need to establish effective antiviral strategies against “Disease X”^2^. The human innate immune response is the first line of defense against viral pathogens^3^, but there are vast differences in the efficacy of innate immune functions between individuals, especially immunocompromised individuals who are 2–30 times more likely to contract cytomegalovirus (CMV), hepatitis B virus (HBV), and herpes simplex virus types 1 and 2 (HSV-1 and HSV-2) infection^4^. HSV-1 infection can cause a wide variety of diseases, including herpetic simplex keratitis (HSK), which has a high mortality rate if untreated^5^. Generally, HSK is caused by HSV-1 infection in the cornea, which is a leading cause of blindness and viral encephalitis in the developed world^6^. Early recognition of viruses is initiated by STING [stimulator of interferon (IFN) genes] in immune cells. STING recognizes double-stranded DNA or RNA released from an invading virus^7,8^ and results in the translocation of transcription factor IRF3 and the production of chemokines and proinflammatory cytokines that recruit phagocytes to the site of infection to eliminate virus in mammalian cells^9^.

Synthetic biology-based molecular diagnostics have been created by engineering natural biological components for practical applications^10–12^. Programmable RNA sensors called toehold switches were designed to bind and sense virtually any RNA sequence and can be used to detect viruses^13^. The freeze-dried, paper-based, cell-free protein expression platform allows for the deployment of the toehold switch sensor for virus detection outside of a research laboratory anytime, anywhere, and at room temperature^14,15^.

The ongoing biological revolution stemming from the discovery of clustered regularly interspaced short palindromic repeats (CRISPR) has led to the development of nucleic acid-based pathogen detection technologies, notably the CRISPR-based diagnostic tools [SHERLOCK^16,17^, DETECTR^18^, and HOLMES^19^], which were based on the collateral effect of an RNA-guided and RNA-targeting CRISPR effector Cas13 or Cas12a. These technologies are of high sensitivity and specificity in the detection of target-specific virus^20^.

In addition to these CRISPR-based detection technologies, CRISPR-associated protein 9 (Cas9)-nuclease-based technologies have been developed to degrade virus genomic materials with demonstrated in vitro potential with SARS-CoV-2^21^, influenza^21^, human immunodeficiency virus (HIV)^22^, HBV^23^, human papillomavirus (HPV)^24^, and HSV-1^25^. However, the delivery of Cas9 using a lentiviral- or adeno-associated virus (AAV)-vector driven by constitutive promoters can lead to sustained Cas9 expression, anti-Cas9 immune responses, and off-target editing, which have halted the use of these technologies as antiviral therapies in clinical applications^26^. It is worth to note that the constitutive expression of Cas9 can increase the risk of the emergence of CRISPR/Cas9-resistant escape mutant virus strains^25^.

Recent advances in both our understanding of immunology and insights from synthetic biology about cellular engineering have led to the technological development of “immune-like designer cells”, including a self-regulated designer cell that can prevent methicillin-resistant *Staphylococcus aureus* infection^27^. Thus, there is hope for offering better clinical care to immunocompromised patients based on the development and deployment of smart cells that can compensate for inoperative or weak immune responses by acting as autonomous and potentially broad-spectrum antiviral agents.

Our research focuses on engineering antiviral immune-like systems for biomedical applications, and we envisioned that it is possible to combine viral detection and antiviral function in the same engineered cell, potentially with virus infection-triggered conditional expression of antiviral cytokines (IFN-α and IFN-β), the Cas9/sgRNA (small guide RNA) complex and even broadly-neutralizing antibodies. Such engineered intelligent “sense-and-destroy” immune-like cells should be able to autonomously detect the presence of a viral pathogen and respond with targeted antivirals. Here we report the development and deployment of autonomous, intelligent, virus-inducible immune-like (ALICE) systems. Functional ALICE cells include a closed-loop gene network comprising a destabilized version of the STING immune signal pathway sensor for the detection of viruses that can activate STING-based signaling, a synthetic virus-inducible promoter to drive the expression of antiviral cytokines (ALICE_im_), and orthogonal mechanisms of antiviral activity including a Cas9-based degradation module (ALICE_Cas9_) and a neutralizing antibody (Ab) module (ALICE_Ab_). We demonstrate that the ALICE system can monitor the cellular environment for the presence of viral pathogens and show that ALICE cells selectively induce antiviral responses upon the detection of a virus. ALICE_im_ has the ability to enhance the host immune response and relative broad-spectrum antiviral activity due to the inducible expression of antiviral cytokines. Following proof-of-concept experiments in vitro using multiple variations of the basic ALICE system, we applied our technology in vivo in mouse models and show that the ALICE systems efficiently blocked HSV-1 replication and spread in mice at different stages of viral infection. Additionally, the dual Cas9 and neutralizing antibody system (ALICE_SaCas9+Ab_) delivered by an AAV-vector could eliminate viruses via retrograde transport from corneas to trigeminal ganglia (TG) in HSK mice. Taken together, the ALICE systems might have the potential to combat refractory virus infectious diseases.

## Results

### Design and validation of an autonomous, intelligent, virus-inducible immune-like sensor (ALICE_sen_)

**Fig. 1:**
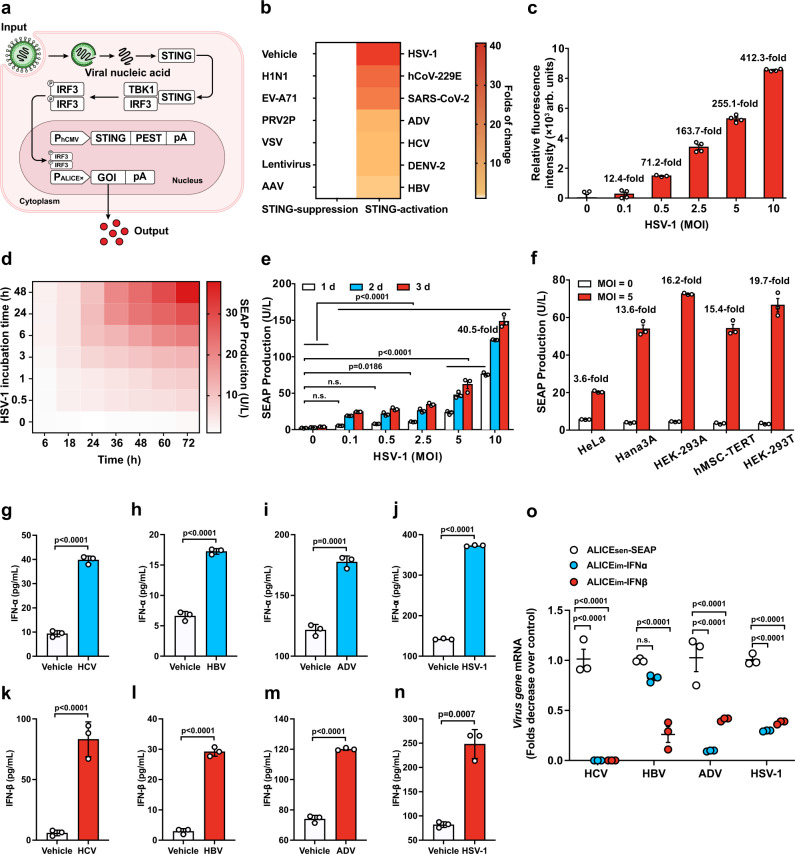
Design and characterization of an autonomous, intelligent, virus-inducible immune-like sensor (ALICE_sen_). **a** Schematic illustration of the design principle for a virus sensor (ALICE_sen_). Virus infects mammalian cells and releases its nucleic acid into the cytoplasm, which activates the destabilized STING (pYW274). Activated STING triggers a synthetic immune signaling pathway mediated by endogenous tank-binding kinase 1 (TBK1), resulting in activating phosphorylation and dimerization of endogenous interferon regulatory factor 3 (IRF3), which translocates into the nucleus as a dimer and initiates the expression of a given gene of interest (GOI) under the control of a “virus-inducible promotor (P_ALICE×_)” sequence. **b** Fold change of SEAP in ALICE_sen_ induced by different STING-suppression/activation viruses. pYW274/pWS67-transgenic cells were incubated with different viruses as indicated, and SEAP in supernatant was quantified at 2 and 4 dpi (day post-infection). **c** HSV-1-dependent EGFP expression in ALICE_sen_. pYW274/pYW379-transgenic HEK-293T cells were incubated with different titers of HSV-1 (MOI = 0–10) and quantified at 2 dpi. **d** Time-dependent SEAP production kinetics of ALICE_sen_. HEK_ALICE-SEAP_ stable cell lines were incubated with HSV-1 (MOI = 0.5) for different incubation times. SEAP levels in culture supernatants were quantified at the indicated time points. **e** HSV-1-dependent SEAP production kinetics of ALICE_sen_. pYW274/pWS67-transgenic HEK-293T cells were incubated with different titers of HSV-1. White bars (1 day), blue bars (2 day), red bar (3 day). *P* values for all other groups versus HSV-1 (MOI = 0) group on the same day. **f** HSV-1-inducible SEAP production in various mammalian cell lines. **g**–**n** Virus-inducible IFN-α/IFN-β production in ALICE_im_. *P* values for virus group versus Vehicle group. **o** qPCR analysis of viral genes in ALICE_im_. White circles (ALICE_sen_-SEAP), blue circles (ALICE_im_-IFNα), red circles (ALICE_im_-IFNβ). *P* values for all other groups versus ALICE_sen_-SEAP group in the same virus. Data in b-o are expressed as means ± SD; *n* = 3 independent experiments in **b**, **d**–**o**; *n* = 3 or 4 independent experiments in (**c**); *P* values in **e**, **o** were calculated by two-way ANOVA with Bonferroni’s post hoc test; *P* values in **g**–**n** were calculated by two-tailed unpaired *t*-test; n.s. not significant. Source data are provided as a Source Data file.

The central component of our antiviral platform requires a sensitive sensor to detect viral nucleic acid. As a first trial, we designed an autonomous, intelligent, virus-inducible immune-like sensor (ALICE_sen_) in which the destabilized STING protein is ectopically expressed in mammalian cells to function as the initial sensor molecule to detect the presence of intracellular exogenous dsDNA or RNA. The activation of destabilized STING then recruits TANK-binding kinase 1 (TBK1) and traffics from the endoplasmic reticulum to a perinuclear endosomal compartment, leading to the phosphorylation and dimerization of IRF3. Subsequently, the phosphorylated and dimerized IRF3 translocates into the nucleus and binds to synthetic promoters (P_ALICE×_) positioned at genes of interest (GOI), thus enabling exogenous-viral nucleic acid-triggered transcriptional activation in mammalian cells (Fig. 1a).

To optimize a virus-responsive ALICE_sen_ iteration that achieves minimal basal transgene expression in the absence of virus and maximal induction ratio in the presence of virus, we used HSV-1 as a model virus, and initially utilized stable STING. Aligned with published data^28^, our immunoblotting assays of endogenous human proteins revealed no STING and cGAS expression in HEK-293T cells (Supplementary Fig. 1a). When exposing different amounts of stable STING, the maximal induction was (3.3-fold ± 0.26) (Supplementary Fig. 1b). To increase induction fold with reduced background, we fused a proteolytic tag (PEST) onto the C-terminus of the STING protein under the control of various promoters (Supplementary Fig. 1c). By promoting the proteolytic degradation of the STING protein, induction was increased to ~20-fold. We assessed the HSV-1-responsive ALICE_sen_ guided by the synthetic IRF3-specific or non-specific promoters (Supplementary Fig. 1d) in HEK-293T cells and found the combination of P_hCMV_–driven STING-PEST and P_ALICE6_, labeled as ALICE_sen_, showed the highest induction (~23-fold) of the reporter gene secreted alkaline phosphatase (SEAP) in the presence of HSV-1.

Importantly, ALICE_sen_ could also be activated by infection with STING-dependent pan-genus viruses^29–32^, including DENV-2, SARS-CoV-2, hCoV-229E, hepatitis C virus (HCV), HBV, adenovirus (ADV) and HSV-1 (Fig. 1b), indicating that ALICE_sen_ might be used as a relative broad-spectrum sensor to detect STING-dependent viruses. Meanwhile, infection by other STING-suppression viruses^32–37^ [e.g., influenza virus (H1N1), enterovirus 71 (EV-A71), pteropine reovirus-2 (PRV2P), vesicular stomatitis virus (VSV), lentivirus or adeno-associated virus (AAV)] failed to do so (Fig. 1b). ALICE_sen_ has the potential to aid in the understanding of the structural and mechanistic STING pathway against virus infection. The maximal induction (~412-fold) of ALICE_sen_ was reached with an enhanced green fluorescent protein (EGFP) reporter construct (Fig. 1c). ALICE_sen_ was functional in time and dosage-dependent manner (Fig. 1d, e). Moreover, we demonstrate that the ALICE_sen_ system was activated in five commonly used mammalian cell lines (Fig. 1f). Reversible induction kinetics of ALICE_sen_ was attained with the use of the antiviral drug acyclovir (ACV) (Supplementary Fig. 1e).

Type I interferons (IFN-α/β), involved in activation of innate immune signaling pathways, have broad-spectrum antiviral activity^38^. We engineered virus-inducible immune-like sense-and-clearance (ALICE_im_) cells, mimicking the human innate immune system, where downstream genes driven by the synthetic promoter (P_ALICE6_) were replaced with the human IFN-α or IFN-β. We next quantified virus-induced IFN-α and IFN-β production, and antiviral efficacy. In the presence of four different viruses (HCV, HBV, ADV, and HSV-1), ALICE_im_ cells can be induced to produce IFN-α (Fig. 1g–j) and IFN-β (Fig. 1k–n). The induced interferons can inhibit virus infection at the cellular level (Fig. 1o). These data demonstrate that ALICE_im_ acts as an artificial innate immune system protecting the host organism through nonspecific immune defense and surveillance by the induction of interferons.

### Design and validation of autonomous, intelligent, virus-inducible immune-like cells with a Cas9 protein (ALICE_Cas9_)

**Fig. 2:**
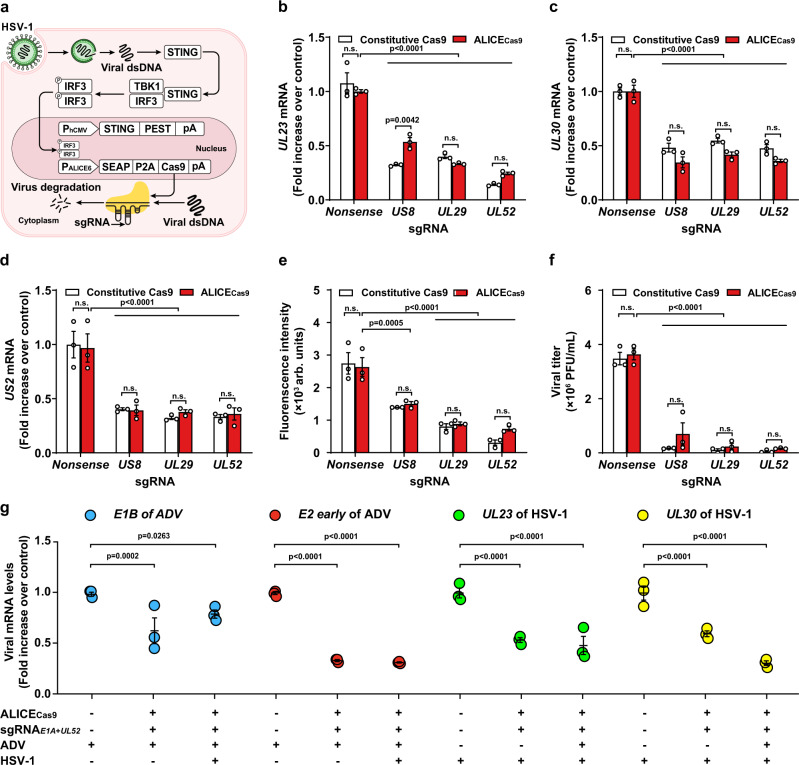
Design and validation of an autonomous, intelligent, antiviral, virus-inducible immune-like sense-and-deletion cells (ALICE_Cas9_ cells). **a** Schematic illustration of the design principle of ALICE_Cas9_ cells for autonomous sense-and-deletion of HSV-1. HSV-1 activates the virus sensor and initiates the expression of the Cas9 protein under the control of the virus-inducible promotor (P_ALICE6_). HSV-1-inducible Cas9 protein inhibits viral replication by targeting and deleting highly conserved virus replication-related genes *US8/UL29/UL52* under the guidance of its corresponding sgRNA. **b** qPCR analysis of mRNA levels of the viral transcript *UL23* from EGFP-labeled HSV-1. Transfection of HSV-1-targeting sgRNA [highly conserved virus replication related gene: *US8* locus (pYW102); *UL29* locus (pYW172); *UL52* locus (pYW188), and a nonsense control locus (pWS68)] with pYW274/pYW169 or constitutive Cas9 (pYW54) was performed 20 h prior to EGFP-labeled HSV-1 infection (MOI = 5) in HEK-293T cells. The relative mRNA expression of *UL23, UL30* (**c**), and *US2* (**d**) was quantified by qPCR. All data were respectively normalized to the *UL23, UL30, US2* gene expression levels in control group where HEK-293T cells co-transfected with pYW54/pWS68 were infected with EGFP-labeled HSV-1. White bars (Constitutive Cas9), red bars (ALICE_Cas9_) in (**b**–**f**). **e** Fluorescence intensity of ALICE_Cas9_. **f** EGFP-labeled HSV-1 titer in ALICE_Cas9_. **g** qPCR analysis of *E1B*/*E2 early* genes from ADV, and *UL23*/*UL30* genes from HSV-1 in ALICE_Cas9_ cells. ALICE_Cas9_ cells transfected with sgRNAs targeting *E1A* of ADV and *UL52* of HSV-1 were incubated with a single virus (ADV or HSV-1) or both viruses (ADV and HSV-1). Blue circles (*E1B* of ADV), red circles (*E2 early* of ADV), green circles (*UL23* of HSV-1), yellow circles (*UL30* of HSV-1). The data in **b**–**g** represent the means ± SD; *n* = 3 independent experiments; *P* values in **b**–**g** were calculated by two-way ANOVA with Bonferroni’s post hoc test; *P* values for all other groups versus *Nonsense* group in the same ALICE_Cas9_ group, and between Constitutive Cas9 group and ALICE_Cas9_ group in the same sgRNA; n.s. not significant. Source data are provided as a Source Data file.

Previous studies have utilized platforms for virus detection and elimination based on the CRISPR gene editing system^39,40^. Notably, in these systems the Cas9 protein is constitutively expressed prior to virus infection. Constitutive expression of nuclease protein Cas9 has many side-effects, such as depletion of cellular resources, off-target effects, and increased risk of the emergence of CRISPR/Cas9-resistant escape mutant viruses^41^. To overcome these shortcomings, we engineered autonomous, intelligent, virus-inducible immune-like sense-and-deletion (ALICE_Cas9_) cells. This rewired STING-TBK1-IRF3 pathway and P_ALICE6_ promoter generates “immune-like cells” that can autonomously respond to HSV-1 and switch on the expression of Cas9 only upon the detection of viral dsDNA (Fig. 2a).

To generate ALICE_Cas9_ cells, we replaced the SEAP reporter in ALICE_sen_ with Cas9. The HSV-1-inducible expression of Cas9 was monitored by immunoblotting and a strong correlation was observed between Cas9 expression and HSV-1 multiplicity of infection (MOI) (Supplementary Fig. 2a). Our first iteration of ALICE_Cas9_ immune-like cells was used for targeted deletion of the host cell gene C–C chemokine receptor type 5 gene (*CCR5*). In this iteration, we constitutively expressed an sgRNA targeting *CCR5* (sgRNA_*CCR5*_) (Supplementary Fig. 2b). In the presence of HSV-1, we achieved virus-induced, targeted deletion of the *CCR5* locus of the human genome. We also successfully deleted an EYFP-fusion variant of an exogenous gene (*d2EYFP*) with sgRNA_*d2EYFP*_ (Supplementary Fig. 2c).

After successfully demonstrating the HSV-1-inducible deletion of endogenous and exogenous genes using ALICE_Cas9_ cells, we further demonstrated the self-sense-and-deletion of ALICE_Cas9_ cells in the presence of HSV-1. Envelope glycoprotein E (*US8*), single-stranded DNA-binding protein (*UL29*), and helicase-primase primase subunit (*UL52*) have been demonstrated as functionally essential for viral propagation^25^, therefore sgRNAs targeting these genes were selected to decrease viral replication.

To assess the HSV-1 life-cycle^42,43^, thymidine kinase (*UL23*), DNA polymerase catalytic subunit *(UL30),* and virion protein US2 *(US2)* genes representing immediate early, early and late stages of HSV-1 replication, respectively, were monitored (Fig. 2b–d). ALICE_Cas9_ cells demonstrated antiviral activity in the presence of EGFP-labeled HSV-1 that encodes EGFP reporter gene, as measured by HSV-1 viral RNA levels (Fig. 2b–d), virus infected cells (Fig. 2e), and live virus particles (Fig. 2f and Supplementary Fig. 2d). ALICE_Cas9_ cells showed comparable antiviral effects, compared with the constitutive Cas9 system (Fig. 2b–f and Supplementary Fig. 2d). Since CRISPR relies on base-pairing of sgRNAs to the target nucleotide sequences^44^, multiple sgRNAs targeting different viruses can be incorporated in ALICE_Cas9_. ALICE_Cas9_ cells transfected with tandem sgRNAs targeting both *E1A* gene of ADV and *UL52* gene of HSV-1, demonstrated antiviral activity (ADV and HSV-1) as measured by ADV and HSV-1 RNA levels (Fig. 2g). These data indicating that the ALICE_Cas9_ system can be designed to target and destroy multiple viruses by expressing tandem sgRNAs.

### Design and validation of autonomous, intelligent, virus-inducible immune-like cells with a neutralizing antibody (ALICE_Ab_)

Next, we assessed an ALICE antibody system. We used a known HSV-1 human monoclonal neutralizing antibody E317 (mAb E317Ab)^45^ in HSV-1-inducible immune-like designer (ALICE_Ab_) cells. Specifically, ALICE_Ab_ cells express P_hCMV_-driven STING-PEST (pYW274) and P_ALICE6_-driven His-tag E317Ab (pYW364) (Supplementary Fig. 3a). After confirming the functional role of E317Ab in HSV-1 neutralization, (Supplementary Fig. 3b), we explored the antiviral kinetics of ALICE_Ab_ cells against HSV-1 (Supplementary Fig. 3c). ALICE_Ab_ cells decreased viral load after infection with EGFP-labeled HSV-1 at an MOI of 1~5 (Supplementary Fig. 3c).

As reported by previous study^46^, a cocktail of antibodies REGN10989 and REGN10987 can neutralize SARS-CoV-2^47^. We generated a SARS-CoV-2-responsive ALICE_Ab_ system, containing a P_hCMV_-driven STING protein (pYW274) and a plasmid to drive the antibody production (pYW406, P_ALICE6_-driven REGN10989, and REGN10987) (Supplementary Fig. 4a–c). We found that ALICE_Ab_ was able to reduce SARS-CoV-2 infection by ~70.3 ± 4.3% (Supplementary Fig. 4c).

### Design and validation of autonomous, intelligent, virus-inducible immune-like sense-and-destroy cells (ALICE_Cas9+Ab_)

**Fig. 3:**
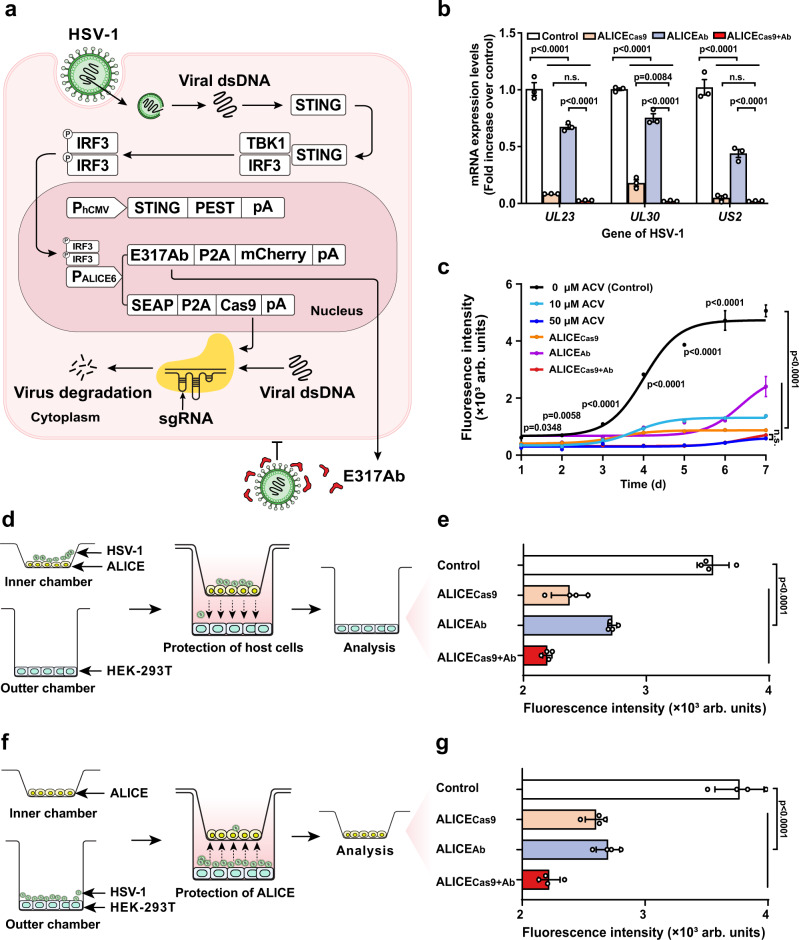
Viral sense-and-destroy function in ALICE system. **a** Schematic illustration of the design principle of ALICE_Cas9+Ab_ system to autonomously sense-and-destroy of HSV-1. HSV-1 activates the ALICE_Cas9+Ab_ system and ultimately initiates the expression of Cas9 and E317Ab under the control of the virus-inducible promotor (P_ALICE6_). HSV-1-inducible Cas9 protein inhibits viral replication by deleting highly conserved sites on virus replication-related genes, while the expression of a recombinant monoclonal antibody that targets an epitope on glycoprotein D of HSV-1 (E317Ab) blocks viral infection. **b** qPCR analysis of EGFP-labeled HSV-1 mRNA levels in the ALICE system. White bars (Control), brown bars (ALICE_Cas9_), blue-gray bars (ALICE_Ab_), red bars (ALICE_Cas9+Ab_) in (**b**, **e**, **g**). *P* values for all other groups versus Control group in the same gene. **c** The comparison study of antiviral effects between acyclovir and ALICE system in mammalian cells. *P* values for ALICE_Cas9+Ab_ group versus Control group, and 50 μM ACV group on the same day. **d** Schematic for the protection function of ALICE system to host cells. Immune-like designer cells containing ALICE system were infected with EGFP-labeled HSV-1 (MOI = 1) for 3 h and then seeded on the inner chamber membrane of a Transwell® apparatus. Before this, HEK-293T cells had been seeded on the bottom of the outer chamber. EGFP expression of HEK-293T cells seeded on the bottom was profiled (**e**). **f** Schematic for the protection function of ALICE system to itself. HEK-293T cells were seeded on the bottom of the outer chamber and infected with EGFP-labeled HSV-1 (MOI = 1) for 3 h. Immune-like designer cells containing ALICE system were then seeded on the inner chamber membrane and embedded into the outer chamber. EGFP expression of immune-like designer cells containing ALICE system seeded on the inner chamber were profiled (**g**). The data in **b**, **c**, **e**, and **g** are expressed as means ± SD; *n* = 3 or 4 independent experiments in **b**, **e**, and **g**; *n* = 3 independent experiments in (**c**); *P* values were calculated by two-way ANOVA with Bonferroni’s post hoc test; n.s. not significant. Source data are provided as a Source Data file.

Following proof-of-concept studies with antiviral ALICE_Cas9_ and ALICE_Ab_ cells, we extended our work by establishing a more robust self-sensing and inhibition system. We constructed ALICE_Cas9+Ab_ cells harboring Cas9 and E317Ab for synergistic activity (Fig. 3a). After generating and confirming the function of ALICE_Cas9_-transgenic stable cell lines (HEK_ALICE-SEAP-Cas9_) (Supplementary Fig. 5) and ALICE_Cas9+Ab_-transgenic stable cell lines (HEK_ALICE-Cas9-E317Ab_) (Supplementary Fig. 6), quantitative profiling and immunoblotting confirmed that the presence of HSV-1 strongly induced the expression of Cas9 and mAb E317Ab in the HEK_ALICE-Cas9-E317Ab_ cells.

We compared the antiviral performance of single-output immune-like designer cells (ALICE_Cas9_ and ALICE_Ab_ cells) against the dual-output immune-like designer ALICE_Cas9+Ab_ cells. ALICE_Cas9+Ab_ system outperformed ALICE_Cas9_ and ALICE_Ab_ system, exhibiting potent and synergistic inhibition of viral replication in HEK-293T cells (Fig. 3b). Subsequent quantitation of SEAP production demonstrated that the modules of ALICE system do not have negative effects either on overall gene expression capacity of the transfected cells (Supplementary Fig. 7). Assessment of the long-term antiviral effects of ALICE_Cas9+Ab_ cells revealed continuous expression of both the Cas9 and E317Ab proteins in the presence HSV-1 over the course of one week (Fig. 3c). We also tested the performance of ALICE_Cas9+Ab_ cells against a known HSV-1 antiviral drug ACV. We found that ALICE_Cas9+Ab_ cells exerted similarly potent antiviral effects against viral replication in HEK-293T cells as a high-dose ACV (50 μM) and found that ALICE_Cas9+Ab_ cells significantly outperformed low-dose ACV (10 μM). The antiviral outputs from ALICE_Cas9+Ab_ cells are specifically induced by the presence of the virus and comprise two orthogonal modes of action against HSV-1 (Fig. 3c). Development of drug resistance by viruses is a major cause for concern^48^ and our findings clearly highlight the attractive potential of multi-output therapeutics based on pathogen-responsive cells.

Furthermore, we performed two types of Transwell®-based assays to characterize antiviral performance of ALICE_Cas9+Ab_ cells: protection against viral spread among host cells (HEK-293T cells) and protection against infection of the designer cells (ALICE_Cas9+Ab_) themselves. The presence of functioning ALICE_Cas9+Ab_ cells strongly inhibited the spread of HSV-1 among HEK-293T cells (Fig. 3d, e). Infected HEK-293T cells were added to functioning ALICE_Cas9+Ab_ or ALICE-like cells and the signal for EGFP-labeled HSV-1 was dramatically lower in the functional ALICE_Cas9+Ab_ cells at 48 h post-infection (hpi) (Fig. 3f, g), confirming the self-protection capacity of the ALICE_Cas9+Ab_ cells.

### Sense-and-destroy against HSV-1 mediated by ALICE in mice

**Fig. 4:**
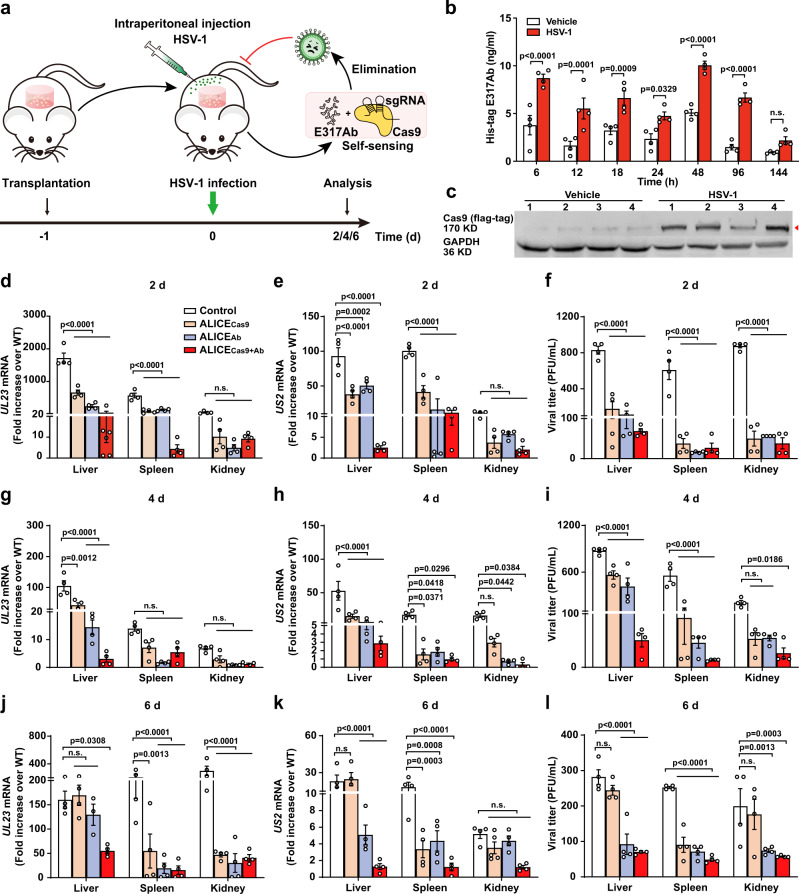
Sense-and-destroy against HSV-1 mediated by ALICE in mice. **a** Schematic illustration of single- and dual-output ALICE systems for autonomous sense-and-destroy against HSV-1 in mice. Four types of cells, including (1) three sgRNAs (pYW102/pYW172/pYW188, respectively targeting *US8*/*UL29*/*UL52*)-transgenic HEK_ALICE-SEAP-Cas9_ (ALICE_Cas9_), (2) pcDNA3.1-transgenic HEK_ALICE-Cas9-E317Ab_ (ALICE_Ab_), (3) pYW102/pYW172/pYW188-transgenic HEK_ALICE-Cas9-E317Ab_ (ALICE_Cas9+Ab_), or (4) pcDNA3.1-transgenic HEK-293T cells (Control), were encapsulated into hydrogel-based scaffolds and transplanted into the abdomen of mice via intraperitoneal surgery. At 20 h post-transplantation of the hydrogel implants, HSV-1 (2 × 10^7^ PFU) was intraperitoneally injected into each mouse. The antiviral effects of the single- and dual-output ALICE systems in mice were evaluated by detecting residual viral titers in indicated organs (liver, spleen, kidney) at 2, 4 and 6-days post HSV-1 injection (**d**–**l**). **b** Assay of HSV-1-inducible E317Ab expression and **c** western blot analysis of HSV-1-inducible Cas9 expression in hydrogel-scaffolds. Mice were transplanted with hydrogel-scaffolds containing pYW102/pYW172/pYW188-transgenic HEK_ALICE-Cas9-E317Ab_ cells, infected with HSV-1 (2 × 10^7^ PFU, HSV-1 group) or uninfected (0, Vehicle group). The red arrowhead indicates the expected Cas9 band. **d** qPCR assay of HSV-1 *UL23*/*US2* mRNA in mice. HSV-1 mRNA levels were performed in isolated liver/spleen/kidney using specific *UL23*/*US2* primers listed in Supplementary Table 2 at 2 (**d**, **e**), 4 (**g**, **h**), and 6 (**j**, **k**) days post HSV-1 injection. White bars (Control), brown bars (ALICE_Cas9_), blue-gray bars (ALICE_Ab_), red bars (ALICE_Cas9+Ab_) in (**d**–**l**). **f** Viral titers in mice. The mice were processed as described in (**a**). Virus in isolated tissues, such as liver, spleen, kidney, were titrated at 2 (**f**), 4 (**i**), and 6 (**l**) days post HSV-1 injection. The relative expression was calculated using the ΔΔct method based on the expression levels of viral genes *UL23/US2* in organs of wild-type mice (WT). *P* values for all other group versus Control group in the same tissue. Data in **d**, **e**, **g**, **h**, **j**, **k** are normalized to wild-type mice (WT); Numbers 1–4 represents four indenpent mice in (**c**); Data in **b**, **d**–**l** are expressed as means ± SEM; *n* = 4 mice; *P* values were calculated by two-way ANOVA with Bonferroni’s post hoc test; n.s. not significant. Source data are provided as a Source Data file.

We performed a pilot study to rigorously assess the feasibility of ALICE systems in vivo, by establishing a series of HSV-1-infected mouse models. To examine the potential of ALICE systems to prevent viral infections, we delivered immune-like cells containing our single- and dual-output ALICE systems into mice and evaluated the antiviral effects (Fig. 4a). From a biocompatibility perspective, we selected hyaluronic acid-based hydrogels, as graft materials for immune-like cells, that are made of hydrophilic polymer chains, cross-linked to swell and retain their three-dimensional structure without dissolving^49^. We embedded control HEK-293T cells, both types of the single-output ALICE_Ab_/ALICE_Cas9_ cells, and the dual-output ALICE_Cas9+Ab_ cells, into hydrogel scaffolds and introduced the matrix into the abdomen of mice via intraperitoneal transplantation. Subsequently, we challenged mice with HSV-1 (2 × 10^7^ PFU, plaque-forming unit) at 20 h post-transplantation. The mice were sacrificed at day 2, 4, and 6 post-infection (dpi) to monitor viral titers in organs including liver, spleen, and kidney (Fig. 4a). We determined that ALICE_Cas9+Ab_ had the best antiviral effect for up to 6 days, then further examined the antiviral activity in this system. E317Ab expression in ALICE_Cas9+Ab_ cells-implanted mice was significantly higher when challenged with virus (Fig. 4b). Likewise, we excised the hydrogels and confirmed that Cas9 expression in mice bearing scaffolds with ALICE_Cas9+Ab_ cells was much higher when challenged with HSV-1, compared with the non-challenge group (Fig. 4c). As shown in Supplementary Fig. 8, no significant difference was found among three groups, including non-treated mice (WT), unmodified-hydrogel-implanted mice (Control), and designer-hydrogel-implanted mice (ALICE_Cas9+Ab_), indicating that hydrogel-based transplantation alone did not invoke host-mediated, foreign-body responses.

The organs of mice that received the control HEK-293T cells had high levels of viral RNAs including *UL23*/*US2* at the various dpi. In contrast, lower level of viral RNAs was detected in organs of the animals given the dual-output ALICE system. The viral RNA levels in organs were at intermediate levels upon transplantation with the single-output ALICE systems, with the mAb system slightly outperforming the Cas9 system (Fig. 4d–l). The superior performance of the dual-output ALICE system was increasingly evident over time (Fig. 4d–l), and the most pronounced effect was detected in the liver at 6 days post-transplantation (Fig. 4j–l).

### Long-term sense-and-destroy against HSV-1 mediated by ALICE_Cas9+sgRNAs+Ab_ in mice

**Fig. 5:**
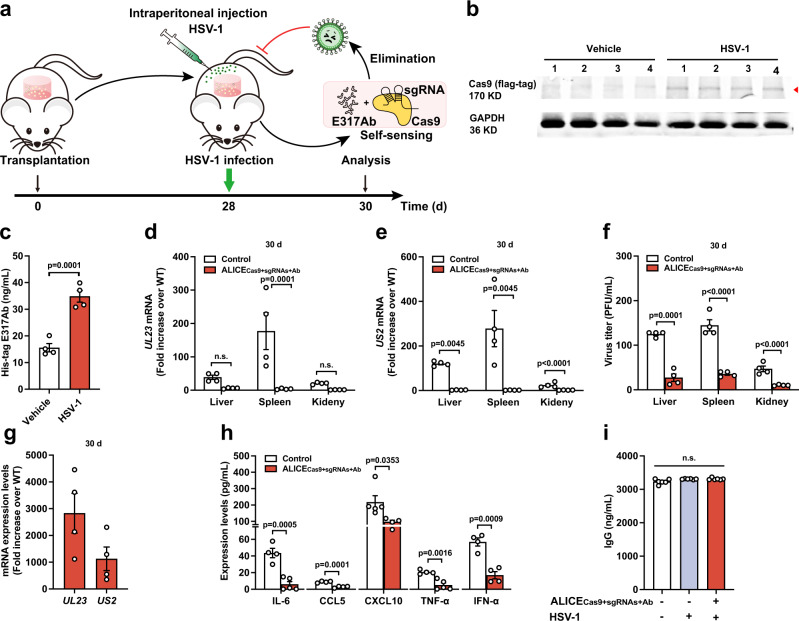
Long-term sense-and-destroy against HSV-1 mediated by ALICE_Cas9+sgRNAs+Ab_ in mice. **a** Schematic illustration of ALICE_Cas9+sgRNAs+Ab_ system for autonomous sense-and-destroy against HSV-1 in mice. HEK-293T cells stably integrated with ALICE_Cas9+sgRNAs+Ab_ (HEK_ALICE-Cas9-sgRNAs-E317Ab_ cells) or pcDNA3.1-transgenic HEK-293T cells (Control) were encapsulated into hydrogel-based scaffolds and transplanted into mice abdomens via intraperitoneal surgery. At 28 d post-transplantation, HSV-1 (2 × 10^7^ PFU) was intraperitoneally injected into each mouse. The antiviral effects of the ALICE_Cas9+sgRNAs+Ab_ in mice was evaluated by detecting residual viral titers in the indicated organs (liver, spleen, kidney) at 2 days post HSV-1 infection. **b** Western blot analysis of HSV-1-inducible Cas9 expression in hydrogel-based scaffolds. The red arrowhead indicates the expected Cas9 band. Number 1–4 represents four independent samples. **c** Validation of HSV-1-inducible E317Ab expression in mice. The E317Ab level in the bloodstream from ALICE_Cas9+sgRNAs+Ab_-treated mice, infected with or without HSV-1, was quantified at 30 days post-transplantation. **d**, **e** qPCR analysis of HSV-1 *UL23*/*US2* mRNA levels in mice. **f** Viral titers in mice. Virus in isolated tissues (liver, spleen, kidney) from ALICE_Cas9+sgRNAs+Ab_-treated mice, infected with or without HSV-1, were titrated at 30 days post-transplantation. **g** qPCR analysis of HSV-1 *UL23/US2* mRNA in hydrogel implants at 30 days post-transplantation. **h** Cytokine levels in mice. Cytokines (IL-6, CCL5, CXCL10, TNF-α, and IFN-α) in the blood from HSV-1-infected mice implanted with or without ALICE_Cas9+sgRNAs+Ab_ were analyzed by flow cytometry at 30 days post-transplantation. **i** IgG expression in mice. Mice implanted with or without ALICE_Cas9+sgRNAs+Ab_ were infected with or without HSV-1. IgG levels in the bloods were analyzed using an IgG ELISA at 30 days post-transplantation. Data in **d**, **e**, and **g** are normalized to wild-type mice (WT); Data in **c**–**i** are expressed as means ± SEM; *P* values in **c**, **h** were calculated by two-tailed unpaired *t*-test; *P* values in **d**–**f** were calculated by two-way ANOVA with Bonferroni’s post hoc test; *P* values in **i** were calculated by one-way ANOVA followed by a Dunnett’s post hoc test; *n* = 4 mice in **c**–**g**, *n* = 4 or 5 mice in (**h**), *n* = 6 mice in (**i**). Source data are provided as a Source Data file.

We subsequently evaluated the long-term antiviral functionality of ALICE_Cas9+Ab_ cells in mice. The hydrogel implantation of the stable dual-output HEK_ALICE-Cas9-sgRNAs-E317Ab_ cells (ALICE_Cas9+sgRNAs+Ab_ cells) into mice was the same as described in Fig. 4a (Fig. 5a). To determine the longevity of ALICE_Cas9+sgRNAs+Ab_ cells activity in vivo, we challenged the hydrogel-implanted mice with HSV-1 (2 × 10^7^ PFU) at 28 days post-transplantation. We sacrificed mice at 30 days post-transplantation to assess viral titers in organs including liver, spleen, and kidney (Fig. 5a). The location and shape of hydrogels was recorded before and after transplantation (Supplementary Fig. 9). Our observations were consistent with previously published studies where the viability of cells in hydrogels are maintained for at least 30 days^50^. Cas9 expression in ALICE_Cas9+sgRNAs+Ab_ cells-implanted mice was much higher when challenged with HSV-1, compared with the non-challenged group (Fig. 5b) and similar findings were observed for the E317Ab expression (Fig. 5c).

We then monitored HSV-1 mRNA levels at 30 days post-transplantation in mouse organs positioned near the transplant sites (liver, spleen, and kidney) (Fig. 5d, e). Our results showed that the organs derived from unmodified-HEK-293T cells-implanted mice had higher levels of viral RNAs and titers, compared with ALICE_Cas9+sgRNAs+Ab_ cells-implanted mice (Fig. 5d–f). To determine shuttle efficacy of HSV-1 in hydrogels, we used quantitative PCR with reverse transcription (RT-qPCR) to quantify copies of HSV-1 mRNA in unmodified-HEK-293T-hydrogels challenged with HSV-1 (Control), normalized to hydrogels without HSV-1. The average level of HSV-1 mRNA (*UL23* and *US2*) in unmodified-HEK-293T-hydrogels challenged with HSV-1 was 1000–3000 folds higher than hydrogels without HSV-1 (Fig. 5g). There is consensus that severe virus infection results in excessive virus-induced inflammation mediated by the infiltration of inflammatory cells, including T cells (both CD4^+^ and CD8^+^), polymorphonuclear leukocytes and macrophages^5^. Indeed, HSV-1 infection provokes the expression of the inflammatory molecules IL-6, CCL5, CXCL10, TNF-α, and IFN-α, which was blocked after ALICE_Cas9+sgRNAs+Ab_ cells-hydrogel transplantation (Fig. 5h). Moreover, the innate immune response of mice is important for antiviral effects. Our results showed that there is no significant difference in the expression of Immunoglobulin G (IgG) between unmodified-HEK-293T-hydrogels implanted mice (Control) and ALICE_Cas9+sgRNAs+Ab_ cells-hydrogels-implanted mice (treated group), when mice were challenged with HSV-1 (Fig. 5i).

### Inhibition of HSV-1 transmission mediated by ALICE_Cas9+Ab_ in mice

During HSV-1 latency, the viral genome is harbored in peripheral neurons in the absence of infectious virus but has the potential to restart infection^51^. To mimic an organ-transplant recipient who received a latent HSV-1-infected organ, we developed a mouse model where mice received HSV-1-infected ALICE_Cas9+Ab_ cells. To verify the inhibition of viral spread efficacy, we evaluated the inhibition of viral transmission mediated by ALICE_Cas9+Ab_, which were infected with HSV-1 before implantation into mice. We used the engineered stable cell line HEK_ALICE-Cas9-E317Ab_ infected with HSV-1 and stabilized the cells in hyaluronic acid-based hydrogel scaffolds. The hydrogel scaffolds were intraperitoneally transplanted into mice as a central point of infection for surrounding organs, thereby establishing infection in the mice (Supplementary Fig. 10a). We first examined the inducible expression of ALICE proteins by excising the hydrogels containing uninfected HEK_ALICE-Cas9-E317Ab_ cells or HSV-1-infected HEK_ALICE-Cas9-E317Ab_ cells at 6 days post-transplantation. Total proteins were extracted from these scaffolds and immunoblotting revealed abundant Cas9 expression in the scaffolds bearing the HSV-1-infected HEK_ALICE-Cas9-E317Ab_ cells but very low background in the control scaffolds. These results highlighted both the virus inducibility of ALICE and a low level of leaky Cas9 expression in the absence of the virus (Supplementary Fig. 10b). Similar observations were obtained for E317Ab expression (Supplementary Fig. 10c). Further, analyses of serum collected at 2-, 4-, and 6-days post-transplantation revealed that the IL-6 levels were consistently higher in the animals transplanted with the HSV-1-infected HEK-293T cells than the animals with the HSV-1-infected HEK_ALICE-Cas9-E317Ab_ cells (Supplementary Fig. 10d), indicating the antiviral effects of ALICE_Cas9+Ab_.

We then monitored the presence of the viral RNA (*UL23* and *US2*) level in mouse organs positioned near the transplant site (liver, spleen, and kidney) (Supplementary Fig. 10e, f, h, i, k, l). The organs of animals transplanted with infected HEK-293T hydrogel scaffolds exhibited significantly higher viral RNA levels and titers than HEK_ALICE-Cas9-E317Ab_ hydrogel scaffolds (Supplementary Fig. 10e–m).

### Sense-and-destroy against HSV-1 mediated by ALICE_Cas9+Ab_ in a virus-infected mouse model

**Fig. 6:**
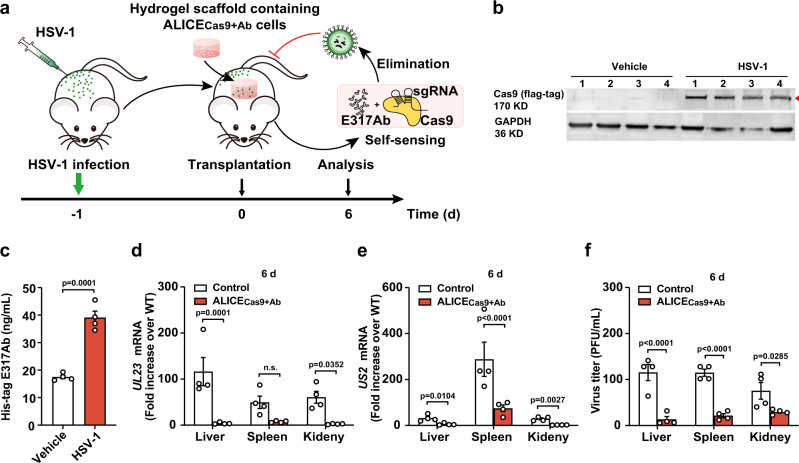
Sense-and-destroy against HSV-1 mediated by ALICE_Cas9+Ab_ in a virus-infected mouse model. **a** Schematic illustration of ALICE_Cas9+Ab_ for autonomous sense-and-destroy against HSV-1 in a virus-infected mouse model. Three sgRNAs (pYW102/pYW172/pYW188, respectively targeting *US8*/*UL29*/*UL52*)-transgenic HEK_ALICE-Cas9-E317Ab_ cells (ALICE_Cas9+Ab_) or unmodified HEK-293T cells (Control) were encapsulated into hydrogel-based scaffolds. At 20 hpi of HSV-1 (2 × 10^7^ PFU) by intraperitoneal injection, mice were implanted with the ALICE_Cas9+Ab_ by intraperitoneal surgery. The antiviral effects of ALICE_Cas9+Ab_ in mice were evaluated by detecting viral titers in indicated mouse organs (liver, spleen, kidney) at 6 days post-transplantation. **b** Western blot analysis of HSV-1-inducible Cas9 expression in hydrogel-based scaffolds and **c** validation of HSV-1-inducible E317Ab expression. HSV-1-infected mice (2 × 10^7^ PFU, HSV-1 group) or uninfected (0, Vehicle group) were all transplanted with hydrogel-based scaffolds containing pYW102/pYW172/pYW188-transgenic HEK_ALICE-Cas9-E317Ab_ cells. Total protein from hydrogel implants isolated from mice were extracted for Western blot analysis at 6 days post-transplantation. The red arrowhead indicates the expected Cas9 band. E317Ab levels in the blood were analyzed by using a His-tag ELISA at 6 days post-transplantation. **d**, **e** qPCR assay of HSV-1 *UL23/US2* mRNA in liver/spleen/kidney at 6 days post-transplantation between Control and ALICE_Cas9+Ab_ group, using primers listed in Supplementary Table 2. The relative expression values were calculated using the ΔΔct method based on the expression levels of the viral genes *UL23/US2* in organs of WT mice without HSV-1 infection and intraperitoneal transplantation of any hydrogel. **f** Viral titers in mice. The mice were processed as described in (**a**). Virus in tissues, such as liver, spleen, kidney, were measured titrated at 6 days post-transplantation. IgG levels in the blood were analyzed by using an IgG ELISA at 6 days post-transplantation. Number 1–4 represents four indenpent mice in b; Data in c-f are expressed as means ± SEM; *P* values in **c** were calculated by two-tailed unpaired *t*-test; *P* values in **d**–**f** were calculated by two-way ANOVA with Bonferroni’s post hoc test; *n* = 4 mice. Source data are provided as a Source Data file.

To examine the antiviral potential of immune-like designer cells ALICE _Cas9+Ab_ in vivo, we used a virus-infected mouse model where mice were first infected with HSV-1 (2 × 10^7^ PFU) via intraperitoneal injection. At 20 hpi, the engineered stable HEK_ALICE-Cas9-E317Ab_ cells were encapsulated into hyaluronic acid-based hydrogel scaffolds, which were intraperitoneally transplanted into mice as a self-sensing antiviral device, thereby decreasing infection in the mice (Fig. 6a). We examined the inducible protein expression from ALICE_Cas9+Ab_ by excising the ALICE_Cas9+Ab_ hydrogels from HSV-1-uninfected or -infected mice at 6 days post-transplantation. Total proteins were extracted from these scaffolds: immunoblotting revealed abundant Cas9 expression in HSV-1-infected mice containing ALICE_Cas9+Ab_ scaffolds, but very low levels in the HSV-1-uninfected mice (Fig. 6b). These results indicated that virus present in the abdominal cavity can freely shuttle into the scaffolds, where it can initiate an antiviral response from the ALICE_Cas9+Ab_ hydrogels and triggered the E317Ab expression in mice containing ALICE_Cas9+Ab_-hydrogel implant (Fig. 6c).

We then determined the levels of HSV-1 viral RNA (*UL23* and *US2*) at 6 days post-transplantation in mouse organs positioned near the transplant sites (liver, spleen, and kidney) (Fig. 6d, e). Our results showed that the organs of unmodified-HEK-293T cells-implanted mice exhibited strong expression of viral RNAs and viral titers, compared with ALICE_Cas9+Ab_ cells-implanted mice, notably where all mice were challenged with HSV-1 (2 × 10^7^ PFU) (Fig. 6d–f).

### Long-term sense-and-destroy against HSV-1 mediated by AAV-ALICE_SaCas9+Ab_ in a herpetic simplex keratitis mouse model

After assessing the antiviral efficacy of the ALICE system in mouse models via cell therapy, we next investigated the antiviral potential of ALICE_Cas9+Ab_ delivered by AAV-vector in a HSK mouse model, which mimics natural HSV-1 infection. As reported by previous studies, AAV-mediated delivery of meganucleases^52^, *Streptococcus pyogenes* Cas9 (SpCas9), or *Staphylococcus aureus* Cas9 (SaCas9)^53^ mediated highly efficient gene editing of HSV-1 from TG. AAV has shown great promise for gene delivery in vivo as well as in TG neurons. Due to the packaging limit of AAV (<4500 bp), SaCas9 (3156 bp) is more suitable for AAV-mediated delivery than SpCas9 (4101 bp), which leaves no space for AAV-ALICE_SaCas9+Ab_ regulatory elements. As an expression of SaCas9 with HSV-1 immediate-early regulatory protein ICP4-targeted sgRNA can lead to the decrease of the fluorescence intensity of EGFP trigged by HSV-1 replication, we confirmed that expression of the CRISPR-SaCas9 system could reduce HSV-1 infection (Supplementary Fig. 11a, b).

**Fig. 7:**
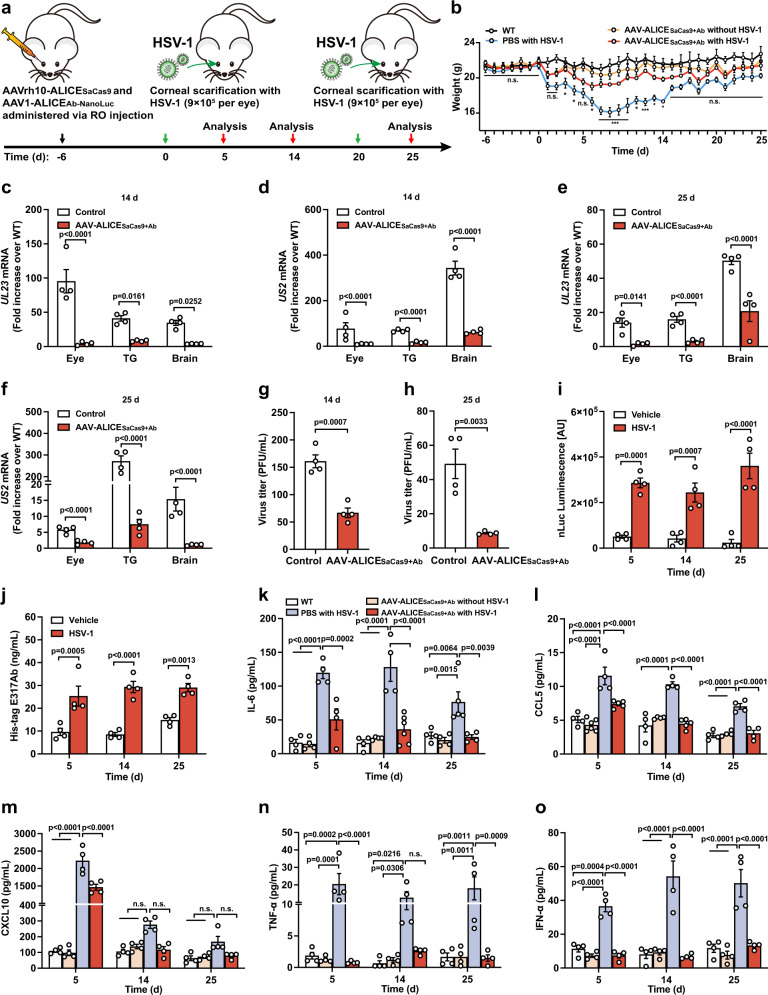
Long-term sense-and-destroy against HSV-1 mediated by AAV-ALICE_SaCas9+Ab_ in a herpetic simplex keratitis mouse model. **a** Schematic illustration of AAV-ALICE_SaCas9+Ab_ for autonomous sense-and-destroy against HSV-1 in a herpetic simplex keratitis mouse model. Two packaged AAV vectors, AAVrh10-ALICE_saCas9_ (5 × 10^11^ PFU) and AAV1-ALICE_Ab_ (5 × 10^11^ PFU), were simultaneously injected into each mouse via RO route 6 days prior to initial HSV-1 infection. Control mice were injected with PBS. At 0 day and 20 days, HSV-1 (9 × 10^5^ PFU) or PBS was delivered by corneal scarification of each eye using a 28-gauge needle. The antiviral effects of ALICE-packaged AAVs in mice was evaluated by measuring residual viral titers in the indicated mouse organs (eye, TG, brain) at 14- and 25-days post initial HSV-1 infection. **b** Change in body weight. *P* values are provided in the Source data file. **c** qPCR analysis of HSV-1 *UL23*/*US2* mRNA in mice at 14 days (**c**, **d**) and 25 days (**e**, **f**) post initial HSV-1 infection. Viral titers in mice. Virus in the brain was titrated at 14 days (**g**) and 25 days (**h**) post initial HSV-1 infection. **i** Assay of HSV-1-induced nanoLuc expression in mice. NanoLuc levels from AAV-ALICE_SaCas9+Ab_-treated mice infected with or without HSV-1, were analyzed at day 5, 14 and 25 post initial HSV-1 infection. **j** Assay of HSV-1-inducible E317Ab expression in mice. Assay of cytokine expression in mice. Cytokine levels, IL-6 (**k**), CCL5 (**l**), TNF-α (**m**), IFN-α (**n**), and CXCL10 (**o**) in the blood were analyzed by flow cytometry at day 5, 14, and 25 post initial HSV-1 infection. White bars (WT), brown bars (AAV-ALICE_SaCas9+Ab_ without HSV-1), blue-gray bars (PBS with HSV-1), red bars (AAV-ALICE_SaCas9+Ab_ with HSV-1) in (**k**–**o**). Data in **c**–**f** are normalized to wild-type mice (WT); Data in **b**–**o** are expressed as means ± SEM; *P* values in **c**–**f**, **i**–**o** were calculated by two-way ANOVA with Bonferroni’s post hoc test; *P* values in **g**, **h** were calculated by two-tailed unpaired *t*-test; *n* = 4–9 mice in (**b**), *n* = 4 mice in (**c**–**j**), *n* = 4–6 mice in (**k**–**o**). n.s. not significant. Source data are provided as a Source Data file.

To test whether the AAV-ALICE_SaCas9+Ab_ system can eliminate HSV-1 in TG, we established an HSK mouse model. The AAV-ALICE_SaCas9+Ab_ system consists of two AAV elements: firstly, the AAVrh10-ALICE_SaCas9_ carries an HSV-1-induced SaCas9 and a constitutive P_U6_-driven expression of HSV-1-targeted *ICP4* sgRNA (5 × 10^11^ PFU/mouse); secondly, the AAV1-ALICE_Ab_ carries an HSV-1-induced E317Ab-P2A-nanoLuc and a constitutive P_hCMV_-driven expression of STING-PEST (5 × 10^11^ PFU/mouse). Wild-type mice were injected with AAV-ALICE_SaCas9+Ab_ system via retro-orbital (RO) injection. At 6 days post AAV-ALICE_SaCas9+Ab_ system injection, challenge with HSV-1 infection (9 × 10^5^ PFU per eye) was conducted following corneal scarification at 0 and 20 days to mimic the HSK mice (Fig. 7a). Body weight was recorded daily. Significant weight loss was observed for the HSV-1-treated mice without the AAV-ALICE_SaCas9+Ab_ system, and a clear difference was observed between the HSV-1 infected mice in the presence or absence of the AAV-ALICE_SaCas9+Ab_ system (Fig. 7b). Next, we evaluated the virus titers in isolated corneas, TG and brain, at 14- and 25-days post initial HSV-1 infection, and found significantly lower titers in all tested organs isolated from AAV-ALICE_SaCas9+Ab_ system-treated mice than PBS-treated mice (Fig. 7c–h).

Finally, we examined whether RO injection of AAV-ALICE_SaCas9+Ab_ system induces HSV-1-responsive nanoLuc (Fig. 7i) and E317Ab (Fig. 7j) in the bloodstream. We observed significantly higher HSV-1-responsive nanoLuc and E317Ab in the AAV-ALICE_SaCas9+Ab_ system-treated mice challenged with HSV-1 (9 × 10^5^ PFU per eye) than those without HSV-1. Moreover, HSV-1 infection induced expression of the inflammatory molecules IL-6, CCL5, CXCL10, TNF-α, and IFN-α, which was blocked after AAV-ALICE_SaCas9+Ab_ system treatment at 5-, 14-, and 25-days post first HSV-1 infection (Fig. 7k–o). Notably, our results showed that there is no significant difference in the expression of IgG between PBS-treated mice (Control) and AAV-ALICE_SaCas9+Ab_ system-treated mice (Treated group), where all mice were challenged with HSV-1 (*n* = 4 mice; non-significant, two-tailed Student’s *t* tests) (Supplementary Fig. 11c). Taken together, these results suggest that the administration of AAV-ALICE_SaCas9+Ab_ system significantly reduced the manifestation of disease severity in the HSK mouse model.

## Discussion

Here, we showed that the immune-like designer cells (ALICE cells) harboring a STING-mediated genetic circuit can effectively detect multiple viruses and we demonstrated the utility of ALICE systems for detection and inhibition/elimination of viruses based on nuclease-mediated cleavage and antibody-mediated virus neutralization. The ALICE system can detect and mitigate infections before symptoms appear, which is particularly beneficial for immune compromised patients for whom infections can be life-threatening^54^.

The availability of a closed-loop gene network to regulate therapeutic agent release in mammalian cells could facilitate implementation of novel anti-infection therapies. Protein therapeutics like our examples of antiviral cytokines (human IFN-α or IFN-β)^38^, Cas9^55^, and the neutralizing antibody E317Ab^56^ represent promising alternatives to conventional antiviral drugs^57,58^. As engineered cells can accommodate many modifications to their genomes, entire biosynthetic pathways for the release of potent antiviral molecules could be engineered into these immune-like designer cells. Therefore, the ALICE system can deploy alternative therapies that are collectively less likely to induce the development of antiviral resistance. Beyond demonstrating a clinically relevant level of anti-HSV-1 potency, our results illustrate a potential to overcome some of the known problems with the emergence of ACV-resistant virus strains (Fig. 3c)^59^.

The ALICE_Cas9_ system could be modified to achieve high viral cleavage efficiency based on alternative sgRNAs that target other viral regions or even target distinct sites of multiple viruses. In addition, the virus-specific neutralizing antibody output modules of ALICE_Ab_ can be changed to express the best-available antibodies to increase potency and/or therapeutic half-life or target various viruses, such as SARS-CoV-2 (Supplementary Fig. 4). These modifications can be combined for new outputs from ALICE_Cas9+Ab_ for combination therapies to achieve synergistic antiviral effects.

There is no cure available for HSV-1 and once a person is infected, the virus remains dormant in the TG, with the potential to reactivate. The leading treatment for HSV-1 infection is acyclovir, a purine nucleoside analog that can inhibit viral replication. These antiviral treatment options are given in combination with topical steroids, which may not be suitable for long-term use due to their side effects, such as secondary glaucoma, infection, and cataracts. This is a major limitation when it comes to treating HSK, which is often recurrent and requires extended and as well prophylactic treatment regimens^60^. Gene therapy became possible through the advances of genetics and bioengineering that enabled manipulating vectors for delivery of extrachromosomal material to target cells^61^. Therefore, we use the engineered AAV vectors to deliver all DNA elements of our ALICE_SaCas9+Ab_ into targeted tissues for a long-term, cocktail HSK therapy.

The success of ALICE cells to control HSV-1 infection in mice, delivered at different stages of virus infection either before, during, or after the transplantation of hydrogel scaffolds, via cell therapy or into HSK mice via gene therapy, warrants further investigation to explore ALICE’s utility against other viruses^62^.

It is important to note that the current study was designed to deliver a specific output in response to HSV-1 infection; the ALICE system could in theory, detect any dsDNA virus infection. Moreover, considering that many highly pathogenic viruses are RNA viruses (SARS-CoV-2, Nipah virus, Ebola virus, and influenza virus), application of the ALICE concept should be explored for the development of surveillance and treatment systems against RNA viruses. This could be achieved by replacing the STING-based sensor of our current ALICE system with an RNA sensor such as the retinoic-acid-inducible gene I (RIG-I). Successful activation of our current STING-based ALICE sensor by SARS-CoV-2 and DENV-2 could have resulted from the release of nuclear or mitochondrial DNA due to cellular stress or cytokine response induced by infection^29,63^.

Additional opportunities for the further development and medical translation of immune-like designer cell-based therapies like ALICE include clinical manipulation of patient-derived cells, non-invasive monitoring of cell stability, and the potential for long-duration performance stretching over many months or longer^64^. These challenges do not seem insurmountable. One technical aspect that should promote such efforts would be the modification of implantation materials, which previous efforts have shown to increase the safety and efficacy of cellular transplants and thereby facilitate the maintenance of the engineered cellular functions for extended periods of time in vivo^65^.

Collectively, the modular design of ALICE systems, comprising a selected host cell chassis, a pathogen-detection sensor module, a rewired endogenous signaling pathway, diversely regulated output pathways, and delivered by designer cells or AAV-vectors, should flexibly accommodate the specific application requirements (suitable antigen, suitable control agents) for many infectious diseases. We anticipate ALICE_im_ can function as an artificial innate immune system, protecting the host organism through nonspecific immune defense by the induction of interferons. We foresee that the diverse examples of ALICE systems we have demonstrated in the present study can serve as illustrative models, which can be readily adapted for the development of immune-like designer cells to achieve sense-and-destroy functions for a potentially very large number of pathogens that target mammals.

## Methods

### Ethical statement

All the experiments involving mice were performed according to the approved protocols by the East China Normal University (ECNU) Animal Care and Use Committee (protocol ID: m20180403). All the procedures for sample or data collection used were carried out in compliance with the Ministry of Science and Technology of the People’s Republic of China on Animal Care Guidelines. All mice were euthanized after the experiments.

### Cloning and vector construction

Design and construction details of each plasmid are provided in Supplementary Table 1. All genetic components were confirmed by DNA sequencing (Genewiz Inc.).

### Cell culture

Human cervical adenocarcinoma cells (HeLa, ATCC: CCL-2), HEK-293T-derived Hana3A cells engineered for the stable expression of G_αολΦ_ and chaperones RTP1/RTP2/REEP1, HEK-293T-derived HEK-293A cells containing a stably integrated copy of the E1 gene (ThermoFisher, cat. no. R70507), telomerase-immortalized human mesenchymal stem cells (hMSC-TERT, ATCC: SCRC4000), human embryonic kidney cells (HEK-293T, ATCC: CRL-11268), African green monkey kidney epithelium-derived Vero cells (Vero, ATCC: CCL-81), Vero E6 cells (ATCC: CRL-1586) rhadomyosarcoma (RD, ATCC: CCL-136), Huh7.5.1 cells and Huh7-NTCP cells were cultivated in DMEM (Gibco, cat. no. 31600-083) supplemented with 10% (v/v) fetal bovine serum (FBS, Biological Industries, cat. no. 04-001-1C) and 1% (v/v) penicillin/streptomycin solution (Sangon Biotech, cat. no. B540732-0010). Aedes albopictus cells (C6/36, ATCC: CRL-1660) were incubated at 28 °C, and all other cell types were incubated at 37 °C in a humidified atmosphere incubator, containing 5% CO_2_ and were regularly tested for the absence of mycoplasma and bacterial contamination.

### Virus preparation

HSV-1 and replication-competent HSV-1 (strain 17) with non-necessary gene (*UL2*) replaced by enhanced green fluorescent protein gene (EGFP-labeled HSV-1) were gifted by Professor Ping Wang (Tongji University) and Professor Erguang Li (Nanjing University), respectively. HSV-1 was propagated in Vero cells. H1N1, Pteropine orthoreovirus PRV2P, hCoV-229E and SARS-CoV-2 isolate BetaCoV/Singapore/2/2020 (GISAID accession no. EPI_ISL_406973) were propagated in Vero E6 cells. EV-A71 isolate 5865/SIN/000009 (GenBank accession no. AF316321) was produced using HeLa cells. VSV was gifted by Professor Peng Zhou (Wuhan Institute of Virology, Chinese Academy of Sciences). Lentivirus, AAV and Adenovirus 5 (ADV) were purchased from ObiO Technology (Shanghai) Corp., Ltd. DENV-2 strain was propagated in *Aedes albopictus* cells at 28 °C.

### Virus titration

HSV-1 infectivity was evaluated using 50% tissue culture infective dose assays (TCID_50_). Briefly, cells seeded in 96-well plates were infected with serially diluted virus, eight replicates per dilution. For each dilution, the number of wells that were positive for cytopathic effect (CPE) was scored. A Reed and Muench calculation was then performed to determine the 50% infectious dose^66^. And CPE was assessed at 4–7 dpi. HSV-1/EGFP-labeled HSV-1 was titrated by plaque assay in Vero cells. H1N1, PRV2P, hCoV-229E and SARS-CoV-2 were titrated by plaque assay in Vero E6 cells. EV-A71 was titrated on RD cells. The quantification of viruses (SARS-CoV-2, HCV, HBV, ADV, HSV-1) was performed by qPCR using the primers target corresponding genes [receptor binding domain for SARS-CoV-2 Spike protein (*RBD)*, HCV genomic RNA, HBV pre-genomic RNA (pgRNA), *E1B*/*E2 early*, *UL23*/*UL30*/*US2*] and QuantiFast SYBR Green RT-PCR kit (Qiagen) following the manufacturer’s instructions. All primers are listed in Supplementary Table 2.

### Cell transfection and virus infection

All cell lines, except Huh7.5.1 and Huh7-NTCP cells, were transfected with an optimized polyethyleneimine (PolyScience)-based protocol. Huh7.5.1 cells/Huh7-NTCP cells were transfected using lipofectamine 2000 (Invitrogen) according to the manufacturer’s instructions.

Transgenic cells were infected with different viruses (H1N1, EV-A71, PRV2P, VSV, Lentivirus, AAV, DENV-2, SARS-CoV-2, hCoV-229E, ADV, and HSV-1) for 1 h, and transgenic-Huh7.5.1 cells/Huh7-NTCP cells were incubated with HCV/HBV for 24 h. SEAP production in culture supernatants was quantified at indicated time points by SEAP reporter assay as previously reported^67^. SEAP expression levels in the cell culture supernatant were quantified using a Synergy H1 hybrid multi-mode microplate reader with Gen5 software (version: 2.04).

### Sense-and-clearance against multiple viruses mediated by ALICE_im_ in mammalian cells

Virus-inducible cytokines production in ALICE_im_. pYW274/pYW365-transgenic cells were incubated with HCV (MOI = 3), HBV [1000 virion genome equivalents (vge)/cell], ADV (MOI = 10), HSV-1 (MOI = 5) and Vehicle (equal volume of DMEM) as control for 24 h. The IFN-α/IFN-β production in culture supernatants was quantified by ELISA at 2/4 dpi, using a human IFN-α ELISA kit (Beyotime, cat. no. PI505) or human IFN-β ELISA kit (Beyotime, cat. no. PI572) according to manufacturer’s instructions.

qPCR analysis of the viral transcript genes in ALICE_im_. pYW274/pWS67-transgenic cells (ALICE_sen_-SEAP), pYW274/pYW365-transgenic cells (ALICE_im_-IFNα) or pYW274/pYW327-transgenic cells (ALICE_im_-IFNβ) were incubated with the HCV (MOI = 3), HBV (1000 vge/cell), ADV (MOI = 10), HSV-1 (MOI = 5) for 24 h. The relative viral mRNA expression was quantified by qPCR at 2/4 dpi. All data were normalized to the viral gene expression levels in ALICE_sen_-SEAP control group infected with the corresponding viruses. All primers used are provided in Supplementary Table 2.

### HSV-1 infection and inhibition assay

EGFP-labeled HSV-1 infection and inhibition in cells were assessed by measuring EGFP fluorescence intensity using Synergy™ H4 Hybrid Multi-Mode Microplate Reader (BioTek Instruments Inc.) with an excitation wavelength of 479 nm and an emission wavelength of 525 nm. The relative fluorescence intensity value is the ratio of the fluorescence intensity of treated cells and untreated cells subtracting the blank (media only) fluorescence intensity.

EGFP-labeled HSV-1 infection and inhibition in mice were examined in the liver, spleen, and kidney tissues. Briefly, indicated tissues of mice were isolated, collected, and washed in cold PBS three times. Tissues were then cut into small pieces, kept on ice and transferred to a homogenizer. Half the tissue in RNAiso Plus kit (Takara Bio, cat. no. 9108) was vigorously vortexed, homogenized and centrifuged (12,000 × *g*, 10 min) at 4 °C for RNA extraction and qPCR assay. Half the tissue in sterile PBS was vigorously vortexed, homogenized and centrifuged as above for titration^68^.

### Sense-and-deletion against viruses mediated by ALICE_Cas9_

The ALICE_Cas9_ device was loaded into HEK293T cells by transfecting pYW274, pYW169, and pYW444 sgRNAs targeting both ADV and HSV-1 genomic DNA 24 h prior to virus incubation. ALICE_Cas9_ cells were incubated with single virus (ADV, MOI = 10; or HSV-1, MOI = 5) or double viruses (simultaneous infection of ADV and HSV-1, MOI = 10 or 5, respectively) for 3 h. The relative viral mRNA expression of *E1B*/*E2 early* genes from ADV, and *UL23*/*UL30* genes from HSV-1 was quantified by qPCR at 2 dpi. All data were normalized to the viral gene expression levels in non-ALICE_Cas9_ cells where HEK-293T cells were co-transfected with pYW274/pWS67/pcDNA3.1 and infected with a single virus (ADV, MOI = 10; or HSV-1, MOI = 5) for 3 h. All primers used are provided in Supplementary Table 2.

### Stable cell lines construction

The monoclonal HEK_ALICE-SEAP-Cas9_ cell line, stably transgenic for HSV-1-inducible SEAP and Cas9 expression, was constructed by co-transfecting HEK-293T (1 × 10^5^ cells) with 400 ng pYW306 (ITR-P_ALICE6_-SEAP-P2A-Cas9-pA::P_mPGK_-puromycin-E2A-STING-PEST-pA-ITR) and 20 ng of the Sleeping Beauty transposase expression vector (P_hCMV_-SB100X-pA), followed by selection in culture medium containing 1 μg/mL puromycin (Thermo Fisher Scientific, cat. no. A1113803) for 10 days. The surviving population was selected for further cultivation and stimulated with HSV-1 (MOI = 0 or 5). Monoclonal cell lines with optimal HSV-1-inducible SEAP and Cas9 production was selected for follow-up studies. Meanwhile, monoclonal cell lines with only optimal HSV-1-inducible SEAP production were selected as HEK_ALICE-SEAP_ cell line for follow-up studies.

The monoclonal HEK_ALICE-Cas9-E317Ab_ cell line, stably transgenic for HSV-1-inducible Cas9 and E317Ab expression, was constructed by transfecting HEK_ALICE-SEAP-Cas9_ (7 × 10^4^ cells) with 200 ng pYW383 (P_ALICE6_-E317Ab-6×His-P2A-mCherry-pA::P_mPGK_-Zeocin-pA), then selected by 1 μg/mL puromycin and 100 μg/mL Zeocin (Thermo Fisher Scientific, cat. no. R25001) for 10 days. The monoclonal cell lines with optimal HSV-1-inducible E317Ab production were selected for follow-up studies.

The monoclonal HEK_ALICE-Cas9-sgRNAs-E317Ab_ cell line, stably transgenic for HSV-1-inducible Cas9 and E317Ab expression, and a constitutive expression of HSV-1-targeted sgRNA_*UL29*_, sgRNA_*UL52*_ and sgRNA_*US8*_, was constructed by transfecting HEK_ALICE-Cas9-E317Ab_ (7 × 10^4^ cells) with 200 ng pYW412 (P_U6_-sgRNA_*UL29*_-sgRNA_*UL52*_-sgRNA_*US8*_-P_SV40_-BSD-pA), then selected by 5 μg/mL blasticidin (Thermo Fisher Scientific, cat. no. A1113903) for 10 days. The monoclonal cell lines with optimal antiviral effects were selected and mixed for follow-up studies.

### qPCR assay

Total RNA in treated HEK-293 cells or in liver, spleen, kidney tissues of treated mice were isolated using the RNAiso Plus kit (Takara Bio, cat. no. 9108). The purified RNA (1 μg) was reversely transcribed into cDNA using PrimeScript^TM^ Reverse Transcription Kit with the gDNA Eraser (Takara Bio, cat. no. RR047). qPCR was performed on LightCycler® 96 instrument (Roche) using SYBR *Premix Ex Taq*^TM^ (Takara Bio, cat. no. RR420) with special primers listed in Supplementary Table 2. The following amplification parameters were used: 95 °C for 10 min, 40 cycles of 95 °C for 30 s, 58 °C for 30 s, and a final cooling at 37 °C for 30 s. The expression of human/mouse housekeeping gene *glyceraldehyde 3-phosphate dehydrogenase* (*GAPDH*) was used as an internal control. The fold change for relative mRNA level was evaluated using the comparative ΔΔCt method^67^.

### T7 endonuclease 1 (T7E1) mismatch detection assay

The deletion efficiency by HSV-1-inducied Cas9 in HEK-293T cells were measured using the primers listed in Supplementary Table 3 as reported^67^. Total genomic DNA of cells was extracted by a TIANamp genomic DNA kit (Tiangen bio, cat. no. DP304) according to the manufacturer’s protocol. sgRNA-targeted *CCR5* gene were PCR-amplified from total genomic DNA using the 2×Taq Plus Master Mix II (Dye Plus) DNA polymerase (Vazyme Inc, cat. no. P213) with the primers listed in Supplementary Table 3. PCR amplicons were purified by a universal DNA purification kit (Tiangen bio, cat. no. DP214). Total 20 μL mixture containing 500 ng purified PCR production and 2 μL 10×M buffer (Takara Bio, cat. no. 1060S) was re-annealed (95 °C for 5 min, then decreased to room temperature) to form heteroduplex DNA. The heteroduplexed DNA was digested using 0.5 μL of T7 endonuclease I (New England BioLabs, cat. no. M0302) and incubated for 2 h at 37 °C. Digested products were separated on a 2% agarose gel. The deletion efficiency by HSV-1-inducied Cas9 was calculated with the following formula: deletion efficiency = 100% × *b*/(*a* + *b*), where a represents the intensity of undigested PCR amplicons and b represents the intensities of the T7E1-digested products.

### Plaque assay

Transfection of pYW274/pYW169 with an HSV-1-targeting sgRNA_*US8*_/sgRNA_*UL29*_/sgRNA_*UL52*_ (pYW102/pYW172/pYW188) or a nonsense sgRNA (pWS68) in HEK-293T cells was performed 20 h prior to EGFP-labeled HSV-1 infection (MOI = 5) for 3 h. After virus incubation for 3 h, supernatant was removed and placed with fresh media containing 1% sterile low melting point agarose (Yeasen Biotech, cat. no.10214ES08). At 48 hpi, solidified agarose medium was removed and HSV-1-infected cells were fixed in 10% trichloroacetic acid (Aladdin, cat. no. 76-03-9) and stained with 0.05% crystal violet (Aladdin, cat. no. 548-62-9). Cells were washed three times in sterile 1×PBS (Sangon Biotech, cat. no. A610100). Micrograph profiling cell activity was performed by microscopy.

### Western blot analysis

Cells infected with or without HSV-1 or cells in the hydrogel-based scaffold isolated from mice were harvested with a RIPA lysis buffer (Yeasen Biotech, cat. no. 20101ES60). The lysates were centrifuged at 10,000 × *g* for 15 min at 4 °C and the protein concentrations were quantified by BCA protein quantification kit (Yeasen Biotech, cat. no. 20201ES76). Proteins samples were loaded on a SDS-PAGE and then electrotransferred onto a polyvinylidene difluoride membrane (PVDF, Millipore, cat. no. ISEQ00010). The membrane was blocked with 5% non-fat milk and incubated with primary antibodies [monoclonal rabbit anti-cGAS (CST, cat. no. 15102T, clone no. D1D3G, 1:1000), monoclonal rabbit anti-STING (CST, cat. no. 13647S, clone no. D2P2F, 1:1000), monoclonal mouse anti-β-Tubulin (Yesen, cat. no. 30301ES40, 1:1000), monoclonal mouse anti-flag (Abcam, cat. no. ab125243, clone no. FG4R, 1:1000), monoclonal rabbit anti-GAPDH (Yesen, cat. no. 30202ES40, 1:2000)]. The membrane was washed 3 times in TBS with 0.05% tween 20 (Sangon biotech, cat. no. 9005-64-5). Immune complexes were detected using secondary antibodies [Alexa fluor-based Goat Anti-Mouse IgG (H + L) (Yesen, cat. no. 33219ES60, 1:25,000), Alexa fluor-based Goat Anti-Rabbit IgG (H + L) (Yesen, cat. no. 33119ES60, 1:25,000)]. Images were scanned using Alpha Innotech Flour Chem-FC2 imaging system (San Leandro)^69^.

### ELISA assay

Expression levels of E317Ab containing a His-tag in culture supernatants or mouse serum were measured using a His-tag ELISA detection kit (GenScript, cat. no. L00436). Mouse serum levels of IL-6, tumor necrosis factor (TNF)-α, interferon (IFN)-γ, and IgG were detected using the LEGEND Max™ mouse IL-6 ELISA kit (BioLegend, cat. no. 431307), mouse TNF-α ELISA kit (MultiSciences, cat. no. 70-EK282/3-96), mouse IFN-gamma ELISA kit (MultiSciences, cat. no. 70-EK280/3-96), and the mouse IgG ELISA kit (MultiSciences, cat. no. 70-EK271-96) according to manufacturer’s instructions.

### NanoLuc assay

NanoLuc levels in cell culture or plasma were measured using the Promega Nano-Glo™ Luciferase Assay System (Promega, cat.no. N1110) according to the manufacturer’s instructions. All assay components (reagents and samples) were equilibrated to room temperature prior to use. Nano-Glo™ Luciferase Assay Reagent was prepared by combining one volume of Nano-Glo™ Luciferase Assay Substrate with 50 volumes of Nano-Glo™ Luciferase Assay Buffer. A volume of reagent equal to that of sample was added to each well. After a 3 min incubation, luminescence was measured using the Synergy™ H4 Hybrid Multi-Mode Microplate Reader (BioTek Instruments Inc.)

### Fluorescence imaging

EGFP expression was measured using an Olympus inverted fluorescence microscope (Olympus IX71, TH4-200) equipped with an Olympus digital camera, a pE-100-LED (CoolLED) as the transmission light source, a Spectra X (Lumencor) as the fluorescent light source, a 10× objective, a 488 nm/509 nm (B/G/R) excitation/emission filter set, and Image-Pro Express C software (version ipp6.0).

### Cytokine and whole blood detection

Mouse serum levels of cytokines, including IL-6, CCL5, CXCL10, TNF-α, and IFN-α, were detected using LEGENDplexTM Multi-Analyte Flow Assay Kit (BioLegend, cat.no. 740625) according to manufacturer’s instructions. The samples were acquired on a BD LSRFortessa^TM^ Flow Cytometer (BD Biosciences) applying a 488 nm laser with 536/40 (BP) filter for the PE fluorochrome and a 638 nm laser with 675/20 (BP) for the APC fluorochrome. The results were analyzed using LEGENDplex™ Data Analysis Software V.8.0 (Vigene Tech Inc., USA). The concentration of each growth factor/cytokine was determined by means of a standard curve generated during the performance of the assay.

The levels of white blood cells, lymphocytes cells, and monocytes cells in mouse blood were measured according to the manufacturer’s protocol by Shanghai Model Organisms Inc.

### Transwell®-based assay

Prevention of virus spread. 5 × 10^4^ immune-like designer cells (ALICE_Cas9_/ALICE_Ab_/ALICE_Cas9+Ab_) or HEK-293T cells (Control) per well incubated with EGFP-labeled HSV-1 (MOI = 5) for 3 h were then separately seeded on transwell polycarbonate membrane inner chambers with an 8 μm pore size (Corning, cat. no. 3428), and then cocultured with HEK-293T cells (5 × 10^4^ cells/well) cultured for 18 h on transwell outer chambers. After 48 h of co-incubation, the fluorescence intensity of HEK-293T cells on outer chambers was detected by the Synergy™ H4 Hybrid Multi-Mode Microplate Reader (BioTek Instruments Inc.).

Prevention of virus infection. HEK-293T cells (5 × 10^4^ cells/well) seeded on the transwell outer chambers, cultured for 18 h, and then infected with EGFP-labeled HSV-1 (MOI = 5) for 3 h. After adding fresh media, the infected cells were cocultured with immune-like designer cells (ALICE_Cas9_/ALICE_Ab_/ALICE_Cas9+Ab_) or HEK-293T cells (Control) (5 × 10^4^ cells/well) seeded on the transwell polycarbonate membrane inner chambers with an 8-μm pore size (Corning, cat. no. 3428). After 48 h of co-incubation, the fluorescence intensity of individual cells on the transwell polycarbonate membrane inner chambers was detected by the Synergy™ H4 Hybrid Multi-Mode Microplate Reader (BioTek Instruments Inc.).

### Sense-and-destroy against HSV-1 mediated by ALICE in mice

To test the HSV-1-inducible transgene expression in mice, 4 × 10^6^ HEK_ALICE-Cas9-E317Ab_ or HEK_ALICE-SEAP-Cas9_ cells were co-transfected with three sgRNAs targeting *US8* (pYW102, 4 μg, P_U6_-sgRNA_*US8*_), *UL29* (pYW172, 4 μg, P_U6_-sgRNA_*UL29*_) and *UL52* (pYW188, 4 μg, P_U6_-sgRNA_*UL52*_), while control cells (4 × 10^6^ HEK_ALICE-Cas9-E317Ab_) were transfected with pcDNA3.1 (12 μg, P_hCMV_-MCS-pA). Wild-type HEK-293T (4 × 10^6^ cells) were transfected with pcDNA3.1 (12 μg). 4 × 10^6^ engineered cells for each mouse were encapsulated into a cylindrical hydrogel-based scaffolds using 300 μL hydrogel scaffold solution (Sigma-Aldrich, cat. no. HYS020).

The female BALB/c wild-type mice (4-week-old; ECNU Laboratory Animal Center) were kept in the animal house maintained at 22 ± 2 °C, with a 12 h light–dark cycle and free access to food and water. To test the autonomous sense-and-destroy against virus of immune-like designer cells (ALICE_Cas9_/ALICE_Ab_/ALICE_Cas9+Ab_) in mice, wild-type female BALB/c mice (four-week-old, ECNU Laboratory Animal Center) were randomly divided into six groups including WT group, sham operation group, control group, ALICE_Cas9_ group, ALICE_Ab_ group, and ALICE_Cas9+Ab_ group: (1) Wild-type BALB/c mice without any treatment were used as negative control (WT); (2) Wild-type BALB/c mice were intraperitoneally implanted with hydrogels containing pYW102/pYW172/pYW188-cotransfected HEK_ALICE-Cas9-E317Ab_ cells without subsequent HSV-1 infection, marked as sham operation group; (3) Control mice were implanted with hydrogels containing pcDNA3.1-transfected wild-type HEK-293T cells; (4) Wild-type BALB/c mice were intraperitoneally implanted with hydrogels containing pYW102/pYW172/pYW188-cotransfected HEK_ALICE-SEAP-Cas9_ cells (ALICE_Cas9_); (5) pcDNA3.1-transfected HEK_ALICE-Cas9-E317Ab_ cells (ALICE_Ab_); and 6) pYW102/pYW172/pYW188-cotransfected HEK_ALICE-Cas9-E317Ab_ cells (ALICE_Cas9+Ab_). One day after implantation, control, ALICE_Cas9_, ALICE_Ab_, and ALICE_Cas9+Ab_ groups were intraperitoneally injected with EGFP-labeled HSV-1 (2 × 10^7^ PFU/mL, 200 μL per mouse) and the sham operation group were intraperitoneally injected with PBS solution (200 μL per mouse).

Mice were retro-orbitally bled at 2, 4, and 6 days post-HSV-1 injection or 30 days post-transplantation. Serum was separated from whole blood by centrifugation at 5000 × *g* for 10 min. Expression levels of E317Ab containing a His-tag in blood were quantified using an his-tag ELISA (GenScript, cat. no. L00436). At 2, 4, and 6 days post-HSV-1 injection or 30 days post-transplantation, mice were euthanized, and the organs (liver, spleen, and kidney) were excised. Virus in tissues was evaluated by titration and qPCR using primers listed in Supplementary Table 2.

### Inhibition of HSV-1 transmission mediated by ALICE_Cas9+Ab_ in mice

To test the ALICE_Cas9+Ab_ mediated inhibition of HSV-1 transmission in mice, HEK_ALICE-Cas9-E317Ab_ cells (4 × 10^6^ cells) co-transfected with three sgRNAs targeting *US8/UL29/UL52* (pYW102/pYW172/pYW188, 4 μg, respectively) were incubated with or without EGFP-labeled HSV-1 (MOI = 1) for 3 h. HEK-293T control cells (4 × 10^6^ cells) transfected with 12 μg pcDNA3.1 were incubated with EGFP-labeled HSV-1 (MOI = 1) for 3 h. 4 × 10^6^ cells of each mouse were encapsulated into a cylinder of hydrogel-based scaffolds using 300 μL hydrogel scaffold solution as described above.

The female BALB/c wild-type mice (4-week-old; ECNU Laboratory Animal Center) were kept in the animal house maintained at 22 ± 2 °C, with a 12 h light-dark cycle and free access to food and water. To test the inhibition of HSV-1 transmission in mice, wild-type female BALB/c mice were randomly divided into four groups: WT, sham operation, control, and ALICE_Cas9+Ab_ groups. (1) Wild-type BALB/c mice without any treatment were used as WT group. (2) Wild-type BALB/c mice intraperitoneally implanted with hydrogels containing pYW102/pYW172/pYW188-co-transfected HEK_ALICE-Cas9-E317Ab_ cells without HSV-1 infection, were marked as sham operation group. (3) Control mice were implanted with hydrogels containing pcDNA3.1-transfected HEK-293T cells with HSV-1 infection. 4) Wild-type BALB/c mice intraperitoneally implanted with hydrogels containing pYW102/pYW172/pYW188-cotransfected HEK_ALICE-Cas9-E317Ab_ cells with HSV-1 infection were marked as ALICE_Cas9+Ab_ group. IL-6 or E317Ab levels in serum were quantified by ELISA. At 2, 4, and 6 days post-transplantation, mice were euthanized, and the organs (liver, spleen, and kidney) were excised. The viral infection and inhibition efficacy in tissues were performed as described in “Sense-and-destroy against HSV-1 mediated by ALICE in mice”.

### Sense-and-destroy against HSV-1 mediated by ALICE_Cas9+Ab_ in a virus-infected mouse model

To establish an HSV-1-infected mouse model, wild-type female BALB/c mice were randomly divided into four groups including WT group, sham operation group, control group, and ALICE_Cas9+Ab_ group. Mice of control group and ALICE_Cas9+Ab_ group were intraperitoneally injected with HSV-1 (2 × 10^7^ PFU/mL, 200 μL per mouse). HEK_ALICE-Cas9-E317Ab_ cells were co-transfected with three sgRNAs targeting *US8/UL29/UL52* (pYW102/pYW172/pYW188, 4 μg, respectively). Wild-type HEK-293T (4 × 10^6^ cells) were transfected with pcDNA3.1 (12 μg). Each mouse implanted with indicated 4 × 10^6^ cells were encapsulated into a cylindrical hydrogel-based scaffolds using 300 μL hydrogel scaffold solution. The female BALB/c wild-type mice (4-week-old; ECNU Laboratory Animal Center) were kept in the animal house maintained at 22 ± 2 °C, with a 12 h light–dark cycle and free access to food and water.

To test the autonomous sense-and-destroy of virus mediated by ALICE_Cas9+Ab_, HSV-1-infected mice were intraperitoneally implanted with hydrogels containing pYW102/pYW172/pYW188-cotransfected HEK_ALICE-Cas9-E317Ab_ cells (ALICE_Cas9+Ab_ group) or pcDNA3.1-transfected HEK-293T cells (control group). Wild-type BALB/c mice were implanted with hydrogels containing ALICE_Cas9+Ab_ cells (sham-operation group). Wild-type BALB/c mice without any treatment were used as negative control (WT group). Serum was collected as described above and E317Ab or IgG levels were quantified by ELISA. At 6 days post-transplantation, mice were euthanized, and the organs (liver, spleen, and kidney) were excised. The viral infection and inhibition efficacy in tissues were examined as described above.

### The herpetic simplex keratitis mouse model

For AAV inoculation, mice anesthetized with ketamine/xylazine were administered the two indicated AAV vectors by RO injection. The female BALB/c wild-type mice (4-week-old; ECNU Laboratory Animal Center) were kept in the animal house maintained at 22 ± 2 °C, with a 12 h light–dark cycle and free access to food and water. AAV vectors marked as AAV-ALICE_SaCas9+Ab_ consisted of two packaged AAV vectors. AAVrh10-ALICE_SaCas9_ carrying an HSV-1-induced SaCas9 and a constitutive expression of HSV-1-targeted *ICP4* sgRNA (pYWG4, ITR-P_ALICE6_-SaCas9-pA::P_U6_-sgRNA_*ICP4*_-ITR; 5 × 10^11^ PFU per mouse) and AAV1-ALICE_Ab_ carrying an HSV-1-induced E317Ab-P2A-nanoLuc and a constitutive expression of STING-PEST (pYW414, ITR-P_ALICE6_-E317Ab-6×His-P2A-NanoLuc-pA::P_hCMV_-STING-PEST-pA-ITR; 5 × 10^11^ PFU per mouse), were simultaneously injected into each mouse (total 100 μL per mouse, 50 μL per eye) via RO injection. All AAVs were packaged by Shanghai Taitool Bioscience Co. Ltd. Equal volumes (total 100 μL per mouse, 50 μL per eye) of PBS solution were injected via RO injection as the control group. The eye, TG, and brain were collected at the indicated time points for further analysis.

For ocular HSV-1 infection, wild-type female BALB/c mice (six-week-old, ECNU Laboratory Animal Center) were anesthetized by intraperitoneal injection of ketamine (100 mg/kg) and xylazine (12 mg/kg) and infected with EGFP-labeled HSV-1 (9 × 10^5^ PFU) following corneal scarification of each eye using a 28-gauge needle.

To test the autonomous sense-and-destroy of HSV-1 mediated by AAV-ALICE_SaCas9+Ab_ in herpetic keratitis mouse model, six-week-old wild-type female BALB/c mice were randomly divided into four groups including WT group, sham operation group, control group, and AAV-ALICE_SaCas9+Ab_ group. Mice were simultaneously RO injection with two packaged AAV vectors (AAVrh10-ALICE_SaCas9_ and AAV1-ALICE_Ab_) 6 days prior to initial HSV-1 infection. Meanwhile, control mice were injected with PBS. At 0 day and 20 days, mice containing AAV-ALICE_SaCas9+Ab_ system were administered with corneal HSV-1 infection (marked as AAV-ALICE_SaCas9+Ab_ group) or non-infection (marked as sham-operation group). Control mice were administered with corneal HSV-1 infection at 0 day and 20 days (marked as control group). Wild-type BALB/c mice without any treatment were used as WT group. Mouse blood was RO collected at 5, 14, and 25 days post-initial HSV-1-infection, and serum was separated as described above. NanoLuc, E317Ab, and IgG levels in serum were quantified using the corresponding detection kits according to manufacturer’s instructions. At 14 and 25-days post-initial HSV-1 infection, mice were euthanized and the organs (eye, TG, and brain) were excised. The viral infection and inhibition efficacy in tissues were evaluated as described in “Sense-and-destroy against HSV-1 mediated by ALICE in mice”.

### Statistical analysis

All in vitro data are expressed as mean ± SD of three independent experiments (*n* = 3 or 4). All micrographs were repeated by three independent experiments with similar results. For the animal experiments, each treatment group consisted of randomly selected mice (*n* = 4 to 9). Neither animals nor samples were excluded from the study. Comparisons between groups were performed using two-tailed Student’s *t* test as means ± SEM. Comparison of the data from multiple groups against one group was performed using a one-way analysis of variance (ANOVA) followed by a Dunnett’s post hoc test or a two-way ANOVA with Bonferroni’s post hoc test. GraphPad Prism software (version 8.3) was used for statistical analysis. *P* values <0.05 were considered statistically significant.

### Reporting summary

Further information on research design is available in the Nature Portfolio Reporting Summary linked to this article.

## Supplementary information

Supplementary Information

Reporting Summary

Peer Review File

## Data Availability

All data associated with this study are present in the manuscript or the Supplementary Materials. All genetic components related to this paper are available with a material transfer agreement and can be requested from H.Y. (hfye@bio.ecnu.edu.cn) or L.-F.W. (linfa.wang@duke-nus.edu.sg). Source data are provided with this paper.

## Source data

Source data files
