# Author Correction: Engineering antiviral immune-like systems for autonomous virus detection and inhibition in mice

**DOI:** 10.1038/s41467-023-39774-x

**Published:** 2023-07-05

**Authors:** Yidan Wang, Ying Xu, Chee Wah Tan, Longliang Qiao, Wan Ni Chia, Hongyi Zhang, Qin Huang, Zhenqiang Deng, Ziwei Wang, Xi Wang, Xurui Shen, Canyu Liu, Rongjuan Pei, Yuanxiao Liu, Shuai Xue, Deqiang Kong, Danielle E. Anderson, Fengfeng Cai, Peng Zhou, Lin-Fa Wang, Haifeng Ye

**Affiliations:** 1 Shanghai Frontiers Science Center of Genome Editing and Cell Therapy, Biomedical Synthetic Biology Research Centre, Shanghai Key Laboratory of Regulatory Biology, Institute of Biomedical Sciences and School of Life Sciences, East China Normal University, Dongchuan Road 500, Shanghai, 200241 China; 2 Chongqing Key Laboratory of Precision Optics, Chongqing Institute of East China Normal University, Chongqing, 401120 China; 3 Programme in Emerging Infectious Diseases, Duke-NUS Medical School, Singapore, Singapore; 4 Department of Breast Surgery, Yangpu Hospital, School of Medicine, Tongji University, 450 Tengyue Road, Shanghai, 200090 China; 5 CAS Key Laboratory of Special Pathogens, Wuhan Institute of Virology, Chinese Academy of Sciences, Wuhan, 430071 Hubei China; 6 University of Chinese Academy of Sciences, Beijing, China; 7 State Key Laboratory of Virology, Wuhan Institute of Virology, Chinese Academy of Sciences, Wuhan, 430071 Hubei China; 8 Department of Biosystems Science and Engineering, ETH Zurich, CH-4058 Basel, Switzerland; 9 SingHealth Duke-NUS Global Health Institute, Singapore, Singapore

**Keywords:** Gene therapy, Synthetic biology

Correction to: *Nature Communications* 10.1038/s41467-022-35425-9, published online 09 December 2022

In the Acknowledgements section of this article the grant number relating to Singapore National Research Foundation was incorrectly given as NRF2016NRF-NSFC002–013 and should have been NRF2018NRF-NSFC003SB-002. The original article has been corrected.

