# Supplementary material for: Engineering antiviral immune-like systems for autonomous virus detection and inhibition in mice
Source: Nat Commun. 2022 Dec 9;13:7629. doi: 10.1038/s41467-022-35425-9 (PMC9734111; doi:10.1038/s41467-022-35425-9)
Supplement: Supplementary file 1 — Supplementary Information [file 41467_2022_35425_MOESM1_ESM.pdf]

Supplementary Information

Engineering antiviral immune-like systems for autonomous virus detection and inhibition in mice

Yidan Wang[1,2], Ying Xu[1], Chee Wah Tan[3], Longliang Qiao[1], Wan Ni Chia[3], Hongyi Zhang[4], Qin Huang[1], Zhenqiang Deng[1], Ziwei Wang[1], Xi Wang[5,6], Xurui Shen[5,6], Canyu Liu[6,7], Rongjuan Pei[7], Yuanxiao Liu[1], Shuai Xue[1,8], Deqiang Kong[1], Danielle E. Anderson[3], Fengfeng Cai[4], Peng Zhou[5], Lin-Fa Wang[3,9*], and Haifeng Ye[1*]

[1]Shanghai Frontiers Science Center of Genome Editing and Cell Therapy, Synthetic Biology and Biomedical Engineering Laboratory, Biomedical Synthetic Biology Research Center, Shanghai Key Laboratory of Regulatory Biology, Institute of Biomedical Sciences and School of Life Sciences, East China Normal University, Dongchuan Road 500, Shanghai 200241, China.

[2]Chongqing Key Laboratory of Precision Optics, Chongqing Institute of East China Normal University, Chongqing 401120, China.

[3]Programme in Emerging Infectious Diseases, Duke-NUS Medical School, Singapore, Singapore.

[4]Department of Breast Surgery, Yangpu Hospital, School of Medicine, Tongji University, 450 Tengyue Road, Shanghai 200090, China.

[5]CAS Key Laboratory of Special Pathogens, Wuhan Institute of Virology, Chinese Academy of Sciences, Wuhan 430071, Hubei, China.

[6]University of Chinese Academy of Sciences, Beijing, China.

[7]State Key Laboratory of Virology, Wuhan Institute of Virology, Chinese Academy of Sciences, Wuhan 430071, Hubei, China

[8]Department of Biosystems Science and Engineering, ETH Zurich, CH-4058 Basel, Switzerland.

[9]SingHealth Duke-NUS Global Health Institute, Singapore, Singapore

[*]To whom correspondence should be addressed: E-mail: hfye@bio.ecnu.edu.cn and linfa.wang@duke-nus.edu.sg

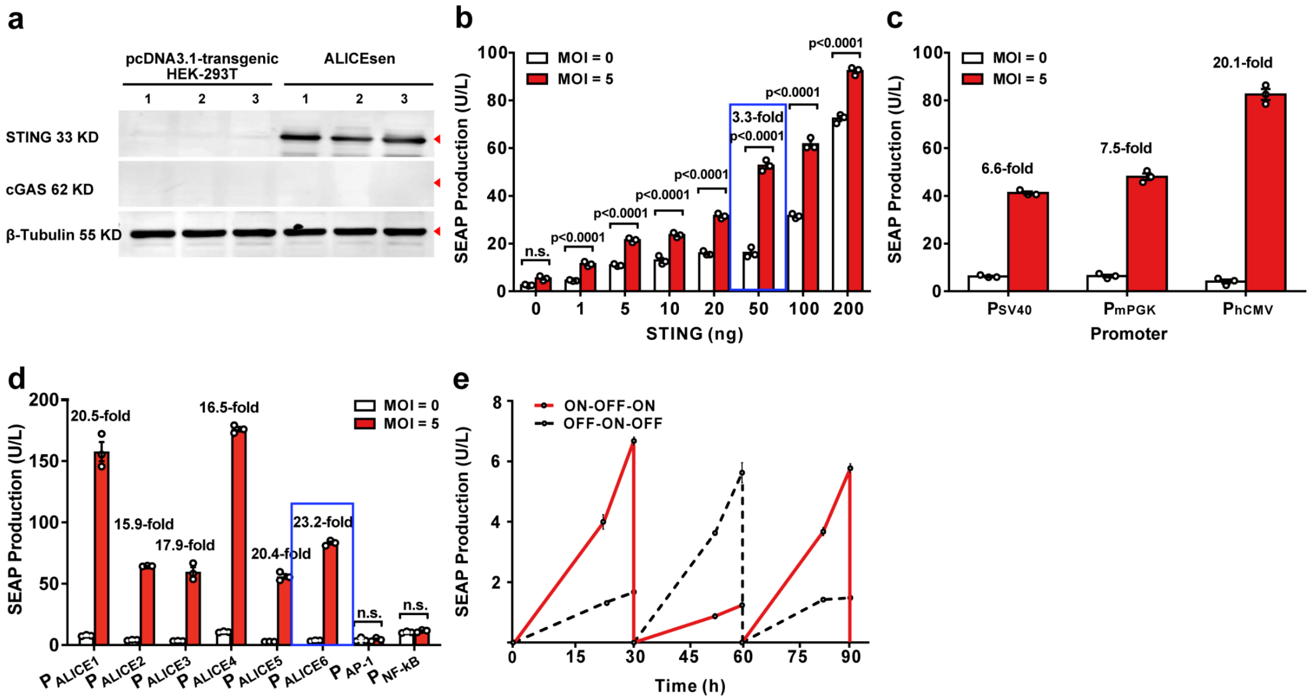

Supplementary Fig. 1 Optimization of ALICE$_{sen}$ in mammalian cells. a Immunoblotting analysis of STING and cGAS expression in HEK-293T cells. HEK-293T cells were tansfected with pcDNA3.1 or pSTING and harvested at 48 hours post-transfection for immunoblotting analysis. Red arrowheads indicate the proteins of interest. **b** Optimization of the adaptor module STING of ALICE$_{sen}$ in mammalian cells. HEK-293T cells co-transfected with the reporter [P$_{ALICE1}$-SEAP-pA; P$_{ALICE1}$, (ISRE)$_5$-P$_{min}$] and different amount of the STING expression vector (P$_{hCMV}$-STING-pA) (0~200 ng) were incubated with HSV-1 (MOI = 0 or 5) for 3 h. SEAP in supernatant was meaasured at 2 days post-infection (dpi). **c** Optimization of the adaptor module (STING-PEST) driven by different promoters in mammalian cells. HEK-293T cells were co-transfected with the reporter (P$_{ALICE1}$-SEAP-pA) and the adaptor module (destabilized-STING, STING-PEST) driven by different promoters including pYW303 (P$_{SV40}$-STING-PEST-pA), pYW302 (P$_{mPGK}$-STING-PEST-pA), or pYW274 (P$_{hCMV}$-STING-PEST-pA). After incubation with HSV-1 (MOI = 0 or 5) for 3 h, SEAP in supernatant was quantified at 2 dpi. **d** Optimization of the virus-responsive promoters (P$_{ALICE\times}$, P$_{AP-1}$, P$_{NF-\kappa B}$) containing different configurations of the operator. HEK-293T cells co-transfected with the adaptor pYW274 (P$_{hCMV}$-STING-PEST-pA) and different reporters including pYW21 [P$_{ALICE1}$-SEAP-pA; P$_{ALICE1}$, (ISRE)$_5$-P$_{min}$], pYW25 (P$_{ALICE2}$-SEAP-pA; P$_{ALICE2}$, (hIFN-RE)-P$_{min}$), pYW26 [P$_{ALICE3}$-SEAP-pA; P$_{ALICE3}$, (hIFN)-P$_{min}$], pYW28 [P$_{ALICE4}$-SEAP-pA; P$_{ALICE4}$, (hIFN-RE)-(ISRE)$_5$-(hIFN-RE)-P$_{min}$], pWS54 [P$_{ALICE5}$-SEAP-pA; P$_{ALICE5}$, (hIFN-RE)-(ISRE)$_3$-P$_{min}$], pWS67 [P$_{ALICE6}$-SEAP-pA; P$_{ALICE6}$,

(hIFN-RE)-(ISRE)$_3$-P$_{min}$], pYW27 (P$_{NF\text{-}kB}$-P$_{min}$-SEAP-pA), or pYW31 (P$_{AP\text{-}1}$-P$_{min}$-SEAP-pA) were incubated with HSV-1 (MOI = 0 or 5) for 3 h and SEAP in supernatant was quantified at 2 dpi. **e** Reversibility of ALICE$_{sen}$. ALICE$_{sen}$ cells were cultivated for 90 h while alternating HSV-1 concentrations either MOI = 0 (OFF) or MOI = 0.1 (ON) at 30 h intervals. ALICE$_{sen}$ cells in the OFF state were incubated with the antiviral drug acyclovir (ACV, 10 μM). SEAP in supernatant was profiled at 18 or 30 h for each interval. All data are expressed as means ± SD; Numbers 1-3 represent three independent experiments; *P* values were calculated by two-way ANOVA with Bonferroni's post hoc test; *n* = 3 independent experiments. n.s., not significant. See Supplementary Table 1 for detailed descriptions of genetic components. Source data are provided as a Source Data file.

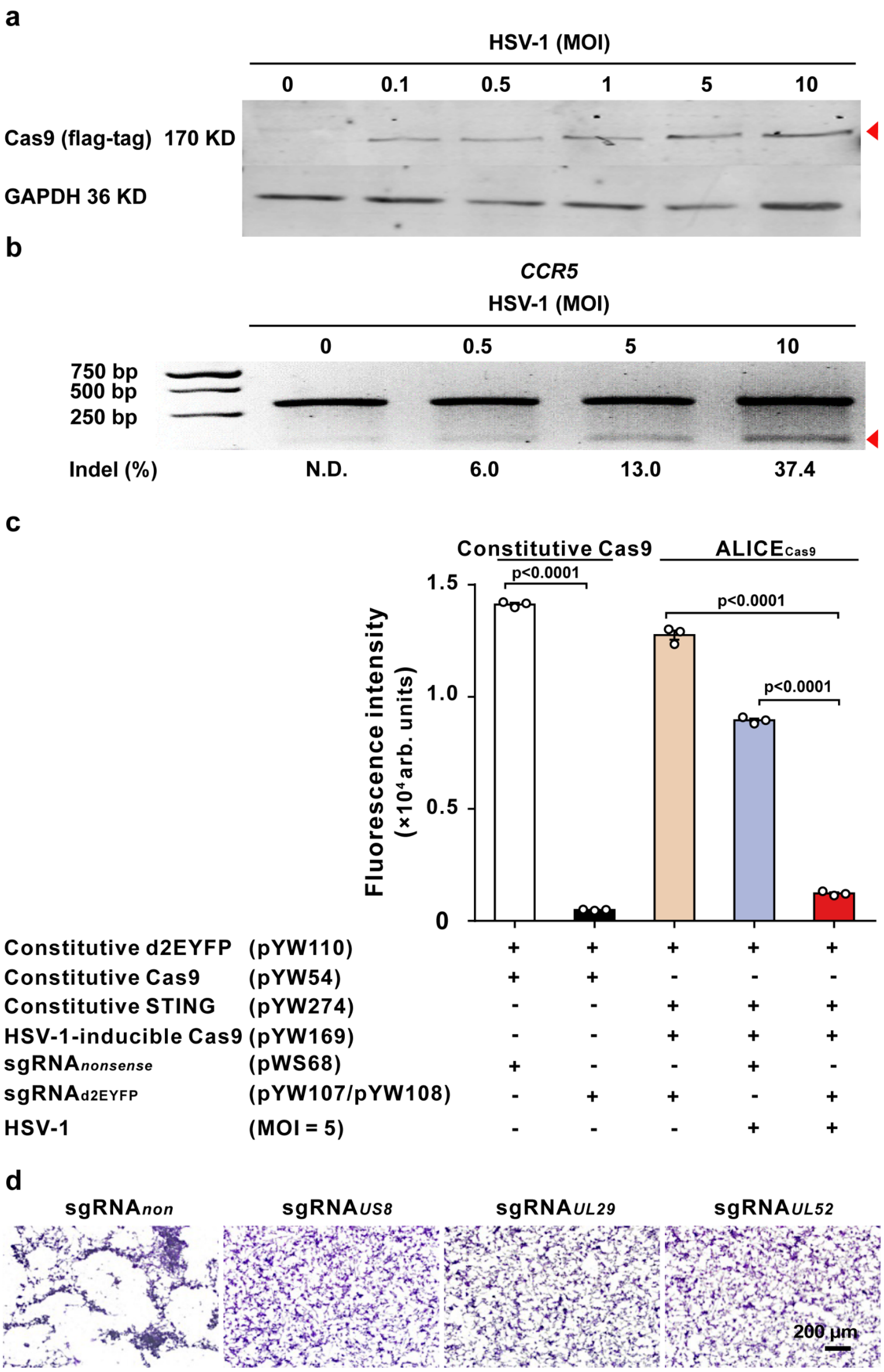

Supplementary Fig. 2 Validation the function of ALICE$_{\text{Cas9}}$ cells. a-b HSV-1-inducible Cas9 expression and gene editing of endogenous gene *CCR5* mediated by ALICE$_{\text{Cas9}}$ cells. HEK-293T cells co-transfected with pYW274 (P$_{\text{hCMV}}$-STING-PEST-pA), pYW169 (P$_{\text{ALICE6}}$-SEAP-P2A-Cas9-pA) and pYW57 (P$_{\text{U6}}$-sgRNA$_{CCR5}$) were infected with various MOI of HSV-1 (MOI = 0~10) for 3 h. At 48 hpi, cells were lysed and subjected to Western blot analysis with the indicated antibody (n = 1 from two independent experiments). **a** The red arrowhead indicates the expected Cas9 expression band. **b** Indel

mutation frequencies (%) of *CCR5* (n = 1 from three independent experiments). Total genomic DNA were extracted and analyzed by the T7 endonuclease 1 (T7E1) mismatch detection assay. The red arrowhead indicates the expected cleavage band. **c** HSV-1-inducible editing of exogenous gene *d2EYFP* mediated by $ALICE_{Cas9}$ cells. HEK-293T cells were co-transfected with constitutive d2EYFP expression vector pYW110 (P_{SV40}-d2EYFP-pA) and other components as indicated. Transgenic cells were infected with HSV-1 (MOI = 0 or 5) for 3 h. At 48 hpi, EGFP expression was analyzed by flow cytometry. **d** HSV-1 plaque assay. Transfection of HSV-1-targeting sgRNA [*US8* locus (pYW102, P_{U6}-$sgRNA_{US8}$); *UL29* locus (pYW172, P_{U6}-$sgRNA_{UL29}$); *UL52* locus (pYW188, P_{U6}-$sgRNA_{UL52}$), and a nonsense control locus (pWS68, P_{U6}-$sgRNA_{nonsense}$)] and pYW274/pYW169 (P_{ALICE6}-SEAP-P2A-Cas9-pA) was performed 20 hours prior to EGFP-labeled HSV-1 infection (MOI = 5) for 3 h in HEK-293T cells. After virus incubation for 3 h, supernatant was removed and replaced with fresh media containing 1% sterile low melting point agarose. At 48 hpi, HSV-1-infected cells were fixed with formaldehyde and stained with crystal violet. Micrographs profiling the plaques were acquired by microscopy. Data in **c** are presented as means ± SD; Data in d are representative of three independent experiments; P values were calculated by one-way ANOVA followed by a Dunnett's post hoc test; n = 3 independent experiments. Detailed descriptions of genetic components in a to d, and transfection mixtures in c are provided in Supplementary Table 1 and 5. Source data are provided as a Source Data file.

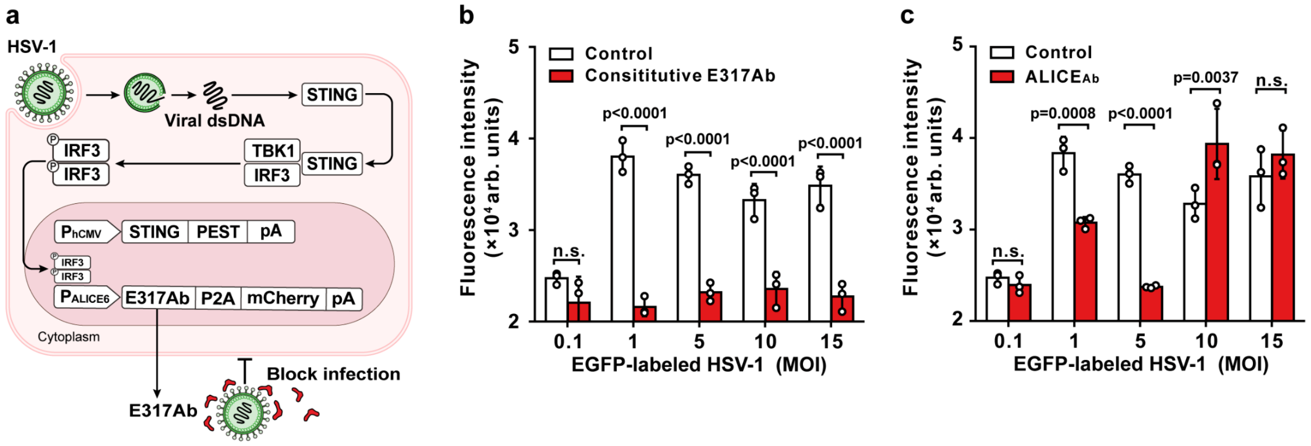

Supplementary Fig. 3 Design and validation of ALICE$_{Ab}$ cells. a Schematic illustration of the viral sense-and-inhibition of ALICE$_{Ab}$ cells. HSV-1 infects mammalian cells and releases dsDNA into the cytoplasm, which activates the engineered STING protein (pYW274). Activated STING triggers the rewired innate immunity signaling pathway mediated by the tank-binding kinase 1 (TBK1), resulting in the phosphorylation and dimerization of interferon regulatory factor 3 (IRF3) that translocates into the nucleus and initiates the expression of E317Ab under the control of its cognate synthetic promoter containing IRF3 binding sites (P_{ALICE6}). The induced E317Ab production further confers resistance to viral infection. **b** Validation of the antiviral effects of constitutive expressed E317Ab. HEK-293T cells were transfected with pYW363 (P_{hCMV}-E317Ab-pA) and the control cells were transfected with pcDNA3.1, and incubated with different MOI of EGFP-labeled HSV-1 (MOI = 0.1~15) for 3 h. EGFP expression was quantified at 2 dpi using a multimode microplate reader. **c** Viral sense-and-inhibition function of ALICE$_{Ab}$ cells. HEK-293T cells were co-transfected with pYW364 (P_{ALICE6}-E317Ab-P2A-SEAP-pA)/pYW274 (P_{hCMV}-STING-PEST-pA). Transgenic cells were infected with different MOI of HSV-1 (MOI = 0.1~15) for 3 h. At 48 hpi, EGFP expression was quantified using a multimode microplate reader. See Supplementary Table 1 for detailed descriptions of genetic components. All data are expressed as means ± SD; *P* values were calculated by two-way ANOVA with Bonferroni's post hoc test; *n* = 3 independent experiments. n.s., not significant. Source data are provided as a Source Data file.

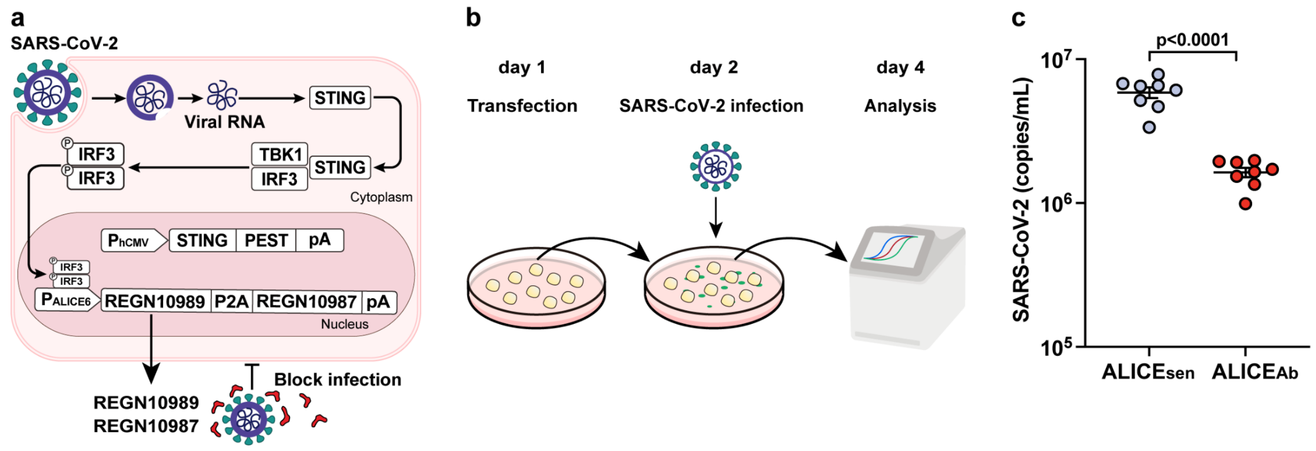

Supplementary Fig. 4 Design and validation of ALICE$_{Ab}$ cells against SARS-CoV-2 infection. a Schematic illustration of the viral sense-and-inhibition of ALICE$_{Ab}$ cells against SARS-CoV-2 infection. During infection, SARS-CoV-2 RNA activates the engineered STING protein (pYW274) and triggers tank-binding kinase 1 (TBK1)-mediated signaling pathway resulting the phosphorylation and dimerization of the interferon regulatory factor 3 (IRF3). IFR3 translocates into the nucleus and initiates the expression of REGN10989 and REGN10987, driven by the synthetic promoter containing IRF3 binding sites (P$_{ALICE6}$). **b** Workflow to assess the anti-SARS-CoV-2 efficacy of ALICE$_{Ab}$ cells in cell culture. HEK-293T cells were co-transfected with ALICE$_{Ab}$ system [ACE2 (P$_{hCMV}$-ACE2-pA)/pYW274 (P$_{hCMV}$-STING-PEST-pA)/pYW406 (P$_{ALICE6}$-REGN10989-P2A-REGN10987-pA)] or ALICE$_{sen}$ [ACE2 (P$_{hCMV}$-ACE2-pA)/pYW274 (P$_{hCMV}$-STING-PEST-pA)/pWS67 (P$_{ALICE6}$-SEAP-pA). Transfected cells were infected with SARS-CoV-2 (MOI = 0.5) for 1 h. At 48 hpi, the mRNA levels of receptor binding domain (RBD) for SARS-CoV-2 spike protein in supernatant were quantified by qPCR assay (c) using primers listed in Supplementary Table 2. See Supplementary Table 1 for detailed description of genetic components. All data are expressed as means ± SD; *P* values were calculated by two-tailed Student's *t* test, *n* = 8 independent experiments. Source data are provided as a Source Data file.

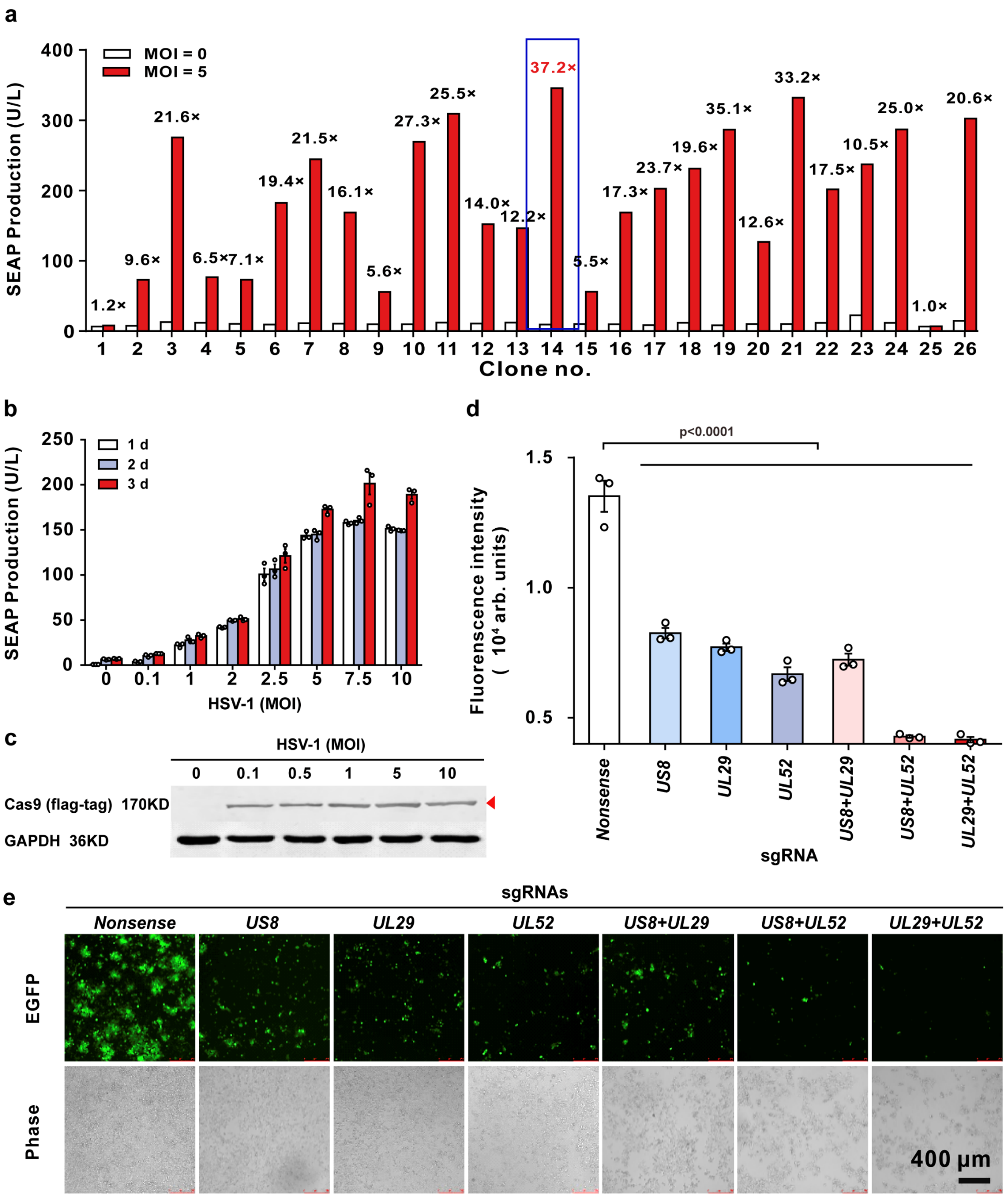

Supplementary Fig. 5 Construction and validation of the ALICE$_{Cas9}$ stable cell lines. a The HEK$_{ALICE-SEAP-Cas9}$ cell line, transgenic for HSV-1-inducible SEAP and Cas9 expression, was constructed by co-transfecting HEK-293T cells with pYW306 (ITR-P$_{ALICE6}$-SEAP-P2A-Cas9-pA::P$_{mPGK}$-puromycin-E2A-STING-PEST-pA-ITR) and SB-100 (P$_{hCMV}$-SB100X), and selected with 1 µg/mL puromycin for 10 days. Twenty-six randomly selected cell clones were infected with HSV-1 (MOI = 0 or 5) for 3 h and profiled for their HSV-1 inducible SEAP production by cultivating for 48

hpi. The blue frame marks the best clone chosen for the following experiments. **b** SEAP expression kinetics of $HEK_{ALICE\text{-}SEAP\text{-}Cas9}$ infected with HSV-1 for 3 h at different MOI (ranging from 0~10). SEAP production in the culture supernatants were scored at 48 hpi. **c** Western blot analysis of HSV-1-inducible Cas9 expression in $HEK_{ALICE\text{-}SEAP\text{-}Cas9}$. The transgenic cells were infected with HSV-1 for 3 h at different MOI (ranging from 0~10) and cultivated for another 48 h before harvesting for Western blot analysis. The red arrowhead indicates the expected Cas9 band. **d-e** Viral sense-and-destroy function of $ALICE_{Cas9}$ in $HEK_{ALICE\text{-}SEAP\text{-}Cas9}$ infected with EGFP-labeled HSV-1. $HEK_{ALICE\text{-}SEAP\text{-}Cas9}$ cells were tranfected/cotransfected with different sgRNAs including single sgRNA respectively targeting to nonsense gene locus (pWS68, P_{U6}-sgRNA$_{nonsense}$), *US8* locus (pYW102, P_{U6}-sgRNA$_{US8}$), *UL29* locus (pYW172, P_{U6}-sgRNA$_{UL29}$), *UL52* locus (pYW188, P_{U6}-sgRNA$_{UL52}$), or dual sgRNAs respectively targeting to *US8* and *UL29* locuses (pYW102/pYW172), *US8* and *UL52* locuses (pYW102/pYW188), *UL29* and *UL52* locuses (pYW172/pYW188). EGFP signal was analyzed by flow cytometry at 48 hpi (d). Fluorescence micrographs profiling EGFP repression kinetics of the antiviral activity were captured with a fluorescence microscope (e). Detailed descriptions of genetic components and transfection mixtures are provided in Supplementary Table 1 and 6. Data in b and d are expressed as means ± SD; Data in c, e are representative of three independent experiments; *P* values were calculated by one-way ANOVA followed by a Dunnett's post hoc test; *n* = 3 independent experiments. Source data are provided as a Source Data file.

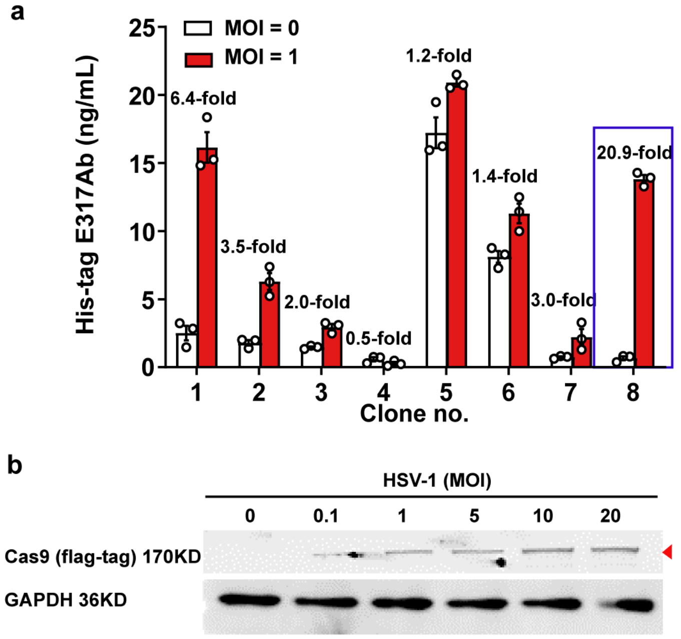

Supplementary Fig. 6 Construction and validation of the ALICE$_{Cas9+Ab}$ stable cell lines. a HSV-1-inducible E317Ab expression in different stable ALICE$_{Cas9+Ab}$ cell lines. HEK$_{ALICE\text{-}SEAP\text{-}Cas9}$ cotransfected with pYW383 (P$_{ALICE6}$-E317Ab-6×His-P2A-mCherry-pA::P$_{mPGK}$-Zeocin-pA) were cultivated for 10 days, selected by Zeocin at 200 μg/mL. Different clones were selected and incubated with HSV-1 (MOI = 0 or 1) for 3 h. The supernatants were collected for E317Ab quantification by ELISA at 2 dpi. **b** HSV-1-inducible Cas9 expression in HEK$_{ALICE\text{-}Cas9\text{-}E317Ab}$. Clone 8 stably integrated with ALICE$_{Cas9+Ab}$ (named as HEK$_{ALICE\text{-}Cas9\text{-}E317Ab}$) was seeded and infected with different MOI of HSV-1 (MOI = 0~20) for 3 h. Total protein from the infected cells were extracted and analyzed by Western blot. The red arrowhead indicates the expected Cas9 band. Data in a are expressed as means ± SD; Data in b are representative of three independent experiments; *n* = 3 independent experiments. Source data are provided as a Source Data file.

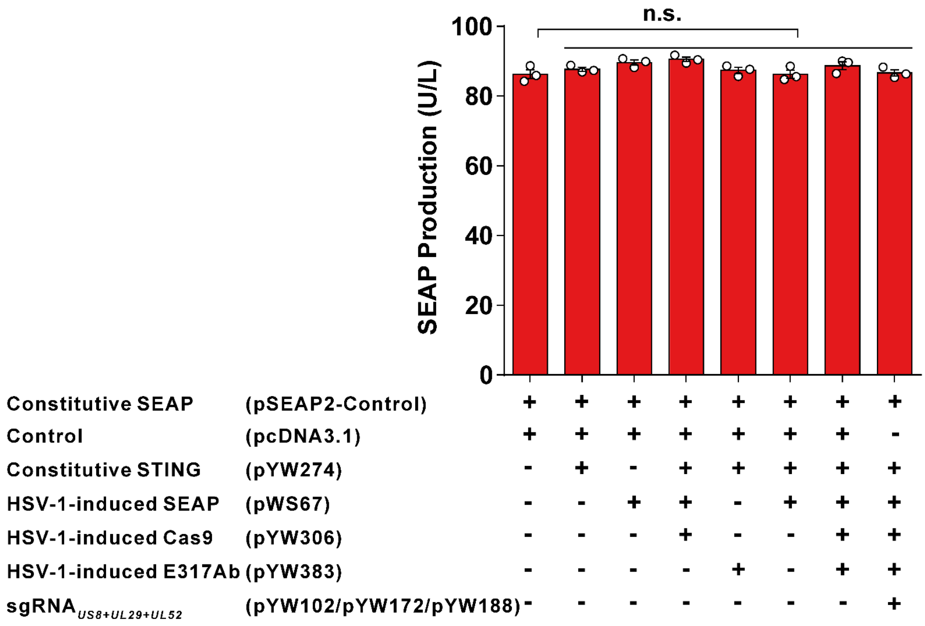

Supplementary Fig. 7 The impact of ALICE's modules on gene expression in HEK-293T cells. HEK-293T cells were co-transfected with 100 ng pSEAP2-Control (P_{SV40}-SEAP-pA) and 100 ng of different combinations (pcDNA3.1, pYW274, pWS67, pYW306, pYW383, pYW102, pYW172, and pYW188) as indicated. Control cells were co-transfected with pSEAP2-Control and pcDNA3.1. SEAP in supernatant was measured at 2 days post-transfection. Detailed descriptions of genetic components and transfection mixtures are provided in Supplementary Table 1 and 7. All data are expressed as means ± SD; *P* values for all other group versus Control group (the first bar); *P* values were calculated by one-way ANOVA followed by a Dunnett's post hoc test; *n* = 3 independent experiments. n.s., not significant. Source data are provided as a Source Data file.

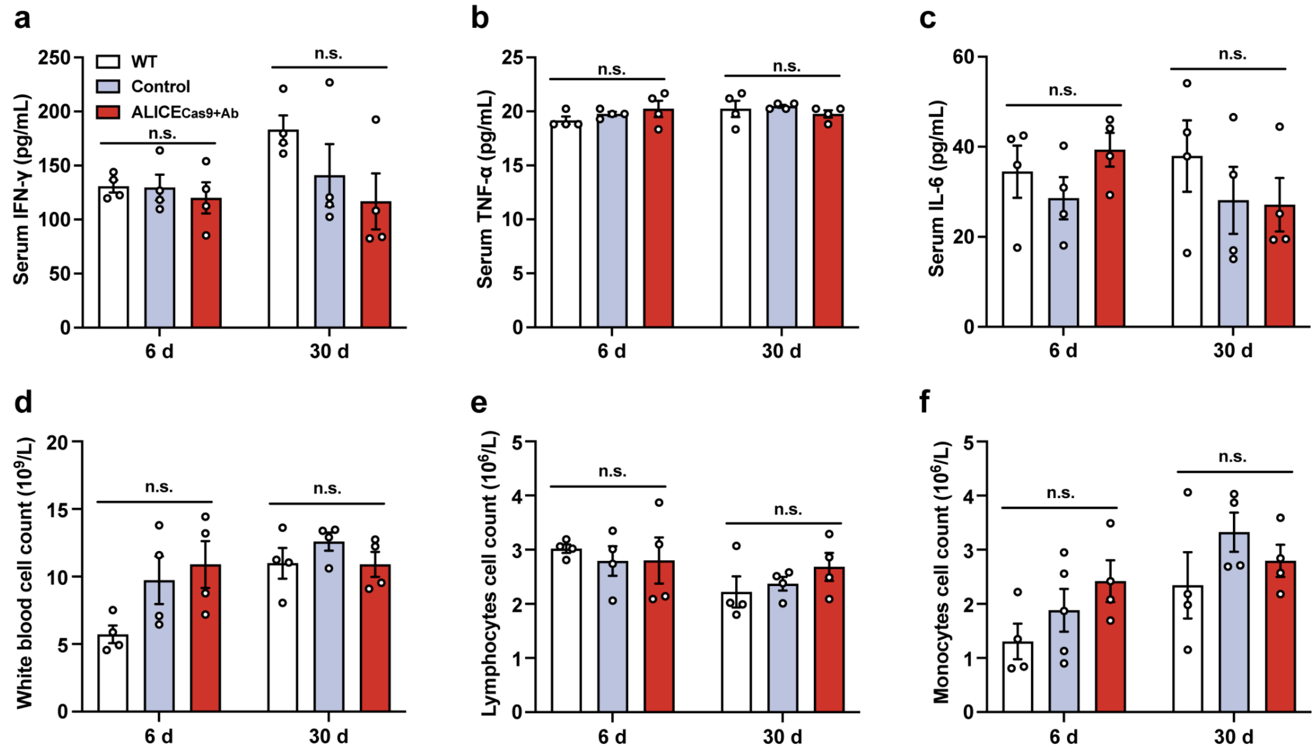

Supplementary Fig. 8 Serum inflammatory cytokines and whole blood analysis of mice implanted with hydrogel implants containing designer cells. Wild-type BALB/c mice were intraperitoneally implanted with hydrogel implants containing three sgRNAs (targeting *US8*, *UL29* and *UL52*)-transgenic HEK$_{ALICE\text{-}Cas9\text{-}E317Ab}$ cells (ALICE$_{Cas9+Ab}$) for 6 or 30 days. Control mice were implanted with hydrogels containing pcDNA3.1-transgenic HEK-293T cells, while wild-type BALB/c mice without hydrogel implantation were used as negative control (WT). 6 or 30 days after implantation, mouse blood was collected for quantification of (a) IFNγ, (b) TNF-α, (c) IL-6 production by ELISA. **d** Counts for total white blood cells (WBC), (e) blood lymphocytes and (f) blood monocytes in mice. White bars (WT), blue-grey bars (Control), red bars (ALICE$_{Cas9+Ab}$) in a-f. Data are expressed as mean ± SEM; *P* values were calculated by two-tailed Student's *t* test; *n* = 4 mice in a-e, *n* = 4 or 5 mice in f; n.s., not significant. Source data are provided as a Source Data file.

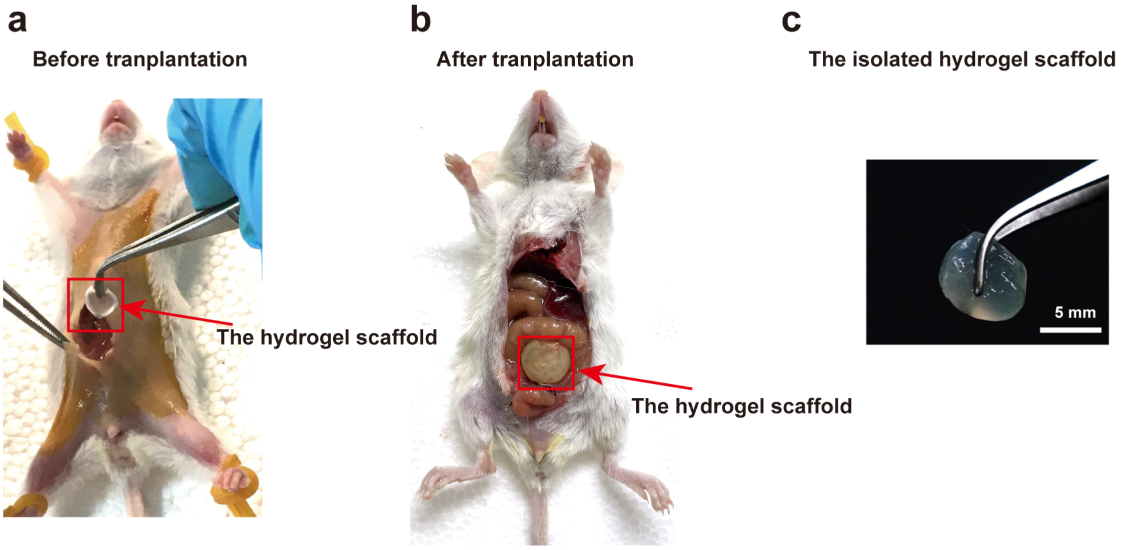

Supplementary Fig. 9 The location of the hydrogel scaffold containing ALICE$_{Cas9+Ab}$ cells in a mouse. a Photograph of the hydrogel scaffold containing ALICE$_{Cas9+Ab}$ cells before transplantation. **b** Photograph of the hydrogel scaffold containing ALICE$_{Cas9+Ab}$ cells after transplantation. At 30 days post-transplantation, mice were euthanized and dissected. The hydrogel scaffold was surrounded by the intestine and completely located in the abdominal cavity. **c** An enlarged view of the isolated hydrogel scaffolds at 30 days post-transplantation. All photos are representative of four independent experiments;

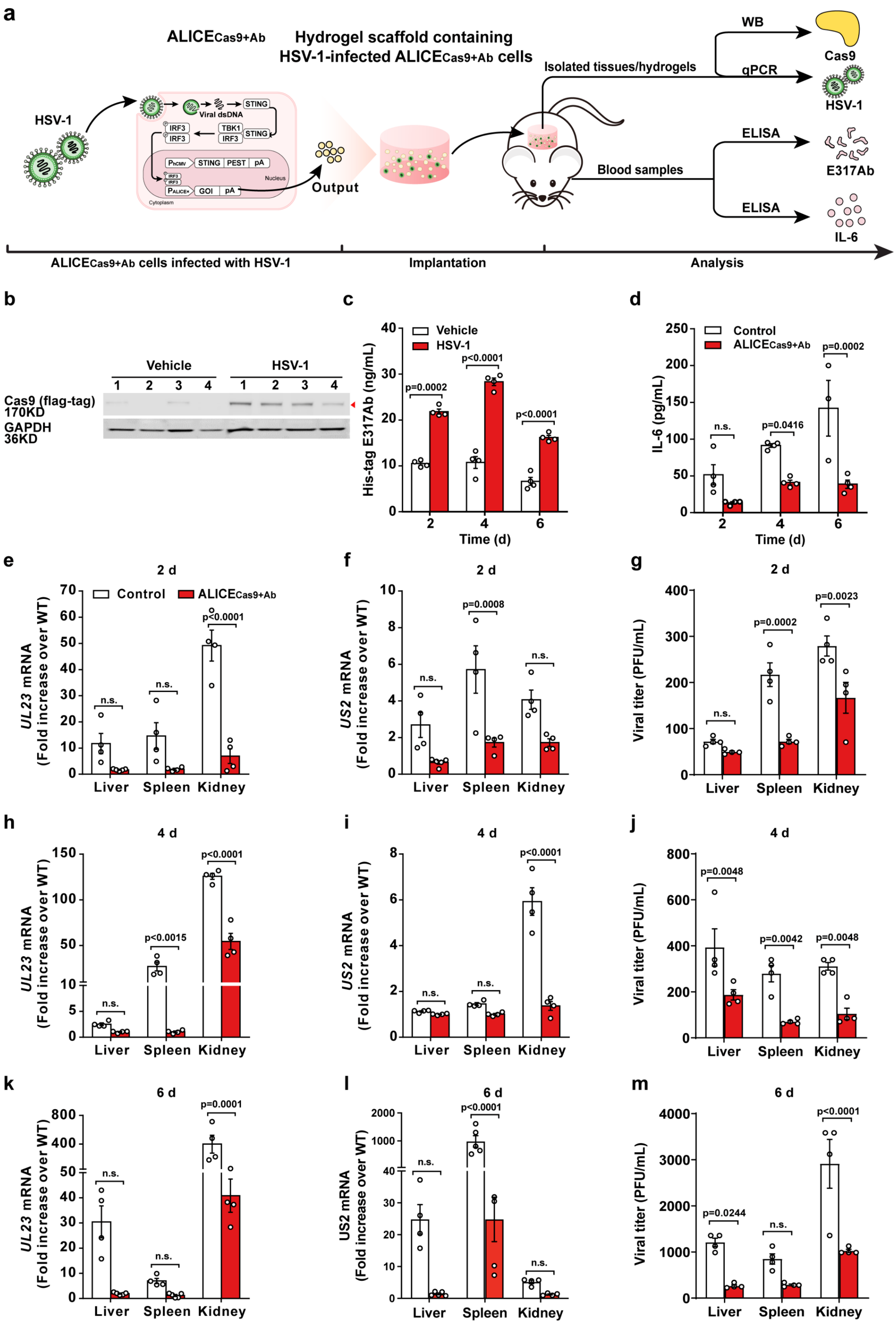
a
ALICECas9+Ab
Hydrogel scaffold containing
HSV-1-infected ALICECas9+Ab cells
HSV-1
Viral dsDNA
STING
IRF3
TBK1
PCMV
PEST
pA
Nucleus
PALICE
GOI
Cytoplasm
Output
Isolated tissues/hydrogels
Blood samples
WB
qPCR
ELISA
Cas9
E317Ab
IL-6
ALICECas9+Ab cells infected with HSV-1
Implantation
Analysis
b
Vehicle
HSV-1
1 2 3 4
Cas9 (flag-tag)
170KD
GAPDH
36KD
c
Vehicle
HSV-1
His-tag E317Ab (ng/mL)
p=0.0002
p<0.0001
p<0.0001
Time (d)
d
Control
ALICECas9+Ab
IL-6 (pg/mL)
n.s.
p=0.0416
p=0.0002
Time (d)
e
2 d
UL23 mRNA
(Fold increase over WT)
p<0.0001
Liver
Spleen
Kidney
f
2 d
US2 mRNA
(Fold increase over WT)
p=0.0008
g
2 d
Viral titer (PFU/mL)
p=0.0002
p=0.0023
h
4 d
p<0.0015
p<0.0001
i
4 d
p<0.0001
j
4 d
p=0.0048
p=0.0042
p=0.0048
k
6 d
p=0.0001
l
6 d
p<0.0001
m
6 d
p=0.0244
p<0.0001

Supplementary Fig. 10 Validation the inhibition of virus transmission mediated by ALICE$_{Cas9+Ab}$ in mice. a Schematic illustration of ALICE$_{Cas9+Ab}$ mediated inhibition of HSV-1 transmission in mice. Three sgRNAs (targeting *US8*, *UL29* and *UL52*)-transgenic HEK$_{ALICE\text{-}Cas9\text{-}E317Ab}$ cells (ALICE$_{Cas9+Ab}$) or unmodified HEK-293T cells (Control) were infected with EGFP-labeled HSV-1 and then encapsulated into a hydrogel-based scaffold, which was then transplanted into the abdomen of mice by intraperitoneal surgery. Total RNA was extracted from isolated tissues for qPCR analysis. Total protein was extracted from hydrogel-based scaffolds for Western blot analysis. Blood samples were collected for ELISA detection of E317Ab and IL-6. **b** Western blot analysis of HSV-1-inducible Cas9 expression in hydrogel-based scaffolds and **c** validation of HSV-1-inducible E317Ab expression. Three sgRNAs (targeting *US8*, *UL29* and *UL52*)-transgenic HEK$_{ALICE\text{-}Cas9\text{-}E317Ab}$ cells were infected with EGFP-labeled HSV-1 [MOI = 0 (Vehicle group) or 1 (HSV-1 group)] and then encapsulated into a hydrogel-based scaffold, which was then transplanted into the abdomen of mice. Total proteins from hydrogels isolated from mice were extracted for Western blot analysis at 6 days post-implantation. The red arrowhead indicates the expected Cas9 band. E317Ab production levels in the blood were analyzed at 2, 4 and 6 days post-implantation. **d** Validation of HSV-1-inducible IL-6 expression. IL-6 production levels in the bloodstream were analyzed at 2, 4 and 6 days post-implantation. **e** qPCR assay of HSV-1 *UL23/US2* mRNA in liver/spleen/kidney at 2 (e-f), 4 (h-i), 6 (k-l) days post-transplantation. The mice were processed as described in a. The relative expression values were calculated using the ΔΔct method based on the expression levels of host genes *UL23/US2* in organs of WT mice without intraperitoneal injection of hydrogel. White bars (Control), red bars (ALICE$_{Cas9+Ab}$) in e-m. **g** Viral titers in mice. The mice were processed as described in a. Virus liver, spleen, kidney was titrated at 2, 4 and 6 days (g, j, m) post-transplantation. Numbers 1-4 represent four indenpent mice in b. Data are expressed as mean ± SEM; *P* values were calculated by two-way ANOVA with Bonferroni's post hoc test; *n* = 4 mice. Source data are provided as a Source Data file.

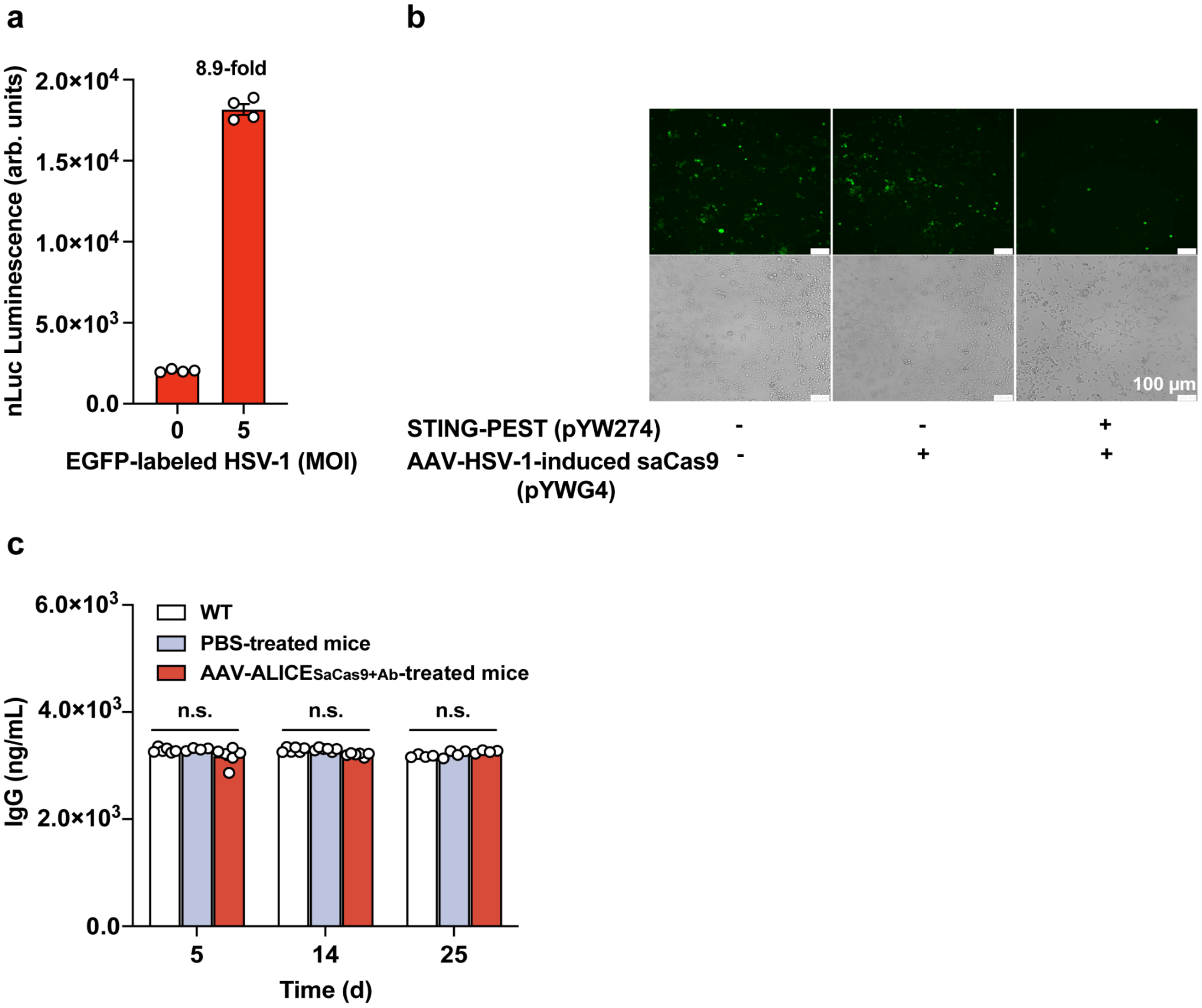

Supplementary Fig. 11 Validation the virus responsiveness of ALICE$_{Cas9+Ab}$ via AAV-mediated gene therapy. a Validation of HSV-1-induced nanoLuc expression in HEK-293T cells. HEK-293T cells transfected with the plasmid pYW414 (200 ng, AAV1-ALICE$_{Ab}$ carrying a HSV-1-induced E317Ab and NanoLuc and a constitutive expression of STING-PEST) were incubated with EGFP-labeled HSV-1 (MOI = 0 or 5) for 3 h. NanoLuc production in the culture supernatants were scored at 2 dpi. **b** Validation of HSV-1-induced SaCas9 expression in HEK-293T cells. HEK-293T cells co-transfected with the plasmid pYWG4 (150 ng, AAVRh10-ALICE$_{SaCas9}$ carrying a HSV-1-induced SaCas9 and a constitutive expression of HSV-1-targeted *ICP4* sgRNA) and pYW274 (150 ng, P$_{hCMV}$-STING-PEST-pA). Transfected cells were infected with EGFP-labeled HSV-1 (MOI = 0 or 5) for 3 h. Fluorescence micrographs profiling the kinetic of EGFP expression were obtained with a fluorescent microscope at 2 dpi. **c** Validation of IgG expression in a herpetic simplex keratitis mouse model. IgG production levels in the blood were analyzed by using an IgG ELISA at 5, 14 and 25 days post initial

HSV-1 infection. Data are expressed as mean ± SEM; *P* values were calculated by two-tailed Student's *t* test; *n* = 4 mice in a; Data in b are representative of three independent experiments; *n* = 4-6 mice in c. n.s., not significant. See Supplementary Table 1 for detailed descriptions of genetic components. Source data are provided as a Source Data file.

Supplementary Table 1. Plasmids designed and used in this study.

Plasmids	Detailed description	Reference
pcDNA3.1(+)	Constitutive mammalian P_{hCMV}-driven expression vector (P_{hCMV}-MCS-pA)	Invitrogen, CA
pSEAP2-Control	Constitutive mammalian P_{SV40}-driven SEAP expression vector (P_{SV40}-SEAP-pA)	Clontech, CA
P_{hCMV}-SB100X	Constitutive mammalian P_{hCMV}-driven SB100X expression vector (P_{hCMV}-SB100X-pA)	Addgene (no. 34879)
pSBtet-GP	The tetracycline-responsive luciferase expression vector (ITR-Ptet operator-luciferase-pA::P_{mPGK}-PuroR-pA-ITR)	Addgene (no. 60495)
ACE2	Constitutive mammalian P_{hCMV}-driven ACE2 expression vector (P_{hCMV}-ACE2-pA)	Gifted by Prof. Zhou
pWS54	HSV-1-inducible SEAP expression vector [P_{ALICE5}-SEAP-pA; P_{ALICE5}, (hIFN-RE)-$(ISRE)_3$-P_{min}]	This work
pWS67	HSV-1-inducible SEAP expression vector [P_{ALICE6}-SEAP-pA; P_{ALICE6}, (hIFN-RE)-$(ISRE)_3$-P_{min}]	This work
pWS68	Constitutive mammalian expression vector for sgRNA non-targeting (P_{U6}-sgRNA$_{non}$)	This work
pXU1	HSV-1-inducible E317Ab expression vector (P_{ALICE6}-SEAP-pA)	This work
STING	Constitutive mammalian P_{hCMV}-driven STING expression vector (P_{hCMV}-STING-pA)	This work
pYW21	HSV-1-inducible SEAP expression vector [P_{ALICE1}-SEAP-pA; P_{ALICE1}, $(ISRE)_5$-P_{min}]	This work
pYW25	HSV-1-inducible SEAP expression vector [P_{ALICE2}-SEAP-pA; P_{ALICE2}, (hIFN-RE)-P_{min}]	This work
pYW26	HSV-1-inducible SEAP expression vector [P_{ALICE3}-SEAP-pA; P_{ALICE3}, (hIFN)-P_{min}]	This work
pYW27	HSV-1-inducible SEAP expression vector ($P_{NF\text{-}kB}$-P_{min}-SEAP-pA)	This work
pYW28	HSV-1-inducible SEAP expression vector [P_{ALICE4}-SEAP-pA; P_{ALICE4}, (hIFN-RE)-$(ISRE)_5$-(hIFN-RE)-P_{min}]	This work
pYW31	HSV-1-inducible SEAP expression vector ($P_{AP\text{-}1}$-P_{min}-SEAP-pA)	This work
pYW57	Constitutive mammalian expression vector for sgRNA targeting to *CCR5* (P_{U6}-sgRNA$_{CCR5}$)	This work
pYW66	HSV-1-inducible E317Ab expression vector [P_{ALICE7}-E317Ab-pA; P_{ALICE7}, (hIFN-RE)-$P_{hCMVmin}$]	This work
pYW102	Constitutive mammalian expression vector for sgRNA targeting to *US8* (P_{U6}-sgRNA$_{US8}$)	This work
pYW107	Constitutive mammalian expression vector for sgRNA targeting to *d2EYFP* (P_{U6}-sgRNA$_{d2EYFP1}$)	This work
pYW108	Constitutive mammalian expression vector for sgRNA targeting to *d2EYFP* (P_{U6}-sgRNA$_{d2EYFP2}$)	This work

pYW110	Constitutive mammalian P_{SV40}-driven d2EYFP expression vector (P_{SV40}-d2EYFP-pA)	This work
pYW169	HSV-1-inducible SEAP-P2A-Cas9 expression vector (P_{ALICE6}-SEAP-P2A-Cas9-pA)	This work
pYW172	Constitutive mammalian expression vector for sgRNA targeting to *UL29* (P_{U6}-sgRNA$_{UL29}$)	This work
pYW188	Constitutive mammalian expression vector for sgRNA targeting to *UL52* (P_{U6}-sgRNA$_{UL52}$)	This work
pYW274	Constitutive mammalian P_{hCMV}-driven STING expression vector (P_{hCMV}-STING-PEST-pA)	This work
pYW292	HSV-1-inducible SEAP-P2A-E317Ab-Fc expression vector (P_{ALICE6}-SEAP-P2A-E317Ab-Fc-pA)	This work
pYW293	HSV-1-inducible E317Ab-Fc-P2A-SEAP expression vector (P_{ALICE6}-E317Ab-Fc-P2A-SEAP-pA)	This work
pYW302	Constitutive mammalian P_{mPGK}-driven STING expression vector (P_{mPGK}-STING-PEST-pA)	This work
pYW303	Constitutive mammalian P_{SV40}-driven STING expression vector (P_{SV40}-STING-PEST-pA)	This work
pYW306	HSV-1-inducible SEAP-P2A-Cas9 expression vector (ITR-P_{ALICE6}-SEAP-P2A-Cas9-pA::P_{mPGK}-puromycin-E2A-STING-PEST-pA-ITR)	This work
pYW327	Virus-inducible SEAP-P2A-hIFN-β expression vector (P_{ALICE6}-SEAP-P2A-hIFN-β-pA)	This work
pYW363	Constitutive mammalian P_{hCMV}-driven E317Ab expression vector (P_{hCMV}-E317Ab-pA)	This work
pYW364	HSV-1-inducible E317Ab-P2A-SEAP expression vector (P_{ALICE6}-E317Ab-P2A-SEAP-pA)	This work
pYW365	Virus-inducible SEAP-P2A-hIFN-α expression vector (P_{ALICE6}-SEAP-P2A-hIFN-α-pA)	This work
pYW366	Constitutive mammalian P_{hCMV}-driven E317Ab-Fc expression vector (P_{hCMV}-E317Ab-Fc-pA)	This work
pYW379	HSV-1-inducible EGFP expression vector (P_{ALICE6}-EGFP-pA)	This work
pYW380	Constitutive mammalian P_{hCMV}-driven E317Ab-6×His expression vector (P_{hCMV}-E317Ab-6×His-pA)	This work
pYW383	HSV-1-inducible E317Ab-6×His-P2A-mCherry expression vector (P_{ALICE6}-E317Ab-6×His-P2A-mCherry-pA::P_{mPGK}-Zeocin-pA)	This work
pYW406	HSV-1-inducible REGN10989-P2A-REGN10987 expression vector (P_{ALICE6}-REGN10989-P2A-REGN10987-pA)	This work
pYW412	Constitutive mammalian expression vector for sgRNA targeting to *UL29*, *UL52*, *US8* and P_{SV40}-driven BSD expression vector (P_{U6}-sgRNA$_{UL29}$-sgRNA$_{UL52}$-sgRNA$_{US8}$-P_{SV40}-BSD-pA	This work
pYW414	HSV-1-inducible E317Ab-6×His-P2A-NanoLuc expression vector (ITR-P_{ALICE6}-E317Ab-6×His-P2A-nanoLuc-pA::P_{SV40}-STING-	This work

	PEST-pA-ITR)	
pYW444	Constitutive mammalian expression vector for sgRNA targeting to *E1A* and *UL52* (P_{U6}-sgRNA$_{E1A\#1+UL52}$)	This work
pYWG4	HSV-1-inducible SaCas9 expression vector (ITR-P_{ALICE6}-SaCas9-pA::P_{U6}-sgRNA$_{ICP4}$-ITR)	This work

Abbreviations: AAV, adeno-associated virus; **Ab**, antibody; **ACV**, acyclovir; **ADV**, adenovirus; **arb. units**, arbitrary units; **Cas9**, CRISPR associated protein 9; ***CCR5***, C-C chemokine receptor type 5 gene; **CMV**, cytomegalovirus; **CPE**, cytopathic effect; **CRISPR**, clustered regularly interspaced short palindromic repeats; **dpi**, day post-infection; **d2EYFP**, enhanced yellow fluorescent protein *in vivo* half-life of ~2 h; **ECNU**, East China Normal University; **EGFP**, enhanced green fluorescent protein; **E2A**, equine rhinitis A virus-derived self-cleaving peptide engineered for bicistronic gene expression in mammalian cells; **Flag**, a polypeptide epitope containing three DYKDDDDK repeats; ***GAPDH***, glyceraldehyde 3-phosphate dehydrogenase; **HBV**, hepatitis B virus; **HCV**, hepatitis C virus; **hIFN**, human interferon response element; **hIFN-RE**, synthetic human interferon response element; **HIV**, human immunodeficiency virus; **hpi**, hour post-infection; **HPV**, human papillomavirus; **HSK**, herpetic simplex keratitis; **HSV-1**, herpes simplex virus type 1; **HSV-2**, herpes simplex virus type 2; **ICP4**, HSV-1 immediate-early regulatory protein ICP4; **IgG**, Immunoglobulin G; **ISRE**, IFN-stimulated response element; **ITR**, inverted terminal repeats of SB100X; **MCS**, multiple cloning site; ***mGAPDH***, mouse housekeeping gene *GAPDH*; **MOI**, multiplicity of infection; **pA**, polyadenylation signal; **P_{ALICE}**, HSV-1-inducible operator; **PEST**, proteolytic tag; **PFU**, plaque-forming unit; **P_{hCMV}**, human cytomegalovirus immediate early promoter; **$P_{hCMVmin}$**, minimal version of P_{hCMV}; **P_{min}**, minimal eukaryotic TATA-box promoter (5'-TAGAGGGTATATAATGGAAGCTCGACTTCCAG-3'); **P_{mPGK}**, mouse phosphoglycerate kinase gene promoter; **P_{SV40}**, simian virus 40 promoter; **P_{U6}**, U6 promoter; **P2A**, picornavirus-derived self-cleaving peptide engineered for bicistronic gene expression in mammalian cells; **RBD**, receptor binding domain for SARS-CoV-2 Spike protein; **SaCas9**, *staphylococcus aureus* Cas9; **SB100X**, the Sleeping Beauty transposase; **SEAP**, human placental secreted alkaline phosphatase; **sgRNA**, small guide RNA; **STING**, stimulator of interferon genes; **$TCID_{50}$**, 50% tissue culture infective dose; **TG**, trigeminal ganglia; ***UL23***, thymidine kinase; ***UL29***, single-stranded DNA-binding protein; ***UL30***, DNA polymerase catalytic subunit; ***UL52***, helicase-primase primase subunit; ***US2***, virion protein US2; ***US8***, envelope glycoprotein E; **vge**, viral genome equivalents; **VSV**, Vesicular stomatitis virus.

Supplementary Table 2. Primers used for qPCR analysis.

Target Gene	Forward primer	Reverse primer
RBD	CAATGGTTTAACAGGCACAGG	CTCAAGTGTCTGTGGATCACG
HCV genomicRNA	ATCACTCCCCTGTGAGGAACT	GCGGGTTGATCCAAGAAAGG
HBV pgRNA	GCCTTAGAGTCTCCTGAGCA	GAGGGAGTTCTTCTTCTAGG
E1B	GGTGAGATAATGTTTAACTTGC	TAACCAAGATTAGCCCACGG
E2 early	TACTGCGCGCTGACTCTTAAGG	ATGGCGCTGACAACAGGTGCT
UL23	GATCGTCGGTATGGAGCCTG	CCCAACGGCGACCTGTATAA
UL30	TGTTTCGCGTGTGGGACATA	TTGTCCTTCAGGACGGCTTC
US2	GAGGGTCAGTTTCTTGCGGA	GTCCGTGTTGTGCGTGTATG
GAPDH	GGAGCGAGATCCCTCCAAAAT	GGCTGTTGTCATACTTCTCATGG
mGAPDH	GAAGGGCTCATGACCACAGT	GGATGCAGGGATGATGTTCT

Supplementary Table 3. Primers used for PCR amplification.

Target Gene	Primer name	Sequence
CCR5	1st PCR-Forward	CTCCATGGTGCTATAGAGCA
	2st PCR-Forward	GAGCCAAGCTCTCCATCTAGT
	Reverse	GCCCTGTCAAGAGTTGACAC

Supplementary Table 4. Sequences of sgRNA.

Target	Target gene	Sequence
HEK-293T	*CCR5*	GTGACATCAATTATTATACAT
HSV-1	*US8*	TATCCCCGGGACATGGATCG
	UL29	GCGAGCGTACACGTATCCC
	UL52	GCCGTCGGTCGCATAAAGCG
ADV	*E1A*	ACCTCCTGAGATACACCCGG

Supplementary Table 5. The expression vectors, transfection mixtures and HSV-1 used in Supplementary Fig. 2c

		Constitutive Cas9		ALICE$_{Cas9}$		
Modules	Plasmids (ng)	1	2	3	4	5
Constitutive d2EYFP	pYW110	50	50	50	50	50
Constitutive Cas9	pYW54	150	150	/	/	/
Constitutive STING-PEST	pYW274	/	/	50	50	50
HSV-1-induced Cas9	pYW169	/	/	150	150	150
sgRNA$_{nonsense}$	pWS68	100	/	/	100	/
sgRNA$_{d2EYFP}$	pYW107/ pYW108	/	50/50	50/50	/	50/50
pcDNA3.1	pcDNA3.1	50	50	/	/	/
HSV-1	/	/	/	/	+	+

Supplementary Table 6. The expression vectors and transfection mixtures used in Supplementary Fig. 5d

sgRNA	Plasmids (ng)	1	2	3	4	5	6	7
/	pcDNA3.1	/	50	50	50	/	/	/
sgRNA$_{nonsense}$	pWS68	100	50	/	/	/	/	/
sgRNA$_{US8}$	pYW102	/	/	50	/	50	50	/
sgRNA$_{UL29}$	pYW172	/	/	/	50	50	/	50
sgRNA$_{UL52}$	pYW188	/	/	/	/	/	50	50

Supplementary Table 7. The expression vectors and transfection mixtures used in Supplementary Fig. 7

Modules of ALICE	Plasmids (ng)	1	2	3	4	5	6	7	8
Constitutive SEAP	pSEAP2-Control	100	100	100	100	100	100	100	100
Control	pcDNA3.1	550	500	400	350	350	350	150	/
Constitutive STING-PEST	pYW274	/	50	/	/	/	50	/	/
HSV-1-induced SEAP	pWS67	/	/	150	/	/	150	/	/
HSV-1-induced Cas9	pYW306	/	/	/	200	/	/	200	200
HSV-1-induced E317Ab	pYW383	/	/	/	/	200	/	200	200
SgRNA$_{US8+UL29+UL52}$	pYW102/pYW172/ pYW188	/	/	/	/	/	/	/	50 /50 /50

Amino acid or nucleic acid sequence information

Plasmid	Amino acid sequence (N → C)
pYW274<br><br>STING<br>PEST<br><br>Backbone:<br>**pcDNA3.1 (+)** (Invitrogen)	MPHSSLHPSIPCPRGHGAQKAALVLLSACLVTLWGLGEPPEHTLRYLVL<br>HLASLQLGLLLNGVCSLAEELRHIHSRYRGSYWRTVRACLGCPLRRGAL<br>LLLSIYFYYSLPNAVGPPFTWMLALLGLSQALNILLGLKGLAPAEISAV<br>CEKGNFNVAHGLAWSYYIGYLRLILPELQARIRTYNQHYNNLLRGAVSQ<br>RLYILLPLDCGVPDNLSMADPNIRFLDKLPQQTGDHAGIKDRVYSNSIY<br>ELLENGQRAGTCVLEYATPLQTLFAMSQYSQAGFSREDRLEQAKLFCRT<br>LEDILADAPESQNNCRLIAYQEPADDSSFSLSQEVLRHLRQEEKEEVTV<br>GSLKTSAVPSTSTMSQEPELLISGMEKPLPLRTDFSRIQQQLGQLTLEN<br>LQMLPESEDEESYDTESEFTEFTEDELPYDDCVFGGQR

Plasmid	Amino acid sequence (N → C)
pYW169<br><br>SEAP<br>P2A<br>Flag<br>Cas9<br><br>Backbone:<br>**pcDNA3.1 (+)** (Invitrogen)	MLLLLLLLGLRLQLSLGIIPVEEENPDFWNREAAEALGAAKKLQPAQTA<br>AKNLIIFLGDGMGVSTVTAARILKGQKKDKLGPEIPLAMDRFPYVALSK<br>TYNVDKHVPDSGATATAYLCGVKGNFQTIGLSAAARFNQCNTTRGNEVI<br>SVMNRAKKAGKSVGVVTTTRVQHASPAGTYAHTVNRNWYSDADVPASAR<br>QEGCQDIATQLISNMDIDVILGGGRKYMFRMGTPDPEYPDDYSQGGTRL<br>DGKNLVQEWLAKRQGARYVWNRTELMQASLDPSVTHLMGLFEPGDMKYE<br>IHRDSTLDPSLMEMTEAALRLLSRNPRGFFLFVEGGRIDHGHHESRAYR<br>ALTETIMFDDAIERAGQLTSEEDTLSLVTADHSHVFSFGGYPLRGSSIF<br>GLAPGKARDRKAYTVLLYGNGPGYVLKDGARPDVTESESGSPEYRQQSA<br>VPLDEETHAGEDVAVFARGPQAHLVHGVQEQTFIAHVMAFAACLEPYTA<br>CDLAPPAGTTDAAHPGYSRVGAAGRFEQTGSGATNFSLLKQAGDVEENP<br>GPSGMDYKDHDGDYKDHDIDYKDDDDKMAPKKKRKVGIHGVPAADKKYS<br>IGLDIGTNSVGWAVITDEYKVPSKKFKVLGNTDRHSIKKNLIGALLFDS<br>GETAEATRLKRTARRRYTRRKNRICYLQEIFSNEMAKVDDSFFHRLEES<br>FLVEEDKKHERHPIFGNIVDEVAYHEKYPTIYHLRKKLVDSTDKADLRL<br>IYLALAHMIKFRGHFLIEGDLNPDNSDVDKLFIQLVQTYNQLFEENPIN<br>ASGVDAKAILSARLSKSRRLENLIAQLPGEKKNGLFGNLIALSLGLTPN<br>FKSNFDLAEDAKLQLSKDTYDDDLDNLLAQIGDQYADLFLAAKNLSDAI<br>LLSDILRVNTEITKAPLSASMIKRYDEHHQDLTLLKALVRQQLPEKYKE<br>IFFDQSKNGYAGYIDGGASQEEFYKFIKPILEKMDGTEELLVKLNREDL<br>LRKQRTFDNGSIPHQIHLGELHAILRRQEDFYPFLKDNREKIEKILTFR<br>IPYYVGPLARGNSRFAWMTRKSEETITPWNFEEVVDKGASAQSFIERMT<br>NFDKNLPNEKVLPKHSLLYEYFTVYNELTKVKYVTEGMRKPAFLSGEQK<br>KAIVDLLFKTNRKVTVKQLKEDYFKKIECFDSVEISGVEDRFNASLGTY<br>HDLLKIIKDKDFLDNEENEDILEDIVLTLTLFEDREMIEERLKTYAHLF<br>DDKVMKQLKRRRYTGWGRLSRKLINGIRDKQSGKTILDFLKSDGFANRN<br>FMQLIHDDSLTFKEDIQKAQVSGQGDSLHEHIANLAGSPAIKKGILQTV<br>KVVDELVKVMGRHKPENIVIEMARENQTTQKGQKNSRERMKRIEEGIKE<br>LGSQILKEHPVENTQLQNEKLYLYYLQNGRDMYVDQELDINRLSDYDVD

	HIVPQSFLKDDSIDNKVITRSDKNRGKSDNVPSEEVVKKMKNYWRQLLN<br>AKLITQRKFDNLTKAERGGLSELDKAGFIKRQLVETRQITKHVAQILDS<br>RMNTKYDENDKLIREVKVITLKSKLVSDFRKDFQFYKVREINNYHHAHD<br>AYLNAVVGTALIKKYPKLESEFVYGDYKVYDVRKMIAKSEQEIGKATAK<br>YFFYSNIMNFFKTEITLANGEIRKRPLIETNGETGEIVWDKGRDFATVR<br>KVLSMPQVNIVKKTEVQTGGFSKESILPKRNSDKLIARKKDWDPKKYGG<br>FDSPTVAYSVLVVAKVEKGKSKKLKSVKELLGITIMERSSFEKNPIDFL<br>EAKGYKEVKKDLIIKLPKYSLFELENGRKRMLASAGELQKGNELALPSK<br>YVNFLYLASHYEKLKGSPEDNEQKQLFVEQHKHYLDEIIEQISEFSKRV<br>ILADANLDKVLSAYNKHRDKPIREQAENIIHLFTLTNLGAPAAFKYFDT<br>TIDRKRYTSTKEVLDATLIHQSITGLYETRIDLSQLGGDKRPAATKKAG<br>QAKKKK

Plasmid	Amino acid sequence (N → C)
pYW383<br><br>E317Ab<br>6×His<br>P2A<br>mCherry<br><br>Backbone:<br>**pcDNA3.1 (+)**<br>(Invitrogen)	MRTPAQFLGILLLWFPGIKCEIVLTQSPGTLSLSPGERATLSCRASQSV<br>TSSQLAWYQQKPGQAPRLLISGASNRATGIPDRFSGSGSGTDFTLTISR<br>LEPEDFAVYYCQQYGSSPTFGGGTKVEIKRAGQGSSVQVTLKQSGAEVK<br>KPGSSVKVSCTASGGTLRTYGVSWVRQAPGQGLEWLGRTIPLFGKTDYA<br>QKFQGRVTITADKSMDTSFMELTSLTSEDTAVYYCARDLTTLTSYNWWD<br>LWGQGTLVTVSHHHHHHATNFSLLKQAGDVEENPGPMVSKGEEDNMAII<br>KEFMRFKVHMEGSVNGHEFEIEGEGEGRPYEGTQTAKLKVTKGGPLPFA<br>WDILSPQFMYGSKAYVKHPADIPDYLKLSFPEGFKWERVMNFEDGGVVT<br>VTQDSSLQDGEFIYKVKLRGTNFPSDGPVMQKKTMGWEASSERMYPEDG<br>ALKGEIKQRLKLKDGGHYDAEVKTTYKAKKPVQLPGAYNVNIKLDITSH<br>NEDYTIVEQYERAEGRHSTGGMDELYKSGILQISSTVAAARV

Plasmid	Nucleic acid sequence (5' → 3')
pYW306<br><br>hIFN-RE<br>ISRE<br>P_{min}<br>SEAP<br>P2A<br>Cas9<br>SV40 polyA<br>mPGK<br>puromycin<br>E2A	CATGACAAAGGGAAACTGAAAGGGAAACTGAgatcTTAGTTTCACTTTCCCTAGTT<br>TCACTTTCCCTAGTTTCACTTTCCCAAGCTTggGctagCCCGGGCtcgaGagaggg<br>tatataatggaagctcgaattccagAagctTATACTCAGTGCCCTGACTATATACT<br>CAGTGCCCTGACTATGaattCGCCCACCATGCTGCTGCTGCTGCTGCTGGGCC<br>TGAGGCTACAGCTCTCCCTGGGCATCATCCCAGTTGAGGAGGAGAACCCGGACTTC<br>TGGAACCGCGAGGCAGCCGAGGCCCTGGGTGCCGCCAAGAAGCTGCAGCCTGCACA<br>GACAGCCGCCAAGAACCTCATCATCTTCCTGGGCGATGGGATGGGGGTGTCTACGG<br>TGACAGCTGCCAGGATCCTAAAAGGGCAGAAGAAGGACAAACTGGGGCCTGAGATA<br>CCCCTGGCCATGGACCGCTTCCCATATGTGGCTCTGTCCAAGACATACAATGTAGA<br>CAAACATGTGCCAGACAGTGGAGCCACAGCCACGGCCTACCTGTGCGGGGTCAAGG<br>GCAACTTCCAGACCATTGGCTTGAGTGCAGCCGCCCGCTTTAACCAGTGCAACACG<br>ACACGCGGCAACGAGGTCATCTCCGTGATGAATCGGGCCAAGAAAGCAGGGAAGTC<br>AGTGGGAGTGGTAACCACCACACGAGTGCAGCACGCCTCGCCAGCCGGCACCTACG

STING

PEST

BGH polyA

Backbone:

pSBtet-GP

(Addgene)

CCCACACGGTGAACCGCAACTGGTACTCGGACGCCGACGTGCCTGCCTCGGCCCGC
CAGGAGGGGTGCCAGGACATCGCTACGCAGCTCATCTCCAACATGGACATTGACGT
GATCCTAGGTGGAGGCCGAAAGTACATGTTTCGCATGGGAACCCCAGACCCTGAGT
ACCCAGATGACTACAGCCAAGGTGGGACCAGGCTGGACGGGAAGAATCTGGTGCAG
GAATGGCTGGCGAAGCGCCAGGGTGCCCGGTATGTGTGGAACCGCACTGAGCTCAT
GCAGGCTTCCCTGGACCCGTCTGTGACCCATCTCATGGGTCTCTTTGAGCCTGGAG
ACATGAAATACGAGATCCACCGAGACTCCACACTGGACCCCTCCCTGATGGAGATG
ACAGAGGCTGCCCTGCGCCTGCTGAGCAGGAACCCCCGCGGCTTCTTCCTCTTCGT
GGAGGGTGGTCGCATCGACCATGGTCATCATGAAAGCAGGGCTTACCGGGCACTGA
CTGAGACGATCATGTTCGACGACGCCATTGAGAGGGCGGGCCAGCTCACCAGCGAG
GAGGACACGCTGAGCCTCGTCACTGCCGACCACTCCCACGTCTTCTCCTTCGGAGG
CTACCCCCTGCGAGGGAGCTCCATCTTCGGGCTGGCCCCTGGCAAGGCCCGGGACA
GGAAGGCCTACACGGTCCTCCTATACGGAAACGGTCCAGGCTATGTGCTCAAGGAC
GGCGCCCGGCCGGATGTTACCGAGAGCGAGAGCGGGAGCCCCGAGTATCGGCAGCA
GTCAGCAGTGCCCCTGGACGAAGAGACCCACGCAGGCGAGGACGTGGCGGTGTTCG
CGCGCGGCCCGCAGGCGCACCTGGTTCACGGCGTGCAGGAGCAGACCTTCATAGCG
CACGTCATGGCCTTCGCCGCCTGCCTGGAGCCCTACACCGCCTGCGACCTGGCGCC
CCCCGCCGGCACCACCGACGCCGCGCACCCGGGTTACTCTAGAGTCGGGGCGGCCG
GCCGCTTCGAGCAGACAggaagcggagctactaacttcagcctgctgaagcaggct
ggagacgtggaggagaaccctggaccttccggaATGGACTATAAGGACCACGACGG
AGACTACAAGGATCATGATATTGATTACAAAGACGATGACGATAAGATGGCCCCAA
AGAAGAAGCGGAAGGTCGGTATCCACGGAGTCCCAGCAGCCGACAAGAAGTACAGC
ATCGGCCTGGACATCGGCACCAACTCTGTGGGCTGGGCCGTGATCACCGACGAGTA
CAAGGTGCCCAGCAAGAAATTCAAGGTGCTGGGCAACACCGACCGGCACAGCATCA
AGAAGAACCTGATCGGAGCCCTGCTGTTCGACAGCGGCGAAACAGCCGAGGCCACC
CGGCTGAAGAGAACCGCCAGAAGAAGATACACCAGACGGAAGAACCGGATCTGCTA
TCTGCAAGAGATCTTCAGCAACGAGATGGCCAAGGTGGACGACAGCTTCTTCCACA
GACTGGAAGAGTCCTTCCTGGTGGAAGAGGATAAGAAGCACGAGCGGCACCCCATC
TTCGGCAACATCGTGGACGAGGTGGCCTACCACGAGAAGTACCCCACCATCTACCA
CCTGAGAAAGAAACTGGTGGACAGCACCGACAAGGCCGACCTGCGGCTGATCTATC
TGGCCCTGGCCCACATGATCAAGTTCCGGGGCCACTTCCTGATCGAGGGCGACCTG
AACCCCGACAACAGCGACGTGGACAAGCTGTTCATCCAGCTGGTGCAGACCTACAA
CCAGCTGTTCGAGGAAAACCCCATCAACGCCAGCGGCGTGGACGCCAAGGCCATCC
TGTCTGCCAGACTGAGCAAGAGCAGACGGCTGGAAAATCTGATCGCCCAGCTGCCC
GGCGAGAAGAAGAATGGCCTGTTCGGAAACCTGATTGCCCTGAGCCTGGGCCTGAC
CCCCAACTTCAAGAGCAACTTCGACCTGGCCGAGGATGCCAAACTGCAGCTGAGCA
AGGACACCTACGACGACGACCTGGACAACCTGCTGGCCCAGATCGGCGACCAGTAC
GCCGACCTGTTTCTGGCCGCCAAGAACCTGTCCGACGCCATCCTGCTGAGCGACAT
CCTGAGAGTGAACACCGAGATCACCAAGGCCCCCCTGAGCGCCTCTATGATCAAGA
GATACGACGAGCACCACCAGGACCTGACCCTGCTGAAAGCTCTCGTGCGGCAGCAG
CTGCCTGAGAAGTACAAAGAGATTTTCTTCGACCAGAGCAAGAACGGCTACGCCGG
CTACATTGACGGCGGAGCCAGCCAGGAAGAGTTCTACAAGTTCATCAAGCCCATCC
TGGAAAAGATGGACGGCACCGAGGAACTGCTCGTGAAGCTGAACAGAGAGGACCTG
CTGCGGAAGCAGCGGACCTTCGACAACGGCAGCATCCCCCACCAGATCCACCTGGG

AGAGCTGCACGCCATTCTGCGGCGGCAGGAAGATTTTTACCCATTCCTGAAGGACA
ACCGGGAAAAGATCGAGAAGATCCTGACCTTCCGCATCCCCTACTACGTGGGCCCT
CTGGCCAGGGGAAACAGCAGATTCGCCTGGATGACCAGAAAGAGCGAGGAAACCAT
CACCCCCTGGAACTTCGAGGAAGTGGTGGACAAGGGCGCTTCCGCCCAGAGCTTCA
TCGAGCGGATGACCAACTTCGATAAGAACCTGCCCAACGAGAAGGTGCTGCCCAAG
CACAGCCTGCTGTACGAGTACTTCACCGTGTATAACGAGCTGACCAAAGTGAAATA
CGTGACCGAGGGAATGAGAAAGCCCGCCTTCCTGAGCGGCGAGCAGAAAAAGGCCA
TCGTGGACCTGCTGTTCAAGACCAACCGGAAAGTGACCGTGAAGCAGCTGAAAGAG
GACTACTTCAAGAAAATCGAGTGCTTCGACTCCGTGGAAATCTCCGGCGTGGAAGA
TCGGTTCAACGCCTCCCTGGGCACATACCACGATCTGCTGAAAATTATCAAGGACA
AGGACTTCCTGGACAATGAGGAAAACGAGGACATTCTGGAAGATATCGTGCTGACC
CTGACACTGTTTGAGGACAGAGAGATGATCGAGGAACGGCTGAAAACCTATGCCCA
CCTGTTCGACGACAAAGTGATGAAGCAGCTGAAGCGGCGGAGATACACCGGCTGGG
GCAGGCTGAGCCGGAAGCTGATCAACGGCATCCGGGACAAGCAGTCCGGCAAGACA
ATCCTGGATTTCCTGAAGTCCGACGGCTTCGCCAACAGAAACTTCATGCAGCTGAT
CCACGACGACAGCCTGACCTTTAAAGAGGACATCCAGAAAGCCCAGGTGTCCGGCC
AGGGCGATAGCCTGCACGAGCACATTGCCAATCTGGCCGGCAGCCCCGCCATTAAG
AAGGGCATCCTGCAGACAGTGAAGGTGGTGGACGAGCTCGTGAAAGTGATGGGCCG
GCACAAGCCCGAGAACATCGTGATCGAAATGGCCAGAGAGAACCAGACCACCCAGA
AGGGACAGAAGAACAGCCGCGAGAGAATGAAGCGGATCGAAGAGGGCATCAAAGAG
CTGGGCAGCCAGATCCTGAAAGAACACCCCGTGGAAAACACCCAGCTGCAGAACGA
GAAGCTGTACCTGTACTACCTGCAGAATGGGCGGGATATGTACGTGGACCAGGAAC
TGGACATCAACCGGCTGTCCGACTACGATGTGGACCATATCGTGCCTCAGAGCTTT
CTGAAGGACGACTCCATCGACAACAAGGTGCTGACCAGAAGCGACAAGAACCGGGG
CAAGAGCGACAACGTGCCCTCCGAAGAGGTCGTGAAGAAGATGAAGAACTACTGGC
GGCAGCTGCTGAACGCCAAGCTGATTACCCAGAGAAAGTTCGACAATCTGACCAAG
GCCGAGAGAGGCGGCCTGAGCGAACTGGATAAGGCCGGCTTCATCAAGAGACAGCT
GGTGGAAACCCGGCAGATCACAAAGCACGTGGCACAGATCCTGGACTCCCGGATGA
ACACTAAGTACGACGAGAATGACAAGCTGATCCGGGAAGTGAAAGTGATCACCCTG
AAGTCCAAGCTGGTGTCCGATTTCCGGAAGGATTTCCAGTTTTACAAAGTGCGCGA
GATCAACAACTACCACCACGCCCACGACGCCTACCTGAACGCCGTCGTGGGAACCG
CCCTGATCAAAAAGTACCCTAAGCTGGAAAGCGAGTTCGTGTACGGCGACTACAAG
GTGTACGACGTGCGGAAGATGATCGCCAAGAGCGAGCAGGAAATCGGCAAGGCTAC
CGCCAAGTACTTCTTCTACAGCAACATCATGAACTTTTTCAAGACCGAGATTACCC
TGGCCAACGGCGAGATCCGGAAGCGGCCTCTGATCGAGACAAACGGCGAAACCGGG
GAGATCGTGTGGGATAAGGGCCGGGATTTTGCCACCGTGCGGAAAGTGCTGAGCAT
GCCCCAAGTGAATATCGTGAAAAAGACCGAGGTGCAGACAGGCGGCTTCAGCAAAG
AGTCTATCCTGCCCAAGAGGAACAGCGATAAGCTGATCGCCAGAAAGAAGGACTGG
GACCCTAAGAAGTACGGCGGCTTCGACAGCCCCACCGTGGCCTATTCTGTGCTGGT
GGTGGCCAAAGTGGAAAAGGGCAAGTCCAAGAAACTGAAGAGTGTGAAAGAGCTGC
TGGGGATCACCATCATGGAAAGAAGCAGCTTCGAGAAGAATCCCATCGACTTTCTG
GAAGCCAAGGGCTACAAAGAAGTGAAAAAGGACCTGATCATCAAGCTGCCTAAGTA
CTCCCTGTTCGAGCTGGAAAACGGCCGGAAGAGAATGCTGGCCTCTGCCGGCGAAC
TGCAGAAGGGAAACGAACTGGCCCTGCCCTCCAAATATGTGAACTTCCTGTACCTG

GCCAGCCACTATGAGAAGCTGAAGGGCTCCCCCGAGGATAATGAGCAGAAACAGCT
GTTTGTGGAACAGCACAAGCACTACCTGGACGAGATCATCGAGCAGATCAGCGAGT
TCTCCAAGAGAGTGATCCTGGCCGACGCTAATCTGGACAAAGTGCTGTCCGCCTAC
AACAAGCACCGGGATAAGCCCATCAGAGAGCAGGCCGAGAATATCATCCACCTGTT
TACCCTGACCAATCTGGGAGCCCCTGCCGCCTTCAAGTACTTTGACACCACCATCG
ACCGGAAGAGGTACACCAGCACCAAAGAGGTGCTGGACGCCACCCTGATCCACCAG
AGCATCACCGGCCTGTACGAGACACGGATCGACCTGTCTCAGCTGGGAGGCGACAA
AAGGCCGGCGGCCACGAAAAAGGCCGGCCAGGCAAAAAAGAAAAAGtaaTCTAGAG
TCGGGGCGGCCGGCCGCTTCGAGCAGACATGATAAGATACATTGATGAGTTTGGAC
AAACCACAACTAGAATGCAGTGAAAAAAATGCTTTATTTGTGAAATTTGTGATGCT
ATTGCTTTATTTGTAACCATTATAAGCTGCAATAAACAAGTTAACAACAACAATTG
CATTCATTTTATGTTTCAGGTTCAGGGGGAGGTGTGGGAGGTTTTTTAAAGCAAGT
AAAACCTCTACAAATGTGGTAAAATCCGACGCGTccgggtaggggaggcgcttttc
ccaaggcagtctggagcatgcgctttagcagccccgctgggcacttggcgctacac
aagtggcctctggcctcgcacacattccacatccaccggtaggcgccaaccggctc
cgttctttggtggccccttcgcgccaccttctactcctcccctagtcaggaagttc
ccccccgccccgcagctcgcgtcgtgcaggacgtgacaaatggaagtagcacgtct
cactagtctcgtgcagatggacagcaccgctgagcaatggaagcgggtaggccttt
ggggcagcggccaatagcagctttgctccttcgctttcGaattcgccaccATGACC
GAGTACAAGCCCACGGTGCGCCTCGCCACCCGCGACGACGTCCCCAGGGCCGTACG
CACCCTCGCCGCCGCGTTCGCCGACTACCCCGCCACGCGCCACACCGTCGATCCGG
ACCGCCACATCGAGCGGGTCACCGAGCTGCAAGAACTCTTCCTCACGCGCGTCGGG
CTCGACATCGGCAAGGTGTGGGTCGCGGACGACGGCGCCGCGGTGGCGGTCTGGAC
CACGCCGGAGAGCGTCGAAGCGGGGGCGGTGTTCGCCGAGATCGGCCCGCGCATGG
CCGAGTTGAGCGGTTCCCGGCTGGCCGCGCAGCAACAGATGGAAGGCCTCCTGGCG
CCGCACCGGCCCAAGGAGCCCGCGTGGTTCCTGGCCACCGTCGGCGTCTCGCCCGA
CCACCAGGGCAAGGGTCTGGGCAGCGCCGTCGTGCTCCCCGGAGTGGAGGCGGCCG
AGCGCGCCGGGGTGCCCGCCTTCCTGGAGACCTCCGCGCCCCGCAACCTCCCCTTC
TACGAGCGGCTCGGCTTCACCGTCACCGCCGACGTCGAGGTGCCCGAAGGACCGCG
CACCTGGTGCATGACCCGCAAGCCCGGTGCCGGGTCCGGCGCTACTAACTTCAGCC
TGCTGAAGCAGGCTGGTGACGTCGAGGAGAATCCTGGTCCCatgccccactccagc
ctgcatccatccatcccgtgtcccaggggtcacggggcccagaaggcagccttggt
tctgctgagtgcctgcctggtgaccctttgggggctaggagagccaccagagcaca
ctctccggtacctggtgctccacctagcctccctgcagctgggactgctgttaaac
ggggtctgcagcctggctgaggagctgcgccacatccactccaggtaccgggggcag
ctactggaggactgtgcgggcctgcctgggctgccccctccgccgtggggccctgt
tgctgctgtccatctatttctactactccctcccaaatgcggtcggcccgcccttc
acttggatgcttgccctcctgggcctctcgcaggcactgaacatcctcctgggcct
caagggcctggccccagctgagatctctgcagtgtgtgaaaaagggaatttcaacg
tggcccatgggctggcatggtcatattacatcggatatctgcggctgatcctgcca
gagctccaggcccggattcgaacttacaatcagcattacaacaacctgctacgggg
tgcagtgagccagcggctgtatattctcctcccattggactgtggggtgcctgata
acctgagtatggctgaccccaacattcgcttcctggataaactgccccagcagacc
ggtgaccatgctggcatcaaggatcgggtttacagcaacagcatctatgagcttct

	ggagaacgggcagcgggcgggcacctgtgtcctggagtacgccaccccttgcaga<br>ctttgtttgccatgtcacaatacagtcaagctggctttagccgggaggataggctt<br>gagcaggccaaactcttctgccggacacttgaggacatcctggcagatgcccctga<br>gtctcagaacaactgccgcctcattgcctaccaggaacctgcagatgacagcagct<br>tctcgctgtcccaggaggttctccggcacctgcggcaggaggaaaggaagaggtt<br>actgtgggcagcttgaagacctcagcggtgcccagtacctccacgatgtcccaaga<br>gcctgagctcctcatcagtggaatggaaaagcccctccctctccgcacggatttct<br>ctCTGAAGAGCCACGGATTTCCCCCTGCCGTGGCTGCTCAGGGCACACTGCCCATG<br>AGCTGCGCCCAGGAGAGCGGCATGGACAGACATCCTGCCGCCTGCGCCAGCGCTAG<br>GATCAACGTGTAAACGCGTCGCAATTCTCTAGcctgcaggAGCTCGCTGATCAGCC<br>GACTGTGCCTTCTAGTTGCCAGCCATCTGTTGTTTGCCCCTCCCCCGTGCCTTCCT<br>TGACCCTGGAAGGTGCCACTCCCACTGTCCTTTCCTAATAAAATGAGGAAATTGCA<br>TCGCATTGTCTGAGTAGGTGTCATTCTATTCTGGGGGGTGGGGTGGGGCAGGACAG<br>CAAGGGGGAGGATTGGGAAGACAATAGCAGGCATGCTGGGGATGCGGTGGGCTCTA<br>TGG
Plasmid	Nucleic acid sequence (5‘ → 3‘)
pWS67<br><br>hIFN-RE<br>ISRE<br>P_{min}<br>SEAP<br><br>Backbone:<br>**pcDNA3.1 (+)**<br>(Invitrogen)	CATGACAAAGGGAAACTGAAAGGGAAACTGAGATCTTAGTTTCACTTTC<br>CCTAGTTTCACTTTCCCTAGTTTCACTTTCCCAAGCTTGGCTAGCCCG<br>GGCTCGAGAGAGGGTATATAATGGAAGCTCGAATTCCAGAagctTATAC<br>TCAGTGCCCTGACTATATACTCAGTGCCCTGACTATGaattCGCCCACC<br>ATGCTGCTGCTGCTGCTGCTGCTGGGCCTGAGGCTACAGCTCTCCCTGG<br>GCATCATCCCAGTTGAGGAGGAGAACCCGGACTTCTGGAACCGCGAGGC<br>AGCCGAGGCCCTGGGTGCCGCCAAGAAGCTGCAGCCTGCACAGACAGCC<br>GCCAAGAACCTCATCATCTTCCTGGGCGATGGGATGGGGGTGTCTACGG<br>TGACAGCTGCCAGGATCCTAAAAGGGCAGAAGAAGGACAAACTGGGGCC<br>TGAGATACCCCTGGCCATGGACCGCTTCCCATATGTGGCTCTGTCCAAG<br>ACATACAATGTAGACAAACATGTGCCAGACAGTGGAGCCACAGCCACGG<br>CCTACCTGTGCGGGGTCAAGGGCAACTTCCAGACCATTGGCTTGAGTGC<br>AGCCGCCCGCTTTAACCAGTGCAACACGACACGCGGCAACGAGGTCATC<br>TCCGTGATGAATCGGGCCAAGAAAGCAGGGAAGTCAGTGGGAGTGGTAA<br>CCACCACACGAGTGCAGCACGCCTCGCCAGCCGGCACCTACGCCCACAC<br>GGTGAACCGCAACTGGTACTCGGACGCCGACGTGCCTGCCTCGGCCCGC<br>CAGGAGGGGTGCCAGGACATCGCTACGCAGCTCATCTCCAACATGGACA<br>TTGACGTGATCCTAGGTGGAGGCCGAAAGTACATGTTTCGCATGGGAAC<br>CCCAGACCCTGAGTACCCAGATGACTACAGCCAAGGTGGGACCAGGCTG<br>GACGGGAAGAATCTGGTGCAGGAATGGCTGGCGAAGCGCCAGGGTGCCC<br>GGTATGTGTGGAACCGCACTGAGCTCATGCAGGCTTCCCTGGACCCGTC<br>TGTGACCCATCTCATGGGTCTCTTTGAGCCTGGAGACATGAAATACGAG<br>ATCCACCGAGACTCCACACTGGACCCCTCCCTGATGGAGATGACAGAGG<br>CTGCCCTGCGCCTGCTGAGCAGGAACCCCCGCGGCTTCTTCCTCTTCGT<br>GGAGGGTGGTCGCATCGACCATGGTCATCATGAAAGCAGGGCTTACCGG<br>GCACTGACTGAGACGATCATGTTCGACGACGCCATTGAGAGGGCGGCC

AGCTCACCAGCGAGGAGGACACGCTGAGCCTCGTCACTGCCGACCACTC
CCACGTCTTCTCCTTCGGAGGCTACCCCCTGCGAGGGAGCTCCATCTTC
GGGCTGGCCCCTGGCAAGGCCCGGGACAGGAAGGCCTACACGGTCCTCC
TATACGGAAACGGTCCAGGCTATGTGCTCAAGGACGGCGCCCGGCCGGA
TGTTACCGAGAGCGAGAGCGGGAGCCCCGAGTATCGGCAGCAGTCAGCA
GTGCCCCTGGACGAAGAGACCCACGCAGGCGAGGACGTGGCGGTGTTCG
CGCGCGGCCCGCAGGCGCACCTGGTTCACGGCGTGCAGGAGCAGACCTT
CATAGCGCACGTCATGGCCTTCGCCGCCTGCCTGGAGCCCTACACCGCC
TGCGACCTGGCGCCCCCCGCCGGCACCACCGACGCCGCGCACCCGGGTT
ACTCTAGAGTCGGGGCGGCCGGCCGCTTCGAGCAGACATGA

Plasmid	Nucleic acid sequence (5‘ → 3‘)
pYW414<br><br>hIFN-RE<br>ISRE<br>P_{min}<br>E317Ab<br>6×His<br>P2A<br>NanoLuc<br>IgG Fc<br>SV40 polyA<br>SV40<br>STING<br>PEST<br>SV40 polyA<br><br>Backbone:<br>**pAAV-PGK-SaCas9-bGHpA-U6-sgRNA**<br>(Addgene)	CATGACAAAGGGAAACTGAAAGGGAAACTGAgatcTTAGTTTCACTTTC<br>CCTAGTTTCACTTTCCCTAGTTTCACTTTCCCAAGCTTggGctagCCCG<br>GGCtcgaGagagggtatataatggaagctcgaattccagAagctTATAC<br>TCAGTGCCCTGACTATATACTCAGTGCCCTGACTATGgccaccatgAGG<br>ACCCCCGCTCAGTTTTTAGGAATCTTATTACTGTGGTTCCCCGGTATCA<br>AGTGCGAGATCGTGCTGACCCAGAGCCCCGGTACTTTATCTTTAAGCCC<br>CGGAGAAAGGGCCACTTTAAGCTGTCGTGCTAGCCAGAGCGTGACCTCC<br>AGCCAGCTGGCTTGGTACCAGCAGAAACCCGGTCAAGCTCCTCGTCTGC<br>TGATCAGCGGAGCCAGCAATCGTGCCACCGGCATTCCCGATCGTTTCAG<br>CGGCTCCGGCAGCGGAACCGACTTCACTTTAACCATCTCCAGACTGGAG<br>CCCGAAGACTTCGCCGTCTACTACTGCCAGCAGTACGGCAGCAGCCCTA<br>CATTCGGCGGCGGCACCAAGGTGGAGATCAAAAGGGCTGGCCAAGGTAG<br>CAGCGTGCAAGTTACACTGAAGCAGAGCGGCGCCGAGGTGAAAAAGCCC<br>GGCTCCAGCGTGAAGGTGTCTTGTACCGCTAGCGGCGGCACACTGAGGA<br>CCTACGGCGTCAGCTGGGTGAGGCAAGCTCCCGGTCAAGGTCTGGAGTG<br>GCTGGGTCGTACCATCCCTTTATTCGGCAAGACCGATTACGCCCAGAAG<br>TTCCAAGGTCGTGTGACCATCACCGCCGACAAGAGCATGGACACCTCCT<br>TCATGGAGCTGACCTCTTTAACCTCCGAGGACACCGCCGTGTACTACTG<br>CGCTAGGGATTTAACCACTTTAACCAGCTACAACTGGTGGGATTTATGG<br>GGCCAAGGCACTTTAGTGACCGTGTCCCATCATCATCATCATCATGCCA<br>CGAACTTCTCTCTGTTAAAGCAAGCAGGAGATGTTGAAGAAAACCCCGG<br>GCCTATGACTAGTGAGACAGACACACTCCTGCTATGGGTACTGCTGCTC<br>TGGGTTCCAGGTTCCACTGGTGACGCTAGTggtggttctggtATGGTCT<br>TCACACTCGAAGATTTCGTTGGGGACTGGCGACAGACAGCCGGCTACAA<br>CCTGGACCAAGTCCTTGAACAGGGAGGTGTGTCCAGTTTGTTTCAGAAT<br>CTCGGGGTGTCCGTAACTCCGATCCAAAGGATTGTCCTGAGCGGTGAAA<br>ATGGGCTGAAGATCGACATCCATGTCATCATCCCGTATGAAGGTCTGAG<br>CGGCGACCAAATGGGCCAGATCGAAAAAATTTTTAAGGTGGTGTACCCT<br>GTGGATGATCATCACTTTAAGGTGATCCTGCACTATGGCACACTGGTAA<br>TCGACGGGGTTACGCCGAACATGATCGACTATTTCGGACGGCCGTATGA<br>AGGCATCGCCGTGTTCGACGGCAAAAAGATCACTGTAACAGGGACCCTG

TGGAACGGCAACAAAATTATCGACGAGCGCCTGATCAACCCCGACGGCT
CCCTGCTGTTCCGAGTAACCATCAACGGAGTGACCGGCTGGCGGCTGTG
CGAACGCATTCTGGCGgCTAGTTCCGGTTGTAAGCCTTGCATATGTACA
GTCCCAGAAGTATCATCTGTCTTCATCTTCCCCCCAAAGCCCAAGGATG
TGCTCACCATTACTCTGACTCCTAAGGTCACGTGTGTTGTGGTAGACAT
CAGCAAGGATGATCCCGAGGTCCAGTTCAGCTGGTTTGTAGATGATGTG
GAGGTGCACACAGCTCAGACGCAACCCCGGGAGGAGCAGTTCAACAGCA
CTTTCCGCTCAGTCAGTGAACTTCCCATCATGCACCAGGACTGGCTCAA
TGGCAAGGAGTTCAAATGCAGGGTCAACAGTGCAGCTTTCCCTGCCCCC
ATCGAGAAAACCATCTCCAAAACCAAAGGCAGACCGAAGGCTCCACAGG
TGTACACCATTCCACCTCCCAAGGAGCAGATGGCCAAGGATAAAGTCAG
TCTGACCTGCATGATAACAGACTTCTTCCCTGAAGACATTACTGTGGAG
TGGCAGTGGAATGGGCAGCCAGCGGAGAACTACAAGAACACTCAGCCCA
TCATGGACACAGATGGCTCTTACTTCGTCTACAGCAAGCTCAATGTGCA
GAAGAGCAACTGGGAGGCAGGAAATACTTTCACCTGCTCTGTGTTACAT
GAGGGCCTGCACAACCACCATACTGAGAAGAGCCTCTCCCACTCTCCTG
GTAAAgCTAGCGGATCCACCGGTgTCTAGTCTAGAcagacatgataaga
tacattgatgagtttggacaaaccacaactagaatgcagtgaaaaaaat
gctttatttgtgaaatttgtgatgctattgctttatttgtaaccattat
aagctgcaataaacaagttaacaacaacaattgcattcattttatgttt
caggttcagggggaggtgtgggaggttttttaaagcaagtaaaacctct
acaaatgtggtatggGAGCTCAtgcatctcaattagtcagcaaccatag
tcccgcccctaactccgcccatcccgcccctaactccgcccagttccgc
ccattctccgccccatggctgactaattttttttatttatgcagaggcc
gaggccgcctcggcctctgagctattccagaagtagtgaggaggctttt
ttggaggcctaggcttttgcaaaaagctcctcGAATTCACgccaccatg
ccccactccagcctgcatccatccatcccgtgtcccaggggtcacgggg
cccagaaggcagccttggttctgctgagtgcctgcctggtgaccctttg
gggctaggagagccaccagagcacactctccggtacctggtgctccac
ctagcctccctgcagctgggactgctgttaaacggggtctgcagcctgg
ctgaggagctgcgccacatccactccaggtaccggggcagctactggag
gactgtgcgggcctgcctgggctgccccctccgccgtggggccctgttg
ctgctgtccatctatttctactactccctcccaaatgcggtcggcccgc
ccttcacttggatgcttgccctcctgggcctctcgcaggcactgaacat
cctcctgggcctcaagggcctggccccagctgagatctctgcagtgtgt
gaaaaagggaatttcaacgtggcccatgggctggcatggtcatattaca
tcggatatctgcggctgatcctgccagagctccaggcccggattcgaac
ttacaatcagcattacaacaacctgctacggggtgcagtgagccagcgg
ctgtatattctcctcccattggactgtggggtgcctgataacctgagta
tggctgaccccaacattcgcttcctggataaactgccccagcagaccgg
tgaccatgctggcatcaaggatcgggtttacagcaacagcatctatgag
cttctggagaacgggcagcgggcgggcacctgtgtcctggagtacgcca
ccccttgcagactttgtttgccatgtcacaatacagtcaagctggctt
tagccgggaggataggcttgagcaggccaaactcttctgccggacactt

gaggacatcctggcagatgcccctgagtctcagaacaactgccgcctca
ttgcctaccaggaacctgcagatgacagcagcttctcgctgtcccagga
ggttctccggcacctgcggcaggaggaaaggaagaggttactgtgggc
agcttgaagacctcagcggtgcccagtacctccacgatgtcccaagagc
ctgagctcctcatcagtggaatggaaaagcccctccctctccgcacgga
tttctctCTGAAGAGCCACGGATTTCCCCCTGCCGTGGCTGCTCAGGGC
ACACTGCCCATGAGCTGCGCCCAGGAGAGCGGCATGGACAGACATCCTG
CCGCCTGCGCCAGCGCTAGGATCAACGTGTAATCTAGAcagacatgata
agatacattgatgagtttggacaaaccacaactagaatgcagtgaaaaa
aatgctttatttgtgaaatttgtgatgctattgctttatttgtaaccat
tataagctgcaataaacaagttaacaacaacaattgcattcattttatg
tttcaggttcagggggaggtgtgggaggtttttt

Plasmid	Nucleic acid sequence (5‛ → 3‛)
pYWG4<br><br>hIFN-RE<br>ISRE<br>P_{min}<br>Flag tag<br>SaCa9<br>U6<br>Seed sequence of sgRNA$_{ICP4}$<br><br>Backbone:<br>**pAAV-PGK-SaCas9-bGHpA-U6-sgRNA**<br>(Addgene)	CATGACAAAGGGAAACTGAAAGGGAAACTGAgatcTTAGTTTCACTTTCCCTAGTT<br>TCACTTTCCCTAGTTTCACTTTCCCAAGCTTggGctagCCCGGGCtcgaGagaggg<br>tatataatggaagctcgaattccagAagctTATACTCAGTGCCCTGACTATATACT<br>CAGTGCCCTGACTATGaattCGCCCACCATGAATTCgccgccaccATGGACTATAA<br>GGACCACGACGGAGACTACAAGGATCATGATATTGATTACAAAGACGATGACGATA<br>AGATGGCCCCGAAGAAAAAGCGCAAGGTCGAAGCGTCCATGAAAAGGAACTACATT<br>CTGGGGCTGGACATCGGGATTACAAGCGTGGGGTATGGGATTATTGACTATGAAAC<br>AAGGGACGTGATCGACGCAGGCGTCAGACTGTTCAAGGAGGCCAACGTGGAAAACA<br>ATGAGGGACGGAGAAGCAAGAGGGGAGCCAGGCGCCTGAAACGACGGAGAAGGCAC<br>AGAATCCAGAGGGTGAAGAAACTGCTGTTCGATTACAACCTGCTGACCGACCATTC<br>TGAGCTGAGTGGAATTAATCCTTATGAAGCCAGGGTGAAAGGCCTGAGTCAGAAGC<br>TGTCAGAGGAAGAGTTTTCCGCAGCTCTGCTGCACCTGGCTAAGCGCCGAGGAGTG<br>CATAACGTCAATGAGGTGGAAGAGGACACCGGCAACGAGCTGTCTACAAAGGAACA<br>GATCTCACGCAATAGCAAAGCTCTGGAAGAGAAGTATGTCGCAGAGCTACAGCTGG<br>AACGGCTGAAGAAAGATGGCGAGGTGAGAGGGTCAATTAATAGGTTCAAGACAAGC<br>GACTACGTCAAAGAAGCCAAGCAGCTGCTGAAAGTGCAGAAGGCTTACCACCAGCT<br>GGATCAGAGCTTCATCGATACTTATATCGACCTGCTGGAGACTCGGAGAACCTACT<br>ATGAGGGACCAGGAGAAGGGAGCCCCTTCGGATGGAAAGACATCAAGGAATGGTAC<br>GAGATGCTGATGGGACATTGCACCTATTTTCCAGAAGAGCTGAGAAGCGTCAAGTA<br>CGCTTATAACGCAGATCTGTACAACGCCCTGAATGACCTGAACAACCTGGTCATCA<br>CCAGGGATGAAAACGAGAAACTGGAATACTATGAGAAGTTCCAGATCATCGAAAAC<br>GTGTTTAAGCAGAAGAAAAAGCCTACACTGAAACAGATTGCTAAGGAGATCCTGGT<br>CAACGAAGAGGACATCAAGGGCTACCGGGTGACAAGCACTGGAAAACCAGAGTTCA<br>CCAATCTGAAAGTGTATCACGATATTAAGGACATCACAGCACGGAAAGAAATCATT<br>GAGAACGCCGAACTGCTGGATCAGATTGCTAAGATCCTGACTATCTACCAGAGTTC

CGAGGACATCCAGGAAGAGCTGACTAACCTGAACAGCGAGCTGACCCAGGAAGAGA

TCGAACAGATTAGTAATCTGAAGGGGTACACCGGAACACACAACCTGTCCCTGAAA

GCTATCAATCTGATTCTGGATGAGCTGTGGCATACAAACGACAATCAGATTGCAAT

CTTTAACCGGCTGAAGCTGGTaCCAAAAAAGGTGGACCTGAGTCAGCAGAAAGAGA

TCCCAACCACACTGGTGGACGATTTCATTCTGTCACCCGTGGTCAAGCGGAGCTTC

ATCCAGAGCATCAAAGTGATCAACGCCATCATCAAGAAGTACGGCCTGCCCAATGA

TATCATTATCGAGCTGGCTAGGGAGAAGAACAGCAAGGACGCACAGAAGATGATCA

ATGAGATGCAGAAACGAAACCGGCAGACCAATGAACGCATTGAAGAGATTATCCGA

ACTACCGGGAAAGAGAACGCAAAGTACCTGATTGAAAAAATCAAGCTGCACGATAT

GCAGGAGGGAAAGTGTCTGTATTCTCTGGAGGCCATCCCCCTGGAGGACCTGCTGA

ACAATCCATTCAACTACGAGGTCGATCATATTATCCCCAGAAGCGTGTCCTTCGAC

AATTCCTTTAACAACAAGGTGCTGGTCAAGCAGGAAGAGAACTCTAAAAAGGGCAA

TAGGACTCCTTTCCAGTACCTGTCTAGTTCAGATTCCAAGATCTCTTACGAAACCT

TTAAAAAGCACATTCTGAATCTGGCCAAAGGAAAGGGCCGCATCAGCAAGACCAAA

AAGGAGTACCTGCTGGAAGAGCGGGACATCAACAGATTCTCCGTCCAGAAGGATTT

TATTAACCGGAATCTGGTGGACACAAGATACGCTACTCGCGGCCTGATGAATCTGC

TGCGATCCTATTTCCGGGTGAACAATCTGGATGTGAAAGTCAAGTCCATCAACGGC

GGGTTCACATCTTTTCTGAGGCGCAAATGGAAGTTTAAAAAGGAGCGCAACAAAGG

GTACAAGCACCATGCCGAAGATGCTCTGATTATCGCAAATGCCGACTTCATCTTTA

AGGAGTGGAAAAAGCTGGACAAAGCCAAGAAAGTGATGGAGAACCAGATGTTCGAA

GAGAAGCAGGCCGAATCTATGCCCGAAATCGAGACAGAACAGGAGTACAAGGAGAT

TTTCATCACTCCTCACCAGATCAAGCATATCAAGGATTTCAAGGACTACAAGTACT

CTCACCGGGTGGATAAAAAGCCCAACAGAGAGCTGATCAATGACACCCTGTATAGT

ACAAGAAAAGACGATAAGGGGAATACCCTGATTGTGAACAATCTGAACGGACTGTA

CGACAAAGATAATGACAAGCTGAAAAAGCTGATCAACAAAAGTCCCGAGAAGCTGC

TGATGTACCACCATGATCCTCAGACATATCAGAAACTGAAGCTGATTATGGAGCAG

TACGGCGACGAGAAGAACCCACTGTATAAGTACTATGAAGAGACTGGGAACTACCT

GACCAAGTATAGCAAAAAGGATAATGGCCCCGTGATCAAGAAGATCAAGTACTATG

GGAACAAGCTGAATGCCCATCTGGACATCACAGACGATTACCCTAACAGTCGCAAC

AAGGTGGTCAAGCTGTCACTGAAGCCATACAGATTCGATGTCTATCTGGACAACGG

CGTGTATAAATTTGTGACTGTCAAGAATCTGGATGTCATCAAAAAGGAGAACTACT

ATGAAGTGAATAGCAAGTGCTACGAAGAGGCTAAAAAGCTGAAAAAGATTAGCAAC

CAGGCAGAGTTCATCGCCTCCTTTTACAACAACGACCTGATTAAGATCAATGGCGA

ACTGTATAGGGTCATCGGGGTGAACAATGATCTGCTGAACCGCATTGAAGTGAATA

TGATTGACATCACTTACCGAGAGTATCTGGAAAACATGAATGATAAGCGCCCCCCT

CGAATTATCAAAACAATCGCCTCTAAGACTCAGAGTATCAAAAAGTACTCAACCGA

CATTCTGGGAAACCTGTATGAGGTGAAGAGCAAAAAGCACCCTCAGATTATCAAAA

AGGGCtgactcgagatgctttatttgtgaaatttgtgatgctattgctttatttgt

aaccattataagctgcaataaacaagttaacaacaacaattgcattcattttatgt
ttcaggttcagggggaggtgtgggaggttttttaaactagTGTACAAAAAAGCAGG
CTTTAAAGGAACCAATTCAGTCGACTGGATCCGGTACCAAGGTCGGGCAGGAAGAG
GGCCTATTTCCCATGATTCCTTCATATTTGCATATACGATACAAGGCTGTTAGAGA
GATAATTAGAATTAATTTGACTGTAAACACAAAGATATTAGTACAAAATACGTGAC
GTAGAAAGTAATAATTTCTTGGGTAGTTTGCAGTTTTAAAATTATGTTTTAAAATG
GACTATCATATGCTTACCGTAACTTGAAAGTATTTCGATTTCTTGGCTTTATATAT
CTTGTGGAAAGGACGAAACACCCACGTCCCCGGGGACCACGCGTTTTAGTACTCTG
GAAACAGAATCTACTAAAACAAGGCAAAATGCCGTGTTTATCTCGTCAACTTGTTG
GCGAGATTTTTTTCTAGACCCAGCTTTCTTGTACAAAGTTGGCATTAgagctcgcg
gccgc
