# Supplementary material for: Engineering antiviral immune-like systems for autonomous virus detection and inhibition in mice
Source: Nat Commun. 2022 Dec 9;13:7629. doi: 10.1038/s41467-022-35425-9 (PMC9734111; doi:10.1038/s41467-022-35425-9)
Supplement: Supplementary file 2 — Reporting Summary [file 41467_2022_35425_MOESM2_ESM.pdf]

nature portfolio

Corresponding author(s): Lin-fa Wang, Haifeng Ye
Last updated by author(s): Nov 22, 2022

Reporting Summary

Nature Portfolio wishes to improve the reproducibility of the work that we publish. This form provides structure for consistency and transparency in reporting. For further information on Nature Portfolio policies, see our Editorial Policies and the Editorial Policy Checklist.

Statistics

For all statistical analyses, confirm that the following items are present in the figure legend, table legend, main text, or Methods section.

n/a	Confirmed	
☐	☒	The exact sample size (n) for each experimental group/condition, given as a discrete number and unit of measurement
☐	☒	A statement on whether measurements were taken from distinct samples or whether the same sample was measured repeatedly
☐	☒	The statistical test(s) used AND whether they are one- or two-sided<br>*Only common tests should be described solely by name; describe more complex techniques in the Methods section.*
☒	☐	A description of all covariates tested
☒	☐	A description of any assumptions or corrections, such as tests of normality and adjustment for multiple comparisons
☐	☒	A full description of the statistical parameters including central tendency (e.g. means) or other basic estimates (e.g. regression coefficient) AND variation (e.g. standard deviation) or associated estimates of uncertainty (e.g. confidence intervals)
☐	☒	For null hypothesis testing, the test statistic (e.g. F, t, r) with confidence intervals, effect sizes, degrees of freedom and P value noted<br>*Give P values as exact values whenever suitable.*
☒	☐	For Bayesian analysis, information on the choice of priors and Markov chain Monte Carlo settings
☒	☐	For hierarchical and complex designs, identification of the appropriate level for tests and full reporting of outcomes
☒	☐	Estimates of effect sizes (e.g. Cohen's d, Pearson's r), indicating how they were calculated

Our web collection on statistics for biologists contains articles on many of the points above.

Software and code

Policy information about availability of computer code

Data collection	Cytokines, SEAP, EGFP fluorescence intensity, NanoLuc expression levels in the cell culture supernatant and mice were quantified using a Synergy H1 hybrid multi-mode microplate reader with Gen5 software (version: 2.04).
Data analysis	We used GraphPad (version 8.3.0) to perform statistical analysis.

For manuscripts utilizing custom algorithms or software that are central to the research but not yet described in published literature, software must be made available to editors and reviewers. We strongly encourage code deposition in a community repository (e.g. GitHub). See the Nature Portfolio guidelines for submitting code & software for further information.

Data

Policy information about availability of data

All manuscripts must include a data availability statement. This statement should provide the following information, where applicable:
- Accession codes, unique identifiers, or web links for publicly available datasets
- A description of any restrictions on data availability
- For clinical datasets or third party data, please ensure that the statement adheres to our policy

The data supporting the findings of this study are available within the article, in the Supplementary Information and in Source Data files. Source Data are provided with this paper.

Field-specific reporting

Please select the one below that is the best fit for your research. If you are not sure, read the appropriate sections before making your selection.

- [x] Life sciences
- [ ] Behavioural & social sciences
- [ ] Ecological, evolutionary & environmental sciences

For a reference copy of the document with all sections, see nature.com/documents/nr-reporting-summary-flat.pdf

Life sciences study design

All studies must disclose on these points even when the disclosure is negative.

Sample size	No sample-size calculation was performed. Following standards of the field, sample sizes were estimated which were capable of yielding statistically significant. Typically, 4-6 mice per group were determined by the reproducibility of nanoLuc expression.
Data exclusions	No data were excluded.
Replication	The number of independent experiments is specified in each figure legend, with at least 3 independent experiments, unless otherwise specified.
Randomization	Physical randomization. All the animals used were randomly selected from the delivered pool. Mice 6-8 weeks old were randomly selected from cages, divided into groups for the studies.
Blinding	The investigator is blinded to the group allocation and the sample at data collection.

Reporting for specific materials, systems and methods

We require information from authors about some types of materials, experimental systems and methods used in many studies. Here, indicate whether each material, system or method listed is relevant to your study. If you are not sure if a list item applies to your research, read the appropriate section before selecting a response.

Materials & experimental systems

n/a	Involved in the study	
[ ]	[x]	Antibodies
[ ]	[x]	Eukaryotic cell lines
[x]	[ ]	Palaeontology and archaeology
[ ]	[x]	Animals and other organisms
[x]	[ ]	Human research participants
[x]	[ ]	Clinical data
[x]	[ ]	Dual use research of concern

Methods

n/a	Involved in the study	
[x]	[ ]	ChIP-seq
[x]	[ ]	Flow cytometry
[x]	[ ]	MRI-based neuroimaging

Antibodies

Antibodies used	monoclonal rabbit anti-cGAS (CST, cat. no. 15102T, clone no. D1D3G, 1:1000, USA),<br>monoclonal rabbit anti-STING (CST, cat. no. 13647S, clone no. D2P2F, 1:1000, USA),<br>monoclonal mouse anti-β-Tubulin (Yesen, cat. no. 30301ES40, 1:1000, China),<br>monoclonal mouse anti-flag (Abcam, cat. no. ab125243, clone no. FG4R, 1:1000, UK),<br>monoclonal rabbit anti-GAPDH (Yesen, cat. no. 30202ES40, 1:2000, China),<br>Alexa fluor-based Goat Anti-Mouse IgG (H+L) (Yesen, cat. no. 33219ES60, 1:25,000, China),<br>Alexa fluor-based Goat Anti-Rabbit IgG (H+L) (Yesen, cat. no. 33119ES60, 1:25,000, China).
Validation	All antibodies were validated for the specified application by respective manufacturer.

Eukaryotic cell lines

Policy information about cell lines

Cell line source(s)	Human cervical adenocarcinoma cells (HeLa, ATCC: CCL-2),<br>HEK-293-derived Hana3A cells engineered for the stable expression of GαoλΦ and chaperones RTP1/RTP2/REEP1,<br>HEK-293-derived HEK-293A cells containing a stably integrated copy of the E1 gene (ThermoFisher, cat. no. R70507),<br>telomerase-immortalized human mesenchymal stem cells (hMSC-TERT, ATCC: SCRC4000),<br>human embryonic kidney cells (HEK-293T, ATCC: CRL-11268),<br>African green monkey kidney epithelium-derived Vero cells (Vero, ATCC: CCL-81),

	Vero E6 cells (ATCC no. CRL-1586), rhadomyosarcoma (RD, ATCC: CCL-136), Huh7.5.1 cells and Huh7-NTCP cells (gifted by Prof. Rongjuan Pei from Wuhan Institute of Virology, Chinese Academy of Sciences, Wuhan , Hubei, China), Aedes albopictus cells (C6/36, ATCC no. CRL-1660).
Authentication	None of the cell lines are authenticated.
Mycoplasma contamination	No mycoplasma contamination was detected for all cell lines.
Commonly misidentified lines (See ICLAC register)	No misidentified cell lines have been used in this study.

Animals and other organisms

Policy information about studies involving animals; ARRIVE guidelines recommended for reporting animal research

Laboratory animals	BALB/c mice (female, 4-week-old) were purchased from the ECNU (East China Normal University) Laboratory Animal Center.The female BALB/c wild-type mice (4-week-old; ECNU Laboratory Animal Center) were kept in an animal house maintained at 22 ± 2°C, with a 12-hour light-dark cycle and free access to food and water.
Wild animals	None.
Field-collected samples	None.
Ethics oversight	The protocol involved in this study was approved by the ECNU Animal Care and Use Committee (protocol ID: m20180403).

Note that full information on the approval of the study protocol must also be provided in the manuscript.
